# Pilot experience with opebacan/rBPI _21_ in myeloablative hematopoietic cell transplantation

**DOI:** 10.12688/f1000research.7558.1

**Published:** 2015-12-21

**Authors:** Eva Guinan, David E Avigan, Robert J Soiffer, Nancy J Bunin, Lisa L Brennan, Ilana Bergelson, Spencer Brightman, Al Ozonoff, Patrick J Scannon, Ofer Levy

**Affiliations:** 1 Dana-Farber Cancer Institute, Boston, USA; 2 Harvard Medical School, Boston, USA; 3 Boston Children's Hospital, Boston, USA; 4 Beth Israel Deaconess Medical Center, Boston, USA; 5 Brigham and Women's Hospital, Boston, USA; 6 Children's Hospital of Philadelphia, Philadelphia, USA; 7 Xoma (US) LLC, Berkeley, USA

**Keywords:** endotoxin, innate immunity, allogeneic hematopoietic cell transplantation, regimen-related toxicity, infection, engraftment

## Abstract

Bacterial infection and inflammation contribute significantly to the morbidity and mortality of myeloablative allogeneic hematopoietic cell transplantation (HCT). Endotoxin, a component of the outer membrane of Gram-negative bacteria, is a potent inflammatory stimulus in humans. Bactericidal/permeability increasing protein (BPI), a constituent of human neutrophil granules, binds endotoxin thereby precluding endotoxin-induced inflammation and also has direct anti-infective properties against bacteria. As a consequence of myeloablative therapy used in preparation for hematopoietic cell infusion, patients experience gastrointestinal leak of bacteria and bacterial toxins into the systemic circulation and a period of inflammatory cytokine elevation associated with subsequent regimen-related toxicities.  Patients frequently become endotoxemic and febrile as well as BPI-deficient due to sustained neutropenia. To examine whether enhancing endotoxin-neutralizing and anti-infective activity by exogenous administration of a recombinant N-terminal fragment of BPI (rBPI _21_, generic name opebacan) might ameliorate regimen-related toxicities including infection, we recruited patients scheduled to undergo myeloablative HCT to participate in a proof-of-concept prospective phase I/II trial. After the HCT preparative regimen was completed, opebacan was initiated 18-36 hours prior to administration of allogeneic hematopoietic stem cells (defined as Day 0) and continued for 72 hours. The trial was to have included escalation of rBPI _21 _dose and duration but was stopped prematurely due to lack of further drug availability.  Therefore, to better understand the clinical course of opebacan-treated patients (n=6), we compared their outcomes with a comparable cohort meeting the same eligibility criteria and enrolled in a non-interventional myeloablative HCT observational study (n = 35).  Opebacan-treated participants had earlier platelet engraftment (p=0.005), mirroring beneficial effects of rBPI _21_ previously observed in irradiated mice, fewer documented infections (p=0.03) and appeared less likely to experience significant regimen-related toxicities (p=0.05). This small pilot experience supports the potential utility of rBPI _21_ in ameliorating HCT-related morbidity and merits further exploration.

## Introduction

Regimen-related toxicities, including infection, organ damage, and acute graft-versus-host disease (aGvHD), remain significant barriers to successful allogeneic hematopoietic cell transplantation (HCT). The “cytokine storm” hypothesis - that regimen-related injury to host cells creates a pro-inflammatory environment contributing to aGVHD and other toxicities – has been well substantiated in various experimental models ^1, 2^. One consequence of such host cell injury, specifically gastrointestinal (GI) damage from myeloablative therapy, results in leakage of bacterial lipopolysaccharide (LPS), also referred to as endotoxin, into the systemic circulation in both mice and humans undergoing HCT ^3, 4^. LPS, which is a constituent of the outer membrane of Gram-negative bacteria ^5^ is one of the most potent inflammatory stimuli in humans, and LPS-induced production of pro-inflammatory and Th1-polarizing cytokines has been linked to subsequent aGvHD in model systems ^6– 8^. Moreover, administration of a synthetic LPS antagonist for 6 days starting from the day of transplantation reduced TNFα production, intestinal damage, aGvHD and mortality after murine myeloablative HCT while preserving the graft vs. leukemia (GvL) effect ^7, 9^.

Upon entry into the systemic circulation, LPS is recognized by proteins that enhance its activity by shepherding LPS to its major tripartite pro-inflammatory cell surface receptor composed of *Toll*-like receptor 4 (TLR4), CD14 and MD-2 ^10– 13^. This multi-step delivery system amplifies the effect of small amounts of LPS. Given the resulting potency of LPS, there are also numerous mechanisms, including clearance, detoxification and neutralization, that decrease LPS-mediated inflammation ^10– 13^. Antimicrobial proteins and peptides (APPs) associated with neutrophil granules provide a potent source of LPS neutralizing activity ^14^. One of these granule constituents, BPI, is a cationic 55 kDa protein with high affinity for the lipid A region common to most LPS variants ^15, 16^. Among APPs, including defensins, BPI is a particularly potent LPS inhibitor active at nanomolar concentrations ^17^. Binding of BPI to LPS precludes LPS binding to both lipopolysaccharide-binding protein (LBP) and LPS receptors such as TLR4, thus inhibiting LPS-induced inflammation, including TNFα production ^18^.

Recombinant N-terminal fragments of BPI (including rBPI _23_ and rBPI _21_ [opebacan, NEUPREX ^®^]) possessing the LPS-binding and LPS-neutralizing activity of native BPI were developed by XOMA (US) LLC (Berkeley, CA) as anti-infective agents for use in sepsis and other infectious disease indications. These BPI congeners have potent *in vitro* endotoxin-neutralizing activity ^15^ and have demonstrated efficacy in multiple animal models of endotoxemia ^19– 23^. In human trials, intravenous (IV) administration of either rBPI _23_ or rBPI _21_ has appeared safe and non-immunogenic ^24– 27^ and ameliorated LPS-induced changes in parameters including cardiac index, cytokine release and coagulation. rBPI _21_ and rBPI _23_ have bactericidal activity and exhibit synergy with conventional antibiotics, including activity against antibiotic-resistant bacteria ^18, 28^.

We have proposed that opebacan may be most beneficial for individuals deficient in endogenous BPI ^29^. Myeloablative HCT, during which recipients experience simultaneous endotoxemia and neutropenia ^3, 4, 30^, represents a condition where the LPS:BPI ratio is high. To further pursue the hypothesis that providing additional LPS-neutralizing activity would abrogate LPS-related toxicity when systemic LPS is present and endogenous BPI is inadequate, we undertook a Phase I/II study of opebacan during myeloablative HCT to investigate safety and preliminary correlative clinical and laboratory data strategic decisions related to inflammation and regimen-related toxicity. The sponsor prematurely discontinued the study because of strategic decisions related to unanticipated insufficient drug supply. In order to generate hypotheses for future work and to begin to describe the effects of rBPI _21_ in this setting, we therefore compared the outcomes of the completed cohort of opebacan-treated participants with those of individuals meeting the same eligibility criteria and enrolled in a non-interventional study of innate immunity after myeloablative HCT.

## Material and methods

***Study design and eligibility.*** From 9/2007-7/2008, sequential eligible patients scheduled to undergo allogeneic HCT were offered participation ( SM Figure 1) in an open-label, dose-finding study of opebacan ( NCT00454155) at the Children’s Hospital of Philadelphia (protocol CHP 871), the Beth Israel Deaconess Medical Center (BIDMC) and the Dana-Farber Cancer Institute/Brigham and Women’s Hospital (DFCI, BWH) (all protocol 06155). Eligibility criteria included: use of a myeloablative regimen (total body irradiation [TBI] ≥1000 cGy or busulfan ≥14 mg/kg PO or IV equivalent); Lansky or Karnofsky performance score >80%; first HCT; no active infection. Additional criteria included: room air oxygen saturation >95%; serum creatinine <1.5× upper limit of normal (ULN); AST and ALT ≤3×ULN and total bilirubin ≤1.5×ULN; normal cardiac shortening or ejection fraction; no history of congestive heart failure; normal cardiac troponin T level; cumulative anthracycline exposure <300 mg/m ^2^. Exclusion criteria included: use of cord blood cells; T-cell depletion regimen; prophylactic antibiotics beyond standard practice; or planned heparin anticoagulation or dextran sulfate use (both antagonize opebacan activity) during opebacan infusion. The study was originally open to those <60 years and modified to have a lower limit of >18 years. The study was approved by the DFCI and the Children’s Hospital of Philadelphia Institutional Review Boards (IRBs). All participants and/or legal guardians gave written consent and/or assent.

The original interventional study design included 5 cohorts (each n=6) with sequential escalation of opebacan dose and duration ( Supplementary material: Figure 2 and Opebacan trial protocol 06155). Opebacan was administered via central IV catheter after completion of the myeloablative regimen and ≥18 and ≤36 hours prior to donor cell infusion. In order to reach a steady-state plasma level rapidly, an initial bolus was followed by continuous IV infusion.

The protocol anticipated comparisons between 5 cohorts. However, the study was discontinued after the first cohort based on a strategic manufacturing decision by the sponsor that precluded sufficient drug availability to complete planned accrual. To better understand the outcomes of cohort 1, we therefore identified a comparison group (COMP, n=35) enrolled on a non-interventional, sample collection study of endotoxin-related innate immunity after HCT who met eligibility criteria for the opebacan trial. COMP participants had been recruited prospectively from 8/2005-7/2009 at Boston Children’s Hospital (BCH), DFCI and BWH onto protocol 05127 ( Supplementary material: Comparison group protocol 05127), approved by the DFCI IRB. Comparison of the two data sets was IRB approved. All participants and/or legal guardians gave written consent and/or assent.

Patient characteristics are shown in Table 1. Endpoints were defined according to the opebacan protocol ( Supplementary material: Opebacan trial protocol 06155). All tests were conducted in clinical laboratories per routine. Day 0 was defined as the day of cell infusion. Engraftment was defined as the first of 3 consecutive days with absolute neutrophil count (ANC) of ≥500/µL. Platelet recovery was defined as the first of 7 consecutive days with untransfused platelet count ≥20,000/µL. The maximal temperature ± 1 day of sample acquisition was recorded. Supportive care included acyclovir prophylaxis in herpes simplex sero-positive individuals, quantitative polymerase chain reaction (PCR) screening for cytomegalovirus (CMV) reactivation followed by treatment if indicated, and antifungal prophylaxis. Participants received “gut decontamination” consisting either of oral non-absorbable antibiotics or oral or intravenous fluoroquinolone, per the treating medical team. aGVHD was graded according to modified Glucksberg ^31^. Per FDA practice, toxicity was recorded for 30 days after drug completion (rounded to Day 35 post-HCT) on case report forms. Per institutional routine, toxicity data were collected through Day 100. Maximal severity of adverse events (AEs) was reported per NCI Common Terminology Criteria for Adverse Events (CTCAE), version 3.0. Infections were defined by positive blood culture; focal complaint or finding with positive culture from a normally sterile or clinically indicated site; or other confirmatory laboratory evaluation (e.g., PCR). Absent infection, a fever occurring during neutropenia was classified as febrile neutropenia. CMV viremia on routine screening absent a clinical complaint or CMV-relatable organ disease was denoted CMV reactivation. Biopsy-confirmed CMV was denoted CMV infection. Focal physical and/or radiologic findings without microbiological confirmation were scored culture-negative (i.e., possible) infections. *Clostridium difficile* in stool, as it is frequently present on HCT admission, was excluded from the infection analysis. Research staff with HCT expertise abstracted data.

**Table 1.** Participant characteristics.

Characteristics	Opebacan Study	Comparison Study	p-value*
	N (%)	N (%)	
Participants enrolled	6	35	
Age in years	17–55 (median 50)	17–60 (median 43)	p=0.16
Gender: M/F	2/4	21/14	p=0.38
Diagnosis:			p=1.00
Acute leukemia	3 (50)	17 (48)	
MDS/ Myelofibrosis	1	6	
CML	2	6	
Lymphoma	-	4/1	
CLL	-	1	
Conditioning:			p=0.58
TBI/CY	4 (67)	29 (82)	
BU/CY	2	6	
Stem Cell Source:			
Related: Unrelated	3:3 (50)	25:10 (71)	p=0.36
PBSC	4 (67)	29 (83)	p=0.58
BM	2	5	
BM+PBSC	0	1	
GVHD prophylaxis:			p=1.00
MTX/CI	6	31	
Sirolimus/ Tacrolimus	-	4	

Abbrev. MDS, myelodysplasia; CML, chronic myelogenous leukemia; CLL, chronic lymphocytic leukemia; TBI/CY, total body irradiation/Cyclophosphamide; BU/CY, Busulfan/Cyclophosphamide;

PBSC, peripheral blood stem cells; BM, bone marrow; GVHD, graft vs. host disease;

CI, calcineurin inhibitors; MTX, methotrexate;

*All p-values are Fisher's exact test except for age, which is derived by Wilcoxon Rank Sum.

## Statistical analysis

We generated summary statistics of sample characteristics and outcomes for both cohorts. For between-group comparisons of proportion of subjects with toxicity, we used Fisher’s exact test with exact p-values calculated directly from the hypergeometric distribution. For comparisons of toxicity rates we used Poisson regression with a generalized linear model with log (patient days) as offset and a single binary predictor for cohort. We present the p-value for the cohort effect i.e. the estimate divided by the standard error and fit to the standard normal. We used the Mantel-Cox log rank test for event rates and the Wilcoxon rank sum test for the age comparison. The software was the R statistical package v3.1.0 (R Institute, Vienna, 2014). In Table 1, the diagnosis of acute leukemia was compared to all other diagnoses and for stem cell source, PBSC were compared to BM and BM plus PBSC.

## Results

Opebacan-treated participant demographic, and transplant information as well as transplant-related toxicity Click here for additional data file. Copyright: © 2015 Guinan E et al. 2015 Data associated with the article are available under the terms of the Creative Commons Zero "No rights reserved" data waiver (CC0 1.0 Public domain dedication).

COMP cohort participant demographic, and transplant information as well as transplant-related toxicity Click here for additional data file. Copyright: © 2015 Guinan E et al. 2015 Data associated with the article are available under the terms of the Creative Commons Zero "No rights reserved" data waiver (CC0 1.0 Public domain dedication).

***Participant characteristics.*** Thirty sequential HCT candidates were screened for the opebacan interventional study. Of these, 24 were ineligible [non-myeloablative regimen (n=13), excessive anthracycline exposure (n=8) and enrollment on another trial (n=3)]. Six eligible patients enrolled. All completed the planned treatment. Of 54 individuals enrolled on the innate immunity observational study, 19 were ineligible for the opebacan trial and were excluded from the COMP cohort. Reasons for exclusion included: age (n=10), non-myeloablative regimen (n=5), relapse prior to HCT (n=2), and HCT cancellation (n=2). The remaining 35 constituted the COMP group. Both groups had complete follow-up to death or Day 100. Participant characteristics in both groups were similar with respect to age, gender, diagnosis, stem cell source and GVHD prophylaxis ( Table 1). Conditioning in both groups favored TBI-containing regimens. All but one participant had an HLA matched donor (100% opebacan vs 97% COMP).

***Opebacan treatment trial.*** Six participants were enrolled from 3 institutions into cohort 1. All received opebacan per protocol with 2 deviations [drug interrupted inadvertently for 30 minutes on treatment day 1 (1 patient) and drug discontinued 1 hour prematurely (1 patient)]. There were no infusional toxicities or serious adverse events (SAEs) related to study drug. Per the Investigator’s Brochure, cardiotoxicity had rarely been observed in animal studies examining higher doses and longer durations of rBPI _21_ than prescribed here. This had not been observed in prior clinical trials of rBPI congeners, including opebacan ^24– 27, 32^. Here, all participants had normal shortening or ejection fractions prior to study entry, and none developed clinical evidence of cardiotoxicity during infusion or thereafter. Participants had echocardiograms weekly through Day 30 or hospital discharge and again at Day 100. One participant (aged 17) had a shortening fraction decrease at Day 100 read by the echocardiographer as “mild left ventricular dysfunction”. This participant had no cardiac findings at last follow-up. The remaining 5 participants had normal ejection (measured in 5 of 5) and shortening (measured in 4 of 5) fractions at all time-points.

***Clinical course.*** All opebacan and COMP participants had neutrophil engraftment (median Day 17 [range 14–28] vs Day 14 [range 10–30], respectively; p=0.35; Table 2). All participants met platelet engraftment criteria, with the exception of one COMP participant who died prior to achieving this endpoint. Opebacan-treated individuals had significantly more rapid platelet engraftment (median 12 days, range 10–26) than observed in COMP participants (median 19 days, range 13–109; p=0.005; Figure 1). There was no severe (Grade III/IV) aGVHD observed in opebacan participants. Grade III/IV aGVHD was observed in 4/35 (11%) of COMP participants.

**Table 2.** All regimen-related toxicities.

	Opebacan Study	Comparison Study	p-Value*
	N	N	
# Enrolled	6	35	
Total Patient Days per interval D0–35/D36–100	210/390	1225/1931	
Days to engraftment (median/ range)			
ANC	17 (14–28)	14 (10–30)	p=0.35
PLT	12.5 (10–26)	19 (13–109)	p=0.005
Acute GVHD	Grade 0 = 1	Grade 0 =17	p=0.81
	Grade 1 = 1	Grade 1 = 8	p=0.45
	Grade 2 = 4	Grade 2 = 6	p=0.21
	Grade 3 = 0	Grade 3 = 2	N/A
	Grade 4 = 0	Grade 4 = 2	N/A
**Grade 3 toxicity ≤ day 35**			
# patients	4	33	p=0.095
# and type of toxicities	10	75	p=0.46
	hypertension, mucositis, F/N, cellulitis at line site	hypertension, mucositis, F/N, pleural effusion, hepatitis, pneumonitis, DVT, VOD, infection	.
**Grade 4 toxicity ≤ day 35**			
# patients	0	6	p=0.57
# and type of toxicities	0	8	N/A
	-	mucositis, infection, non-VOD hyperbilirubinemia	
Grade 3–5 toxicity ≤ day 35**			
# patients	4	34	p=0.05
# toxicities	10	83	p=0.29
**Grade 3 toxicity day 36–100**			
# patients	1	6	p=1.00
# and type of toxicities	2	7	p=0.52
	infection	infection	
**Grade 4 toxicity day 36–100**			
# patients	0	3	p=1.00
# and type of toxicities	0	3	N/A
	-	infection, respiratory failure	
**Grade 5 toxicity day 36–100**			
# patients	0	4	p=1.00
# toxicities	0	4	N/A
day of death/cause	-	D39 – DAH; D40 – VOD; D71 – ARDS; D78 – RF	
**Grade 3–5 toxicity day 36–100**			
# patients	1	10	p=1.00
# toxicities	2	14	p=0.65
**100 day mortality**	0 (0)	4/35 (11%)	p=1.00

Abbr: ANC, absolute neutrophil count; PLT, platelet, F/N= febrile neutropenia; DVT, deep vein thrombosis; RF, renal failure; VOD, veno-occlusive disease of liver; ARDS, acute respiratory distress syndrome; DAH, diffuse alveolar hemorrhage.

* All p-values determined by Fisher's exact test except for ANC and PLT which are by Mantel-Cox log rank.

**No grade 5 toxicity in either group ≤D 35.

**Figure 1.**
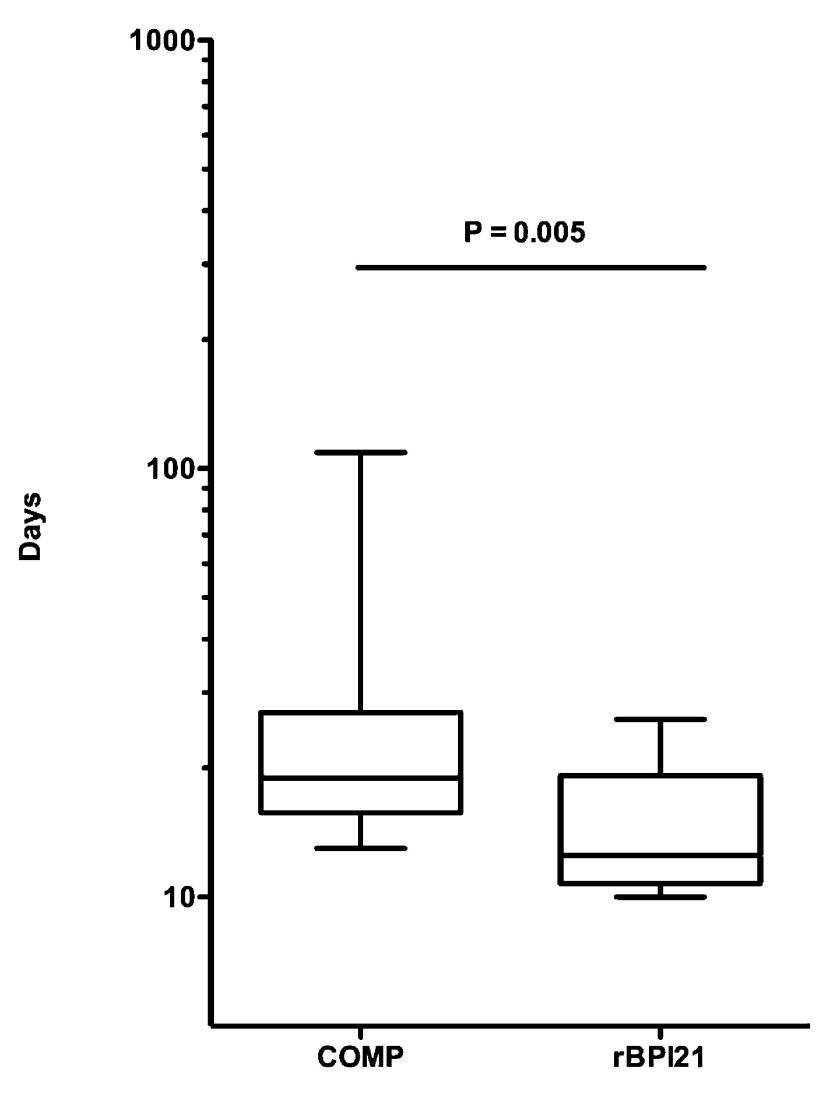
Opebacan-treated patients undergoing myeloablative HCT demonstrated more rapid platelet engraftment than the comparator group. Depicted is a box and whisker plot of the median (horizontal bar) and quartiles (bottom and top of boxes; 25 ^th^ to 75 ^th^) for days to platelet engraftment (as defined in Methods) in opebacan treated and COMP participants. The opebacan treated group demonstrated more rapid platelet engraftment by Mantel-Cox log rank test; p=0.005.

***Regimen-related toxicity.*** During the first 35 days, 4 opebacan-treated participants experienced at least one grade 3 toxicity for 10 aggregate grade 3 toxicities (4.8 toxicities/100 participant days at risk, Table 2). In the COMP group, we observed at least one grade 3 toxicity in 33 participants for 75 aggregate grade 3 toxicities (6.1 toxicities/100 participant days at risk). No grade 4 (life-threatening) toxicities were observed in the opebacan group in contrast to at least one grade 4 toxicity in 6 COMP participants (17%) for 8 aggregate grade 4 toxicities (0.7 toxicities/100 participant days at risk). No grade 5 toxicities were observed in either group. Thus, opebacan-treated participants experienced a lower rate of grade 3–5 toxicity through Day 35 than the COMP group (4/6 participants vs 34/35, respectively; p=0.05). In total, 10 (or 4.8/100 participant days at risk) and 83 (or 6.8/100 participant days at risk) grade 3–5 toxicities were observed in the opebacan and COMP groups, respectively (p=0.29).

To characterize the complete period for which acute HCT toxicities are commonly reported, we also analyzed toxicities during Day 36–100. During this period, we observed 2 and 7 grade 3 toxicities in the treatment and COMP groups, respectively ( Table 2). Opebacan-treated participants experienced no grade 4–5 toxicities. Three COMP participants (9%) each experienced one grade 4 toxicity and 4 experienced a fatal (grade 5) toxicity. Thus, we observed 0.51 and 0.73 grade 3–5 toxicities/100 participant days at risk in the opebacan and COMP groups, respectively (p=0.65).

***Infection.*** We first examined the incidence of possible or proven infection during the period of opebacan administration. One participant (not neutropenic) in the opebacan-treated group had fever (Day -1; 16.7%). Five of 35 COMP participants developed fever (median day +1; 14.3%) during this time, of whom 3 were not neutropenic and one had bacteremia meeting sepsis criteria. No other sources for fever were identified. There was no statistically significant difference in occurrence of early fever.

We next examined all regimen-related toxicities classified as infections ( Table 3). As opebacan was administered for only 72 hours peri-transplant, we again examined the early and later post-HCT intervals separately. Through Day 35 post-HCT, COMP participants were more likely to have positive cultures than those treated with opebacan (p=0.03). One opebacan participant had possible infection based on CVL-insertion site erythema and tenderness, but cultures were negative. In contrast, 6 possible (culture-negative) and 18 documented infections occurred in the COMP group. Of these, 14 were bacterial, including 10 bacteremias of which 4 fulfilled criteria for sepsis, 1 fungal and 3 viral.

**Table 3.** Infections.

	Opebacan Cohort	Comparison Cohort	p-value
	N	N	(Fisher's/GLM)
Patients	6	35	
**Days 0–35**			
Culture Positive (Total)	0	18	p=0.03
Bacterial	0	14	p=0.08
		Enterococcus*# S. epidermidis*, S. aureus*#, Streptococcus mitis*	
Fungal	0	1	p=1.00
		Neosartorya species*	
Viral	0	3	p=1.00
		CMV*+	
Culture negative (Total)	1	6	p=1.00
**Days 36–100**			
Culture Positive (Total)	2	9	p=0.65
Bacterial	1	5	p=1.00
	S.epidermidis*#	S. epidermidis*, S. aureus*, Enterococcus*, E. coli ^#^	
Fungal	0	0	-
Viral	1	4	p=0.57
	HSV^	CMV* ^$^, parainfluenza ^%^	
Culture negative (Total)	0	0	-

Abbrev. CMV, Cytomegalovirus; HSV-Herpes simplex virus; *E.coli, Escherichia coli; S. aureus, Staphylococcus aureus; S.epidermidis, Staphylococcus epidermidis.*

* blood; # urine; + broncheolar lavage, ^ oral swab; $ colon biopsy, % nasopharynx swab

In the Day 36–100 interval ( Table 3), one opebacan participant developed concurrent bacteremia and urinary tract infection as well as oral HSV. COMP participants experienced 9 confirmed infections. Five were bacterial, including 4 bacteremias of which 2 fulfilled criteria for sepsis. There were also 4 viral infections. There was no statistically significant difference in infection incidence (p=0.65).

## Discussion

Here we report the first experience of rBPI _21_/opebacan administration to humans undergoing myeloablative allogeneic HCT. The drug appeared well tolerated, without attributable SAEs. Time to engraftment, incidence of regimen-related toxicities, including infection, and aGVHD appeared equivalent to or better than a cotemporaneous comparison cohort meeting the opebacan trial eligibility criteria.

We have published that administration of rBPI _21_ and daily enrofloxacin (a veterinary ciprofloxacin equivalent) is associated with significantly increased survival and accelerated hematopoietic recovery in a murine model of myeloablative TBI mimicking unintended radiation exposure (e.g. after a nuclear event) ^30^. In the murine model, single-fraction TBI was given, subcutaneous rBPI _21_ treatment was initiated 24 hours after TBI, and no stem cells were administered to restore hematopoiesis. While there are significant differences between the murine TBI model and human HCT, the rapid hematopoietic reconstitution observed in rBPI _21_-treated irradiated mice prompted our interest in the potential effects of rBPI _21_ on engraftment. Consistent with the effects observed in the murine TBI model, we found the median time to platelet engraftment was significantly decreased (by one week) in opebacan-treated participants. Time to neutrophil engraftment was similar despite greater use of peripheral blood stem cells, which are associated with more rapid neutrophil and platelet engraftment ^33, 34^, in COMP participants (86% vs 67% in opebacan participants). However, these are small groups and these results require confirmation.

Infection is one of the most common toxicities experienced by patients undergoing myeloablative HCT and one of, if not the major, contributors to non-relapse mortality ^35, 36^. Documented infections in patients undergoing myeloablative HCT are largely Gram-positive ^35, 36^ as was observed here ( Table 3). While both native BPI and rBPI _21_ have well-recognized anti-infective activities toward Gram-negative bacteria ^37^, they also bind to and contribute anti-infective activities against Gram-positive organisms ^38, 39^. Relevant mechanisms of action in both settings include membrane permeabilization, bacterial-toxin binding, facilitation of phagocytic opsonization and effects on membrane polarization ^37– 39^. Cell wall-deficient (e.g. L-form) bacteria, which may contribute to culture-negative fevers in HCT patients, are also susceptible to BPI-mediated killing ^32, 40^. Overall, the lower infection incidence observed in opebacan-treated participants appears consistent with BPI’s antimicrobial properties as well as associations of BPI gene single nucleotide polymorphisms with infection risk after myeloablative HCT ^41^.

Opebacan treatment was associated with less early, significant regiment-related toxicity (p=0.05). In addition, the opebacan group experienced only common regimen-related toxicities, such as febrile neutropenia and mucositis. In contrast, the toxicities noted in the COMP group included well-known but less frequent and more serious post-HCT complications such as hepatic veno-occlusive disease, pneumonitis and pleural effusions. Interestingly, low level endotoxemia has been associated with greater degrees of organ dysfunction in several other settings, including congestive heart failure, renal failure, and HIV infection ^42– 44^. Moreover, lower levels of BPI in neutrophils have been associated with atherosclerotic disease severity ^45^, and BPI polymorphisms have been associated with bronchiolitis obliterans, a devastating HCT complication ^46^. In murine allogeneic HCT models, endotoxin has been shown to play a significant role in the incidence of aGVHD and regimen-related toxicity, and therapies mitigating endotoxin-mediated effects have decreased such effects ^7– 9^. Our preliminary observations coupled with the literature thus support the hypothesis that rBPI _21_-mediated endotoxin neutralization may limit the occurrence and severity of toxicities in human myeloablative HCT.

Although the findings reported here are novel and support the study hypotheses, several substantial limitations pertain. The number of subjects is small. In addition, while the groups compared above met the same eligibility criteria and underwent HCT during a similar timeframe, this was not a prospective randomized trial and there were no formal power calculations performed for the newly constituted comparison of outcomes. Finally, we were unable to complete the original trial of increasing opebacan dose and duration due to long-term disruption of drug supply. Given that rBPI _21_ has concentration-dependent effects ^28^, it is possible and perhaps likely that greater benefit would be observed with prolonged and/or higher dosing. These results suggest that opebacan can be safely administered to individuals undergoing myeloablative HCT. In addition, positive trends or significant findings with respect to time to platelet engraftment, and the incidence of regimen-related toxicity and infection are consistent with beneficial anti-infective, systemic and hematopoietic effects of BPI described previously. This pilot experience suggests that further investigation of the potential role of opebacan in mitigating toxicity and infection in this and other clinical settings characterized by reduced neutrophil quantity or quality is warranted.

## Data availability

The data referenced by this article are under copyright with the following copyright statement: Copyright: © 2015 Guinan E et al.

Data associated with the article are available under the terms of the Creative Commons Zero "No rights reserved" data waiver (CC0 1.0 Public domain dedication). 

*F1000Research*: Dataset 1. Opebacan-treated participant demographic, and transplant information as well as transplant-related toxicity, 10.5256/f1000research.7558.d109174 ^47^

*F1000Research*: Dataset 2. COMP cohort participant demographic, and transplant information as well as transplant-related toxicity, 10.5256/f1000research.7558.d109175 ^48^

## Consent

Written informed consent for participation including publication of deidentified information was obtained from all participants.

## Figures and Tables

**Figure 1.**
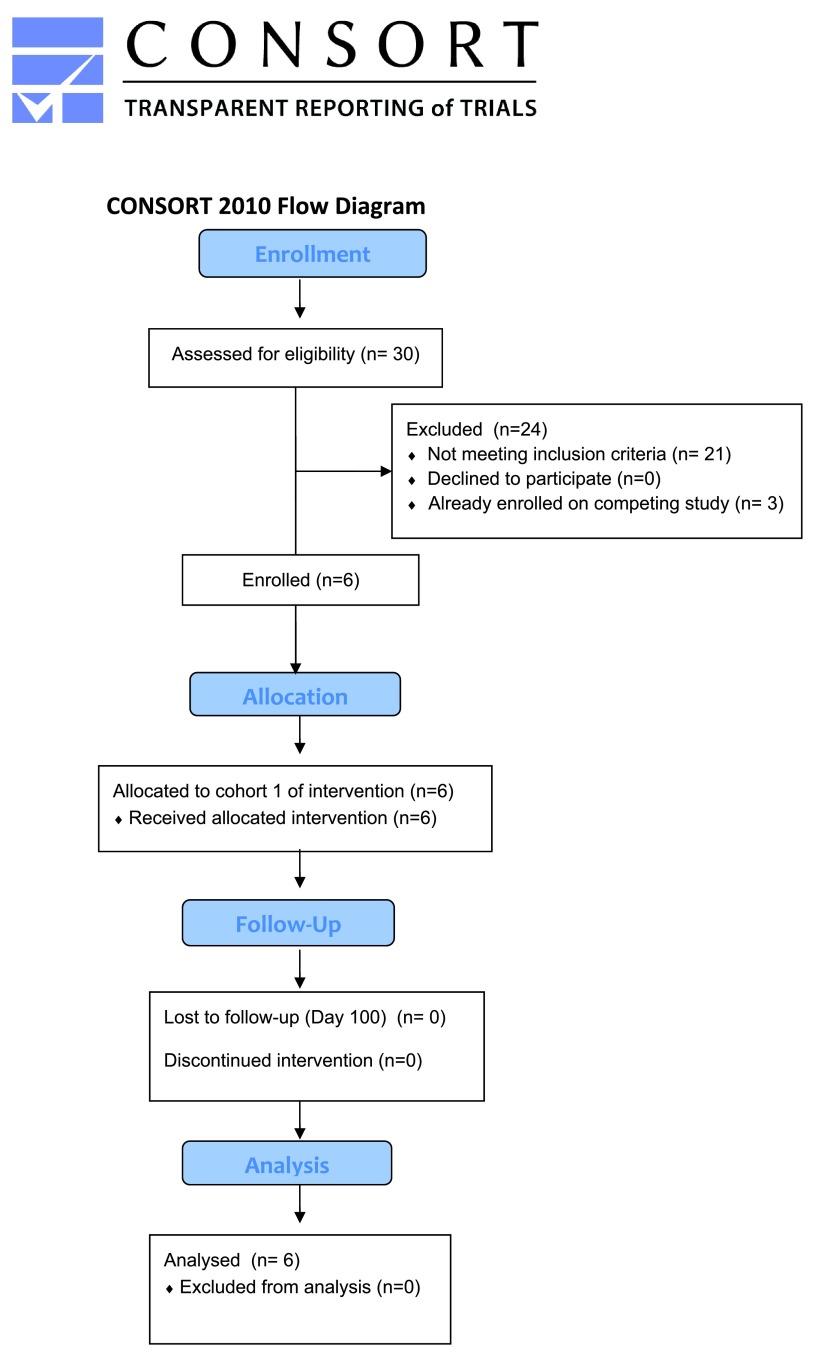
Clinical trial participant flow diagram (CONSORT). Recruitment, enrollment and subsequent flow through the study for the clinical trial cohort (n=6) is shown.

**Figure 2.**
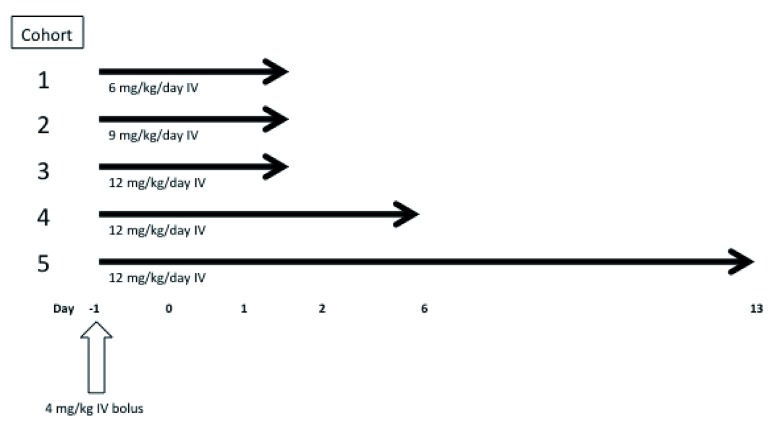
Treatment schema of phase I/II opebecan (rBPI _21_) study in patients undergoing myeloablative HCT. The study design included an IV bolus dose of 4mg/kg followed by daily IV administration of escalating doses (Cohorts 1–3) and extension of duration (Cohorts 4 and 5). Due to discontinuation of drug availability, only cohort 1 (i.e., 4 mg/kg IV bolus followed by 6mg/kg/day IV) was enrolled and completed. Click here for additional data file.. Click here for additional data file..
