# Supplementary material for: Pilot experience with opebacan/rBPI 21 in myeloablative hematopoietic cell transplantation
Source: F1000Res. 2015 Dec 21;4:1480. [Version 1] doi: 10.12688/f1000research.7558.1 (PMC4722698; doi:10.12688/f1000research.7558.1)
Supplement: Supplementary file 3 [file f1000research-4-8138-s0002.tgz › 71cc89d2-67b2-45e2-b6e9-9bac332a7aab.pdf]

STATUS PAGE

PROTOCOL **06-155**

Closed To New Accrual

Closure Effective Date: 06/16/09

No new subjects may be enrolled in the study as described above. Any questions regarding this closure should be directed to the study's Principal Investigator

DANA-FARBER CANCER INSTITUTE
DANA-FARBER / HARVARD CANCER CENTER

Office for Human Research Studies

Protocol Front Sheet

DFCI Protocol No.: 06155

1. PROTOCOL TITLE AND VERSION

Title: A Phase 1/2 Study of the Safety of Opebacan in Patients Undergoing Myeloablative HSCT
Protocol Version No./ Date: 6/4/15
Sponsor Study Number: N/A

2. DF/HCC STUDY CONTACT INFORMATION

Primary Study Contact: Lisa Brennan RN **Email:** lisa_brennan@dfci.harvard.edu **Phone:** 632-3846

INVESTIGATORS: (List only those under DFCI IRB, *i.e.*, from institutions listed in Section 6 below)

**Overall PI:**	Eva C. Guinan MD	**Phone:** 632-4932	**Institution(s):** DFCI
**Site Responsible PI:**	Robert Soiffer MD	**Phone:** 632-4731	**Institution(s):** DFCI
	David Avigan MD		
	617-667-9920		

3. DRUG / DEVICE INFORMATION N/A:

☒ **Drug(s), Biologic(s)**: Opebacan
Provided by: Xoma
IND Exempt: ☐ -or-
IND#: 100082 **Holder Type:** Industry
IND Holder Name: IND is now closed

☐ **Device(s) Name**:
Provided by:
IDE Exempt: ☐ -or-
IDE #: **Holder Type:** [pull down]
IDE Holder Name:

4. PROTOCOL COORDINATION, FUNDING, PHASE, MODE, TYPE ETC.

Regulatory Sponsor:
Industry: regulatory sponsor is no longer XOMA

Cancer Related: [pull down] If yes:
Primary Disease Program:
[pull down]
or
Primary Discipline Based Program:
Transplant

Funding/Support (check all that apply)**:**
☐ Industry:
☐ Federal Organization:
Grant #:
☐ Internal Funding:
☐ Non-Federal:
☒ Other: Funding has stopped

CTEP Study: No

Phase: Phase 1/2

Multi-Center (i.e., non-DF/HCC site participation)**:**
Yes

Protocol Type: IAA - Therapeutic
If Ancillary, provide parent protocol #:

Protocol Involves (check all that apply as listed in the protocol document, even if not part of the research but is mandated by the protocol document)**:**

☐ Chemotherapy	☐ Hormone Therapy	☒ Medical Record Review
☐ Immunotherapy	☐ Vaccine	☐ Questionnaires/Surveys/Interviews
☐ Surgery	☐ Data Repository	☒ Radiological Exams
☒ Bone Marrow/Stem Cell Transplant	☐ Exercise/Physical Therapy	☐ Required Biopsy Study
☐ Cell Based Therapy	☐ Genetic Studies	☐ Human Embryonic Stem Cell
☐ Gene Transfer (use of recombinant DNA)	☐ Human Material Banking	☐ Quality of Life
☐ Radiation Therapy	☒ Human Material Collection	☐ Other:

5. SUBJECT POPULATION (also applies to medical record review and specimen collection studies)

Total Study-Wide Enrollment Goal: 30
Greater than 25% of the overall study accrual will be at DF/HCC: ☒ Yes ☐ No
Total DF/HCC Estimated Enrollment Goal: 20
Adult Age Range: greater than or equal to 17years **Pediatric Age Range:**
Will all subjects be recruited from pediatric clinics? ☐ Yes ☒ No
If enrolling both adults and pediatric subjects, anticipated percent of pediatric subjects:
Retrospective Medical Record Reviews only (Please provide date range): from to

6. DF/HCC PARTICIPANTS UNDER DFCI IRB (check all that apply)

☐ Beth Israel Deaconess Medical Center (BIDMC)
☒ Beth Israel Deaconess Medical Center – Needham (BIDMC-Needham)
☐ Boston Children's Hospital (BCH)
☒ Brigham and Women's Hospital (BWH)
☒ Dana-Farber Cancer Institute (DFCI)
☐ Dana-Farber/New Hampshire Oncology-Hematology (DFCI @ NHOH)
☐ DF/BWCC in Clinical Affiliation with South Shore Hospital (DFCI @ SSH)
☐ Dana-Farber at Milford Regional Cancer Center (DFCI @ MRCC)
☐ Dana-Farber at Steward St. Elizabeth's Medical Center (DFCI @ SEMC)
☐ Massachusetts General Hospital (MGH)
☐ Mass General/North Shore Cancer Center (MGH @ NSCC)
☐ Mass General at Emerson Hospital – Bethke (MGH @ EH)
☐ New England Cancer Specialists (NECS)

7. NON-DF/HCC PARTICIPANTS UNDER DFCI IRB (check all that apply)

☐ Cape Cod Healthcare (CCH)
☐ Lowell General Hospital (LGH)
☐ New Hampshire Oncology-Hematology-P.A. (NHOH)
☐ Newton-Wellesley Hospital (NWH)
☐ Broad Institute
☐ Lawrence & Memorial Cancer Center in affiliation with Dana-Farber Community Cancer Care (LMCCC)

Protocol Front Sheet

8. DF/HCC INITIATED STUDIES ONLY - INSTITUTIONAL PARTICIPANTS UNDER OTHER IRB (N/A:)

DF/HCC Multi-Center Protocols: (list institution/location)

DF/PCC Network Affiliates: (list institution/location)

Protocol Number: 06-155

Approval Date: 08/22/06 (IRB meeting date when protocol/consent approved or conditionally approved)

Activation Date: 01/26/07 (Date when protocol open to patient entry)

Approval signatures are on file in the Office for Human Research Studies, tel. 617-632-3029.

Date Posted	Revised Sections	IRB Approval Date	OHRS Version Date
04/02/07	Front Sheet Replaced Due To Amendment # 1	03/28/07	-
6/15/07	Consent Forms (2), Protocol and Front Sheet replaced due to Amendment # 2	6/5/07	-
07/05/07	Delayed Activation Alert Page replaced: MGH ready for activation (Note: previously activated at BIDMC on 01/26/07; DFCI and TCH's will join at later date)	N/A	-
07/11/07	Correction: Consent Forms replaced due to incorrect versions provided previously	N/a	-
08/21/07	Consent Form replaced (footer) due to Continuing Review #1	08/21/07	-
10/17/07	Protocol and Front sheet replaced due to Amendment #4	10/02/07	-
12/17/07	Front Sheet replaced due Amendment # 3	12/12/07	-
02/27/08	Main Consent Form, Alert Page, Protocol and Front Sheet replaced added due to Amendment #7	02/26/08	-
02/28/08	Delayed Activation Alert Page removed: BWH ready for activation) Note: previously activated at BIDMC on 01/26/07 and at MGH on 07/05/07; DFCI and TCH's will no longer join); Donor Consent Form replaced due to Amendment # 5	11/29/07	-
03/17/08	Front Sheet replaced due Amendment # 8	03/14/08	-
06/12/08	Front Sheet replaced due Amendment # 6	06/12/08	-
08/15/08	Consent Forms replaced due to Continuing Review #2	08/07/08	-
07/28/09	Closure to accrual (Effective 06/16/09) and Study renewal due to Continuing Review #3	07/14/09	-
07/12/10	Study renewal/Consent Forms (footers) replaced due to Continuing Review #4	07/07/10	-
11/01/10	BIDMC site ON HOLD due BIDMC Strategic Hold OE #5	N/A	-
01/12/11	Request to remove Administrative Hold BIDMC due to Amendment # 9 (OE # 5; letter date 12/30/10)	01/11/11	-
06/28/11	Study renewal and Front Sheet replaced due to Continuing Review #5	06/19/11	-
09/22/11	Front Sheet replaced due Amendment #10	09/14/11	-
05/31/12	Study renewal/Consent Forms (footers) replaced due to Continuing Review #6	05/28/12	-
04/10/13	Study renewal/Consent Form footers replaced due to Continuing Review #7	04/10/13	N/A
02/13/14	Study renewal/Consent Form footers replaced due to Continuing Review #8	02/12/14	n/a
12/16/14	Study renewal/Consent Form footers replaced due to Continuing Review #9	12/15/14	N/A

Date Posted	Revised Sections	IRB Approval Date	OHRS Version Date
07/21/15	Protocol and Front Sheet replaced due to Amendment #11	07/16/15	N/A

XOMA (US) LLC

**Title:**	A Phase I/II Study of the Safety and Pharmacokinetics of Opebacan ($rBPI_{21}$) in Patients Undergoing Myeloablative Allogeneic Hematopoietic Stem Cell Transplantation (HSCT)
**Protocol Number:**	BPSC030 Amendment 5 (BPSC030.05)
**Study Drug:**	Opebacan
**IND:**	100082
**Medical Monitor:**	Alan M. Solinger, M.D.<br>(510) 204-7448
**Sponsor:**	XOMA (US) LLC<br>2910 Seventh Street, Berkeley, CA 94710
**Original Protocol Date:**	March 1, 2006
**Amendment 1 Date:**	September 27, 2006
**Amendment 2 Date:**	November 22, 2006
**Amendment 3 Date:**	January 4, 2007
**Amendment 4 Date:**	July 27, 2007
**Amendment 5 Date:**	February 6, 2008

Amendment 6 made by Research team: 6/4/2015

CONFIDENTIAL

This is a XOMA (US) LLC document that contains confidential information. It is intended solely for the recipient clinical investigator(s) and must not be disclosed to any other party. The material may be used only for evaluating or conducting clinical investigations; any other proposed use requires written consent from XOMA (US) LLC.

SYNOPSIS

<table>
<tr><td>Protocol Title:</td><td>A Phase I/II Study of the Safety and Pharmacokinetics of Opebacan ($rBPI_{21}$) in Patients Undergoing Myeloablative Allogeneic Hematopoietic Stem Cell Transplantation (HSCT)</td></tr>
<tr><td>Protocol Number:</td><td>BPSC030 Amendment 5 (BPSC030.05)</td></tr>
<tr><td>Sponsor:</td><td>XOMA (US) LLC</td></tr>
<tr><td>Study Drug:</td><td>Opebacan</td></tr>
<tr><td>Objectives:</td><td>1. To demonstrate the safety of escalating doses of opebacan in subjects undergoing myeloablative allogeneic HSCT
2. To determine the pharmacokinetics of opebacan in subjects undergoing myeloablative allogeneic HSCT
3. To determine if IV administration of opebacan is associated with changes in biological markers for inflammation
4. To develop preliminary descriptive data on the occurrence and severity of HSCT-related complications, including acute graft vs. host disease (aGvHD)</td></tr>
<tr><td>Study Design:</td><td>This is a phase I/II, open-label, dose-escalation study of the safety and pharmacokinetics (PK) of opebacan in subjects undergoing non-T cell depleted myeloablative HSCT from an allogeneic donor. Subjects who sign an informed consent and pass initial screening prior to the start of their HSCT conditioning regimen will be re-evaluated on Day −1. Subjects who meet the eligibility criteria for HSCT at final screening will begin their IV infusions of opebacan on Day −1. Subjects whose conditioning regimen consists of chemotherapy only will receive a continuous opebacan infusion following completion of chemotherapy treatment on Day −1. Subjects whose conditioning regimen includes total body irradiation (TBI) will have three 1-hour interruptions in their opebacan infusions (one on Day −1 and two on Day 0) to accommodate TBI treatment. Following the final interruption on Day 0, the infusion must be restarted at least 2 hours prior to the HSCT.

The HSCT procedure will take place on Day 0, between 18 and 36 hours following the start of opebacan treatment, and approximately 4 to 12 days following initiation of the conditioning regimen.

Eligible subjects will be assigned to one of five successive dose groups of six subjects each. Subjects in successive dose groups will be treated with opebacan at escalating doses and durations as shown in the table below.
<table>
<tr><th>Dose Group</th><th>No. of Subjects</th><th>Dose Regimen</th></tr>
<tr><td>1</td><td>6</td><td>4 mg/kg continuous IV infusion for 30 minutes followed immediately by 6 mg/kg/day continuous IV infusion for 3 days</td></tr>
<tr><td>2</td><td>6</td><td>4 mg/kg continuous IV infusion for 30 minutes followed immediately by 9 mg/kg/day continuous IV infusion for 3 days</td></tr>
<tr><td>3</td><td>6</td><td>4 mg/kg continuous IV infusion for 30 minutes followed immediately by 12 mg/kg/day continuous IV infusion for 3 days</td></tr>
<tr><td>4</td><td>6</td><td>4 mg/kg continuous IV infusion for 30 minutes followed immediately by 12 mg/kg/day continuous IV infusion for 7 days</td></tr>
<tr><td>5</td><td>6</td><td>4 mg/kg continuous IV infusion for 30 minutes followed immediately by 12 mg/kg/day continuous IV infusion for 14 days</td></tr>
</table>
After all subjects in a dose group have completed study procedures through Day 28, all available safety information will be reviewed by the Data Safety Monitoring Board (DSMB). Dosing may begin for the next dose group at the next higher dose level if no more than one subject in the previous group has experienced a dose-limiting toxicity (DLT) and, in the opinion of the DSMB, there are no clinically significant safety</td></tr>
</table>

SYNOPSIS *(continued)*

**Study Design *(continued):***	concerns at the previous dose level. All subjects will have disease status, performance score, and cumulative aGvHD status confirmed on Day 100 (± 14 days) by clinic visit, if possible, or by phone.<br>Safety will be assessed by pre- and post-treatment serial measurements of vital signs and clinical laboratory assessments and by the recording of adverse clinical events, with an emphasis on infectious and non-infectious regimen-related toxicities. PK data will be collected and analyzed to determine dose/duration relationships and to explore preliminary correlations of PK data with demographic information, clinical course through transplant, occurrence of transplant regimen-related toxicities including aGvHD, and laboratory markers of inflammation.<br>If a subject in any cohort experiences graft failure, the subject's cohort will be expanded to 12 subjects. If a second graft failure occurs in the expanded cohort, the DSMB will determine whether the study should be stopped after reviewing all available information on the two subjects whose grafts failed.
**Hypothesis:**	IV infusion of opebacan to replace endogenous BPI during the peritransplant period will result in the reduction of LPS-induced inflammatory sequelae, in particular aGvHD, in patients undergoing allogeneic HSCT.
**Study Population:**	Patients undergoing non-T-cell-depleted myeloablative allogeneic hematopoietic stem cell transplantation (HSCT)
**Sample Size:**	Up to 60; the expected enrollment is approximately 30 – 40 subjects.
**No. of Sites:**	3
**Study Duration for Subjects**	The duration of subject participation is approximately 105 to 150 days (from prescreening to the Day 100 follow-up assessment)
**Outcome Measures**	**Biological and Clinical Activity:**<br>Biological and clinical activity will be assessed through an evaluation of the following outcome measures:<br>• Time to engraftment, defined as the first of three consecutive measurements of ANC ≥ 500/µL<br>• Inflammatory markers (such as LPS, LBP, IL-6, CRP and other exploratory biomarkers)<br>• Inflammatory states such as presence of fever and infection<br>• Transplant-related complications including, but not limited to, aGvHD, inadequate oral intake, presence of oral mucositis, veno-occlusive disease of the liver, and idiopathic pulmonary fibrosis<br>**Safety:**<br>Safety will be assessed by pre- and post-treatment serial measurements of vital signs and clinical laboratory assessments and by the recording of adverse clinical events, with an emphasis on infectious and non-infectious regimen-related toxicities.<br>**Immunology:**<br>All subjects must have samples for anti-opebacan antibody analysis drawn at prescreening. Samples will also be collected at Day 28 to detect whether subjects have developed anti-opebacan antibodies.<br>**Pharmacokinetics:**<br>The pharmacokinetics of opebacan will be determined through the collection of PK samples from each subject throughout the study.

SYNOPSIS *(continued)*

**Estimated Start:**	Q4 2006
**Estimated Finish:**	Q2 2009
**Safety:**	All regulations stated in 21 CFR Parts 50, 56 and 312 and recommendations outlined in the ICH Guidelines for Good Clinical Practice will be adhered to throughout this trial. The safety of the drug will be assessed by multiple subject assessments of vital signs, physical exams, clinical tests and laboratory evaluations. Concomitant medications and adverse events will be monitored and tracked.
**Statistical Considerations:**	Data will be analyzed for safety, pharmacokinetics and biological endpoints. Safety analyses will include all subjects who received any amount of study drug. Analyses of the population of subjects who completed treatment may also be performed. Since the sample size is small, no formal statistical testing will be performed. Study results will be summarized by descriptive statistics.<br>The sample size for this study is based primarily on PK/PD considerations. The planned accrual is six subjects in each of the five successive dose groups. Formal sample size calculations were not done; past experience with this type of study suggests that this number of subjects will be adequate to characterize the PK/PD profile at each dose level.

TABLE OF CONTENTS

SYNOPSIS ii

TABLE OF CONTENTS v

LIST OF TABLES viii

LIST OF FIGURES viii

LIST OF APPENDICES viii

LIST OF ABBREVIATIONS ix

1.0 BACKGROUND 1

- 1.1 Overview 1
- 1.2 Innate Immune System 1
- 1.3 BPI and Opebacan ($rBPI_{21}$) 2
- 1.4 Prior Human SCT Data 4
- 1.5 Study Rationale 4

2.0 OBJECTIVES 5

3.0 STUDY DESIGN 6

- 3.1 Description of the Study 6
- 3.2 Rationale For Study Design and Dose Level 9
  - 3.2.1 Rationale for Dose Level 9
    - 3.2.1.1 Determining Therapeutic Levels of an Anti-endotoxin Therapeutic 9
    - 3.2.1.2 An Endotoxin Antagonist in an aGvHD Animal Model: Clinical Study Considerations 10
    - 3.2.1.3 Endogenous BPI and LPS Levels Following Hematopoietic Stem Cell Transplant 10
    - 3.2.1.4 Nonclinical Safety Assessment Summary 11
  - 3.2.2 Rationale for Study Design 13
- 3.3 Selection of Doses Used in the Study 14
  - 3.3.1 Dosing Groups 14
  - 3.3.2 Definition and Management of Dose-limiting Toxicity 14
  - 3.3.3 Definition of Maximum Tolerated Dose 15
  - 3.3.4 Dose Escalation Plan 15
- 3.4 Selection of Study Population 18
  - 3.4.1 Prescreen Eligibility Criteria 18
    - 3.4.1.1 Prescreen Inclusion Criteria 18
    - 3.4.1.2 Prescreen Exclusion Criteria 19
  - 3.4.2 Final Pretreatment Eligibility Criteria 20
    - 3.4.2.1 Final Pretreatment Inclusion Criteria 20
    - 3.4.2.2 Final Pretreatment Exclusion Criteria 21
- 3.5 Enrollment 21

3.6 Treatment 21
3.6.1 Formulation 21
3.6.2 Labeling....22
3.6.3 Shipping 22
3.6.4 Warnings 22
3.6.5 Dosage, Administration, and Storage....23
3.6.5.1 Dosage....23
3.6.5.2 Administration 23
3.6.5.3 Storage and Handling....23
3.6.6 Prior and Concomitant Therapy 24
3.6.6.1 Excluded Concomitant Medications 24
3.6.6.2 Allowed Concomitant Medications 24
3.7 Supportive Care Guidelines....25
3.8 Outcome Measures 25
3.8.1 Biological and Clinical Activity....25
3.8.2 Safety Outcome Measures....26
3.8.3 Pharmacokinetic Assessment and Sampling Schedule....26
3.8.4 Immunogenicity Assessment....26
3.9 Assay Methods 27
3.9.1 Pharmacokinetics Assay....27
3.9.2 Immunological Assay....27
4.0 STUDY OPERATIONS AND EVALUATIONS....28
4.1 Overview 28
4.2 Reporting and Recording of Data....28
4.3 Study Assessments 29
4.3.1 Screening Assessments....30
4.3.2 Assessments for Treatment and Follow-up Period....32
4.3.2.1 Assessments for Treatment and Follow-up Period: Dose Groups 1, 2, and 3 32
4.3.2.2 Assessments for Treatment and Follow-up Period: Dose Group 4 48
4.3.2.3 Assessments for Treatment and Follow-up Period: Dose Group 5 65
4.4 Donor Assessments 81
4.5 Discontinuation 82
4.5.1 Subject Discontinuation 82
4.5.2 Study Discontinuation....82
5.0 ASSESSMENT OF SAFETY 83
5.1 Adverse Events....83
5.2 Baseline Medical Conditions and Underlying Disease 85
5.3 Laboratory Tests/Other Evaluations....85

5.4 Procedures for Eliciting, Recording, and Reporting Adverse Events 86
5.4.1 Recording Adverse Events 86
5.4.2 Adverse Events Requiring Expedited Reporting....87
5.4.3 Special Reporting Situations 87
5.4.3.1 Death 87
5.4.3.2 Hospitalizations for Surgical or Diagnostic Procedures 88
5.5 Type and Duration of Follow-up After Adverse Events 88
5.5.1 Post Treatment Follow-up....88
5.5.2 Post Adverse Event Follow-up....88
6.0 DATA ANALYSIS AND STATISTICAL METHODS....89
6.1 Disposition of the Study Subjects and Conduct of the Study....89
6.2 Determination of Sample Size....89
6.3 Analysis of Treatment Group Comparability 89
6.4 Biological and Clinical Activity Analysis....89
6.5 Safety Analysis....89
6.5.1 Adverse Events....91
6.5.2 Clinical Laboratory Evaluation 91
6.5.3 Vital Signs 91
6.5.4 Antibody Response to Opebacan 92
6.5.5 Concomitant Medication 92
6.6 Missing Data 92
6.7 Pharmacokinetic, Pharmacodynamic, and Other Analyses 92
6.8 Interim Analyses....92
7.0 INVESTIGATOR REQUIREMENTS AND QUALITY CONTROL....93
7.1 Study Initiation....93
7.2 Study Completion....93
7.3 Informed Consent....94
7.4 Institutional Review Board Approval....94
7.5 Ethical Considerations....94
7.6 Study Monitoring Requirements 95
7.7 Case Report Forms 95
7.8 Study Medication Accountability....95
7.9 Disclosure of Data 95
7.10 Retention of Records 96
7.11 Quality Assurance 96
8.0 REFERENCES....97

LIST OF TABLES

Table 1 Study BPSC030 Dose Groups....6
Table 2 Probabilities of Dose Escalating Assuming Different DLT Incidence Rates....18
Table 3 Required Label Information....22

LIST OF FIGURES

Figure 1 Subject Enrollment and Treatment Timeline....8
Figure 2 Plasma Concentrations in Patients Undergoing HSCT....11
Figure 3 Plasma Concentration-time of Opebacan for Dosing Regimens in HSCT Subjects....12
Figure 4 Dose Escalation Diagram....17

LIST OF APPENDICES

Appendix 1 Study BPSC030 Schedule of Assessments....1
Appendix 2 Study BPSC030 PK Sampling Schedule....4
Appendix 3 Karnofsky Performance Criteria....5
Appendix 4 Consensus Grading for Acute GvHD....6
Appendix 5 Oral Mucositis Assessment....7

LIST OF ABBREVIATIONS

AE	Adverse event
aGvHD	Acute graft-versus-host disease
ALT	Alanine aminotransferase
ANC	Absolute neutrophil count
ARDS	Adult respiratory distress syndrome
AST	Aspartate aminotransferase
BUN	Blood urea nitrogen
CBC	Complete blood count
CHF	Congestive heart failure
CFR	Code of Federal Regulations
CMV	Cytomegalovirus
CPK-MB	Creatine phosphokinase-MB band
CRA	Clinical Research Associate
CRF	Case Report Form
CTC	Common Toxicity Criteria
DLT	Dose-limiting toxicity
DSMB	Data Safety Monitoring Board
ECHO	Echocardiography
EOI	End of infusion
FDA	Food and Drug Administration
FDP	Fibrin degradation product
FSP	Fibrin split products
GCP	Good Clinical Practice
GFR	Glomerular filtration rate
GI	Gastrointestinal
GvHD	Graft-versus-host disease
GvL	Graft versus leukemia
HDL	High density lipoprotein
HIV	Human immunodeficiency virus (antibody)
HTLV	Human T-lymphotropic virus (antibody)
HSCT	Hematopoietic stem cell transplantation
I&O	Intake and output
ICH	International Conference on Harmonization

LIST OF ABBREVIATIONS *(continued)*

IND	Investigational New Drug application
IRB	Institutional Review Board
IV	Intravenous
IVIG	Intravenous immunoglobulin
LAL	Limulus amoebocyte lysate
LBP	Lipopolysaccharide-binding protein
LDH	Lactate dehydrogenase
LPS	Lipopolysaccharide
LVEF	Left ventricular ejection fraction
MTD	Maximum tolerated dose
MUGA	Multiple gated acquisition
NOAEL	No-observed-adverse-effect-level
NOEL	No-observed-effect-level
PCP	*Pneumocystis carinii* pneumonia
PCR	Polymerase chain reaction
PK	Pharmacokinetic
PO	By mouth (per oral)
pO_2	Partial pressure O_2
PT	Prothrombin time
PTT	Partial thromboplastin time
$rBPI_{21}$	A second generation recombinant N-terminal BPI protein; a derivative of $rBPI_{23}$ in which one of three cysteines (Cys132) has been replaced with an alanine. In this protocol $rBPI_{21}$ is referred to as opebacan, its generic designation.
$rBPI_{23}$	A recombinant 23 kDa N-terminal BPI protein closely related to $rBPI_{21}$
SAE	Serious adverse event
SNPs	Single nucleotide polymorphisms
TBI	Total body irradiation
TNF	Tumor necrosis factor
TLR	Toll-like receptor
TPN	Total parenteral nutrition
USP	United States Pharmacopeia
VD	Volume of distribution
VOD	Veno-occlusive disease
WBC	White blood cell (count)

1.0 BACKGROUND

1.1 Overview

The outcome of allogeneic stem cell transplantation remains limited by significant regimen related toxicities, including infection, end organ damage, and acute graft-versus-host disease (aGvHD). These morbidities have in common a pronounced inflammatory state in the host. A growing body of scientific evidence indicates that aGvHD is triggered in part by gram-negative bacterial lipopolysaccharide (LPS or endotoxin) that induces monocytes to produce pro-inflammatory cytokines that activate donor T cells (Cooke et al., 1998). Entry of LPS into the systemic circulation, either via translocation across the damaged gastrointestinal mucosa and/or as a result of invasive infection with gram-negative bacteria, has been demonstrated during myeloablative HSCT both in animal models (Cooke et al., 2002) and humans (Levy et al., 2003). LPS-induced production of pro-inflammatory and Th1-polarizing cytokines such as TNFα has been clearly linked to consequent aGvHD (Cooke et al., 1998; Cooke et al., 2000; Cooke et al., 2002). In addition, LPS has been shown to be an important modulator of endothelial cell function with effects likely to result in increased recruitment of inflammatory cells to sites of LPS-mediated endothelial activation. Inflammatory or infectious states, as assessed by surrogate markers such as C reactive protein elevation or by direct evidence of active infection, are associated with multiple poor outcomes after transplant, including poor nutritional status, increased rates of aGvHD, increased 100-day mortality, and increased all-cause transplant mortality (Holler, 1995).

Importantly, LPS antagonism has shown efficacy in reducing mortality and aGvHD in murine myeloablative HSCT models (Cooke et al., 2001; Cooke et al., 2002). Using a synthetic lipid A analog to antagonize LPS, Cooke et al. demonstrated that administration of the lipid A antagonist early in the time course (Days 0 to 6) of allogeneic SCT in mice caused significant reductions in TNFα and intestinal damage as well as significant survival advantage compared to controls (Cooke et al., 2002). Blockade of LPS in this study reduced the severity of aGvHD while preserving the graft vs. leukemia (GvL) effects associated with SCT (Cooke et al., 2002).

Opebacan ($rBPI_{21}$) is a recombinant N-terminal fragment of the human bactericidal/ permeability-increasing protein (BPI), a neutrophil-derived anti-infective protein with potent endotoxin-neutralizing properties. This study will evaluate the safety and pharmacokinetics of opebacan as a first step in assessing the potential of this agent in reducing peritransplant morbidities, including aGvHD, in patients undergoing HSCT.

1.2 Innate Immune System

Over the past two decades, major advances have been made in understanding the molecular basis of the innate immune system in host defense (Hoffman, 1999) and its role in human health and disease (Abreu and Arditi, 2004; Orange et al., 2005). The innate immune system is the first response to infection and directs the subsequent adaptive immune response (Janeway and Medzhitov, 2002). The presence of microbes in normally sterile body sites is

detected by a family of innate immune receptors known as toll-like receptors (TLRs) (Akira and Takeda, 2004). Perhaps the best studied TLR agonist is LPS, a molecule that is uniquely expressed on the outer leaflet of the outer membrane of gram-negative bacteria (Ulevitch and Tobias, 1999). Upon entry into the systemic circulation, LPS is recognized by the liver-derived LPS-binding protein (LBP), a ~50 kDa protein that serves to disaggregate LPS aggregates and deliver LPS monomers to the LPS receptor complex, comprised of TLR4 and the glycosylphosphoinositide-linked receptor membrane CD14 (mCD14), on host leukocytes. LBP therefore catalyzes the delivery of LPS to its receptor, resulting in remarkable LPS potency such that the presence of even picogram quantities of LPS in blood plasma can activate inflammatory responses of host leukocytes. Soluble CD14 (sCD14) plays important roles in delivering LPS to CD14 negative cells such as endothelial cells (Yu and Wright, 1996).

With growing appreciation of the sensitive detection mechanisms of mammalian innate defense has come a better understanding of how these inflammatory signals are down-regulated. One such mechanism is by the action of neutrophil-derived granule-associated antimicrobial proteins and peptides (Levy, 2004). These granule-associated cationic molecules are capable of binding microbial components and neutralizing their pro-inflammatory activity. Among the human neutrophil antimicrobial proteins and peptides is BPI, a cationic 55 kDa protein that possesses potent anti-endotoxic activity based upon its high (nanomolar) affinity for the lipid A region common to the LPS of all gram-negative bacteria (Gazzano-Santoro et al., 1992; Levy, 2000). Binding of BPI to LPS blocks interaction of LPS with LBP and LPS receptors and thereby inhibits all of the myriad LPS-induced inflammatory responses, including potent inhibition of LPS-induced production of TNFα (Weiss et al., 1992). Importantly, BPI has a higher affinity for LPS than LBP (Opal et al., 1994) and has superior potency in inhibiting the endotoxic activity of LPS in comparison to other antimicrobial peptides including defensins and cathelicidin peptides (Levy et al., 1995).

1.3 BPI and Opebacan ($rBPI_{21}$)

Recombinant N-terminal fragments of BPI (including $rBPI_{23}$ and opebacan [$rBPI_{21}$, NEUPREX®]) possessing the LPS-binding and LPS-neutralizing activity of holo-BPI have been developed as potential anti-infective agents by XOMA (US) LLC (Berkeley, CA). These BPI congeners possess potent *in vitro* endotoxin-neutralizing activity towards LPS in complex biologic fluids including plasma, serum and whole human blood (Weiss et al., 1992). N-terminal rBPI fragments have also demonstrated efficacy in multiple models of endotoxemia of rodents, rabbits, and non-human primates (Rogy et al., 1994; Lin et al., 1994a; Jin et al., 1995; Dahlberg et al., 1996; Lin et al., 1996).

The feasibility and safety of delivering rBPI to humans has been demonstrated in phase I trials where it has been well-tolerated, safe, and non-immunogenic (Wiezer et al., 1998). Intravenous infusion of $rBPI_{23}$ was able to blunt LPS-induced fever, changes in cardiac index, cytokine release (including TNFα) and activation of clotting cascades in human volunteers (von der Mohlen et al., 1995; von der Mohlen, 1995a). A number of phase II

studies have been directed at conditions in which gram-negative bacteremia and/or endotoxemia were believed to play a role in disease. A double-blind, placebo-controlled phase II trial in patients undergoing partial hepatectomy demonstrated that administration of opebacan was associated with reduced leukocyte activation (Wiezer et al., 2000) and prevention of plasma amino acid abnormalities (Nijveldt et al., 2001). Opebacan treatment was also associated with reduced incidence of pneumonia and adult respiratory distress syndrome (ARDS) in patients with hemorrhagic trauma (Demetriades et al., 1999). An open-label phase II trial of opebacan in 26 patients with fulminant meningococcemia suggested a beneficial effect on mortality; mortality was predicted to be ~20-40% (based on historic controls, clinical prognostic scores, and plasma cytokine levels) but in patients treated with opebacan was only ~4%. Based upon these promising results, a randomized, double-blind, placebo-controlled international phase III trial of opebacan for severe meningococcal sepsis was undertaken. The results of the trial showed that those treated with opebacan had a significantly higher frequency of functional outcome similar to that before illness, as measured by the Pediatric Overall Performance Category (POPC) scale at Day 60 after illness (77.3 vs. 66.3%, p = 0.019) (Levin et al., 2000). Moreover, there was a meaningful trend towards a reduction in multiple severe limb amputations in the opebacan treatment group (7.4% placebo, 3.2% opebacan, p = 0.067) and in multiple outcome measures including need for intubation, length of ICU stay, need for blood-product replacement and need for renal replacement therapies.

In keeping with its favorable safety profile, opebacan has no known toxic effects on the marrow or hematopoietic precursors and has in fact been associated with favorable effects on the marrow compartment. For example, $rBPI_{23}$ improved neutrophil recovery in a rat model of neutropenic sepsis. In critically ill children with meningococcal sepsis, there were significant differences in favor of opebacan vs. placebo in the number of cryoprecipitate transfusions received ($p = 0.002$), the proportion of patients receiving them ($p = 0.006$), and the total volume received ($p = 0.02$). Similarly, the number of transfusions and the proportion of patients receiving platelets were significantly lower in the opebacan group ($p = 0.02$ and $p = 0.03$, respectively). For other blood products and colloids, the results were consistently lower in the opebacan group (Levin et al., 2000). Lechner et al. demonstrated in rats severely immunosuppressed with cyclophosphamide that $rBPI_{23}$ infusion can significantly decrease mortality, compared to controls, when the animals are subsequently challenged with intravenous bacteria (Lechner et al., 1995). The study also indicated improvements in a number of supportive parameters (e.g., reduction in bacteria counts and hypothermia).

In summary, clinical trials of opebacan to date have demonstrated that it is well-tolerated, safe, and relatively non-immunogenic, and that it has endotoxin-neutralizing activity in humans. Opebacan is most likely to show a beneficial effect when given early in the course of endotoxemia (Giroir, 2001) and under conditions where the ratio of LPS to BPI (neutrophils) is high, i.e., conditions where there are high LPS concentrations in the face of inadequate endogenous BPI concentrations (Levy, 2002). These conditions are prominent features of patients undergoing myeloablative HSCT in that LPS translocation into the peripheral circulation has been demonstrated in animal (Cooke et al., 1998; Cooke et al.,

2002) and human (Levy et al., 2003) studies and endogenous BPI levels are low due to neutropenia (Levy et al., 2002).

1.4 Prior Human SCT Data

Levy et al. have previously shown that, while neutrophils derived from engrafted bone marrow contain normal quantities of BPI per cell 20 days following HSCT, the absolute neutrophil count (ANC) is near zero in the first weeks immediately after HSCT and thus the absolute quantity (i.e., total body content) of BPI will be markedly diminished during that period (Levy et al., 2002). More recently, Levy et al. have also demonstrated endotoxemia (elevated LPS) in a high proportion of patients early in the course of HSCT (including the day of the HSCT) and nearly universal elevation of LBP by 7 days following HSCT (Levy et al., 2003).

1.5 Study Rationale

The rationale for using opebacan in patients undergoing myeloablative regimens and HSCT is based on the following:

1. Endotoxemia has been demonstrated to play a central pathophysiologic role as a trigger of aGvHD in animal models.
2. Endotoxemia following HSCT is associated with inflammatory conditions (such as inflammatory cytokine release) that have been demonstrated in humans to be associated with organ damage and increased morbidity and mortality.
3. Endotoxemia and LBP elevation have been demonstrated in humans undergoing ablative HSCT.
3. Chemotherapy-induced neutropenia results in a deficiency of endogenous BPI levels.
4. The timing of the endotoxemic insult is predictable (i.e., subsequent to myeloablative chemotherapy and radiotherapy).
5. The return to normal neutrophil levels is not immediate and takes one week to several weeks.
6. Well established laboratory techniques for surrogate markers related to LPS presence and its activities can facilitate the evaluation of molecules designed to inhibit or antagonize LPS and its effects.

2.0 OBJECTIVES

The objectives of this study are as follows:

1. To demonstrate the safety of escalating doses of opebacan in subjects undergoing myeloablative allogeneic HSCT
2. To determine the pharmacokinetics of opebacan in subjects undergoing myeloablative allogeneic HSCT
3. To determine if IV administration of opebacan is associated with changes in biological markers for inflammation
4. To develop preliminary descriptive data on the occurrence and severity of HSCT-related complications, including aGvHD

3.0 STUDY DESIGN

3.1 Description of the Study

This is a phase I/II, open-label, dose- and duration-escalation study of the safety and PK of opebacan in subjects undergoing non-T cell depleted myeloablative HSCT from an allogeneic donor. Subjects who sign an informed consent and pass initial screening prior to the start of their HSCT conditioning regimen will be re-evaluated on Day −1. Subjects who meet the eligibility criteria for HSCT at final screening will begin their IV infusions of opebacan on Day −1. Subjects whose conditioning regimen consists of chemotherapy only will receive a continuous opebacan infusion following completion of chemotherapy treatment on Day −1. Subjects whose conditioning regimen includes total body irradiation (TBI) will have three 1-hour interruptions in their opebacan infusions (one on Day −1 and two on Day 0) to accommodate TBI treatment. Following the final interruption on Day 0, the infusion must be restarted at least 2 hours prior to the HSCT.

The HSCT procedure will take place on Day 0, between 18 and 36 hours following the start of opebacan treatment, and approximately 4 to 12 days following initiation of the conditioning regimen.

Eligible subjects will be assigned to one of five successive dose groups of six subjects each. Subjects in successive dose groups will be treated with opebacan at escalating doses and durations as shown in Table 1.

Table 1
Study BPSC030 Dose Groups

Dose Group	No. of Subjects	Dose Regimen	Total Dose
1	6	4 mg/kg continuous IV infusion for 30 minutes followed immediately by 6 mg/kg/day continuous IV infusion for 3 days	22 mg/kg
2	6	4 mg/kg continuous IV infusion for 30 minutes followed immediately by 9 mg/kg/day continuous IV infusion for 3 days	31 mg/kg
3	6	4 mg/kg continuous IV infusion for 30 minutes followed immediately by 12 mg/kg/day continuous IV infusion for 3 days	40 mg/kg
4	6	4 mg/kg continuous IV infusion for 30 minutes followed immediately by 12 mg/kg/day continuous IV infusion for 7 days	88 mg/kg
5	6	4 mg/kg continuous IV infusion for 30 minutes followed immediately by 12 mg/kg/day continuous IV infusion for 14 days	172 mg/kg

After all subjects in a dose group have completed study procedures through Day 28, all available safety information will be reviewed by the DSMB. Dosing may begin for the next dose group at the next higher dose level if no more than one subject in the previous group has experienced a DLT (see Section 3.3.2 for the definition of DLT) and, in the opinion of the DSMB, there are no clinically significant safety concerns at the previous dose level.

All subjects will have disease status, performance score, and cumulative aGvHD status confirmed on Day 100 (± 14 days) by clinic visit, if possible, or by phone. Safety will be assessed by pre- and post-treatment serial measurements of vital signs and clinical laboratory assessments and by the recording of adverse clinical events, with an emphasis on infectious and non-infectious regimen-related toxicities. PK data will be collected and analyzed to determine dose/duration relationships and to explore preliminary correlations of PK data with demographic information, clinical course through transplant, occurrence of transplant regimen related toxicities including aGvHD, and laboratory markers of inflammation. Figure 1 outlines the study design.

Figure 1
Subject Enrollment and Treatment Timeline

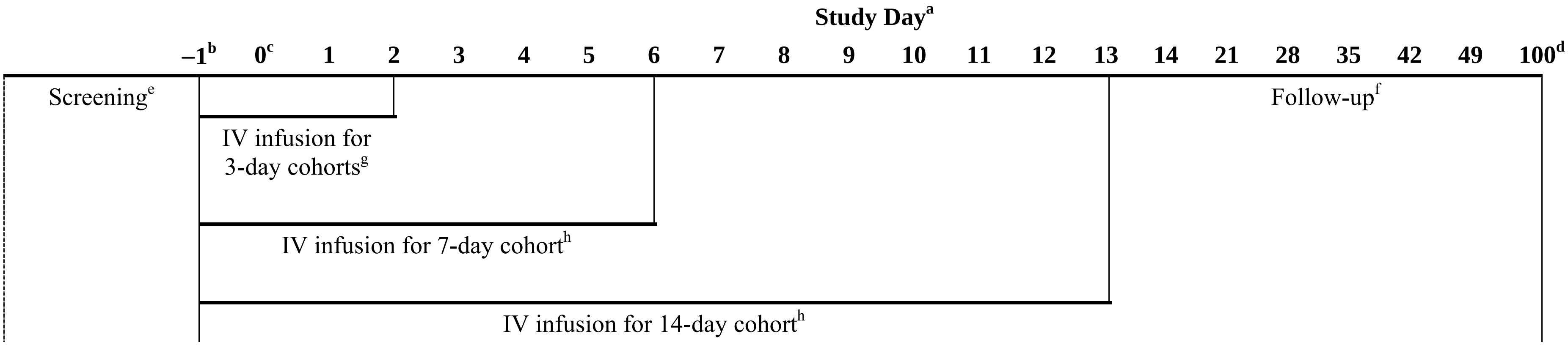

[a] The visit window for Days 21, 28, 42, and 49 is ± 3 days. The visit window for Day 100 is ± 14 days.

[b] IV infusion with opebacan will begin on Day –1, 18 to 36 hours prior to the HSCT. Prior to opebacan dosing, the Principal Investigator or designee must confirm and document that the subject meets all eligibility criteria for the HSCT and that the initiation of study drug administration will precede donor cell administration by 18 to 36 hours. Subjects whose conditioning regimen consists of chemotherapy only will receive a continuous opebacan infusion following completion of chemotherapy treatment on Day –1. Subjects whose conditioning regimen includes total body irradiation (TBI) will have three 1-hour interruptions in their opebacan infusions (one on Day –1 and two on Day 0) to accommodate TBI treatment. Following the final interruption on Day 0, the infusion must be restarted at least 2 hours prior to the HSCT.

[c] The HSCT will be performed on Day 0, 18 to 36 hours following initiation of opebacan treatment, and no less than 18 hours after the completion of chemotherapy treatment.

[d] All subjects will have disease status, performance score, and cumulative aGvHD status confirmed on Day 100 (± 14 days) by clinic visit, if possible, or by phone.

[e] Screening for eligibility will proceed in two parts. The first, a prescreen, will establish potential eligibility for treatment while a second, a final screen, will be used to determine whether drug administration will be initiated. Prescreening assessments must be performed within 30 days of admission to the hospital for the conditioning regimen and subsequent HSCT procedure. The final screen must occur on Day –1.

[f] All assessments scheduled on Days 14, 21, and 28 must be completed for all subjects whether or not the subjects have been discharged from the hospital. All assessments listed under "Discharge" in Appendix 1 must be completed at time of hospital discharge. Assessments scheduled for Days 35, 42, and 49 will be performed only on subjects who are still hospitalized. A final safety follow-up visit for all subjects will occur on Day 100.

[g] Subjects in the three 3-day cohorts will receive 6 mg/kg/day, 9 mg/kg/day, or 12 mg/kg/day for three days following an initial 4 mg/kg 30-minute infusion.

[h] Subjects in the 7- and 14-day cohorts will receive 12 mg/kg/day for 7 or 14 days, respectively, following an initial 4 mg/kg 30-minute infusion.

3.2 Rationale for Study Design and Dose Level

3.2.1 Rationale for Dose Level

3.2.1.1 Determining Therapeutic Levels of an Anti-endotoxin Therapeutic

It is hypothesized that continuous administration of opebacan therapy will provide continuous drug levels that will be available to neutralize circulating endotoxin and ameliorate endotoxin mediated activation of the innate immune system, thus preventing or reducing complications associated with endotoxemia, including aGvHD.

While absolute quantities of total endotoxin can be measured in the blood by limulus amoebocyte lysate (LAL) assays, quantitative measurements of the unbound fraction that is functional (i.e., inflammatory) are difficult to obtain because of the following:

1. The lipid A moiety of endotoxin can form micelles, as well as interact specifically with LBP and TLR4 and non-specifically with many biological molecules and structures (e.g., HDL, albumin and cell membranes). Each of these varied interactions affects endotoxin function, clearance, and area-under-the curve quantities.
2. Endotoxin typically enters the blood stream erratically, either from disruption of the gastrointestinal (GI) or urinary tract, or manifest clinically as bacteremia, and thus tends to result in erratic blood levels (and clinical manifestations) over time.
3. Circulating endotoxin is not one discrete molecule, but is always a complex mixture, with each bacterial serotype making its own unique combination of lipid A and carbohydrate backbone. Thus, endotoxin molecules have a wide range of affinities for specific receptors depending, in part, on the specific lipid A structure and the length of the carbohydrate backbone.

As a result, unbound endotoxin levels in the blood are almost impossible to reliably measure or model with any degree of clinical assurance. This lack of information makes the process of determining the target level of an endotoxin-neutralizing substance needed for a desired clinical effect very challenging.

For example, attempts at XOMA to model appropriate doses of opebacan to neutralize endotoxin based on *in vitro* and *in vivo* studies have resulted in estimated opebacan blood levels as high as 10 µg/mL—a level which, although safe when given in short infusions, is both clinically impractical and poses possible safety issues if given continuously over extended time periods. On the other hand, evidence of clinical activity, including significant and trending prospective measures of clinical benefit, were observed in a randomized, double-blinded trial in pediatric meningococcemia with 2 mg/kg opebacan administered continuously over 24 hours, yielding steady-state plasma levels of 67 ng/mL.

These mixed results suggest that establishing a dosing rationale for opebacan that seeks efficacy signals while maximizing safety will require the use of a composite of quantitative safety observations from animal toxicology studies, other animal data, and human experience and empirical observations in this population.

3.2.1.2 An Endotoxin Antagonist in an aGvHD Animal Model: Clinical Study Considerations

Cooke and colleagues evaluated an endotoxin antagonist (a synthetic lipid-A analogue) in a mouse aGvHD model (Cooke et al., 2001). Results from this study demonstrated that a course of endotoxin antagonist given for six days significantly reduced the later onset of aGvHD. In this animal model, the drug did not need to be present at the time when aGvHD was expected to occur, but rather before onset of aGvHD in a prophylactic mode. If opebacan prophylaxis proves useful in humans receiving stem cell transplants, the duration of such prophylaxis will need to be empirically determined since onset of aGvHD in humans starts between post-transplant Days 21 and 42, later than reported in the mouse model.

Prior to receiving the stem cell transplant, patients undergo an intensive conditioning regimen to destroy benign and, in some cases, malignant cells of the lymphoid and hematopoietic compartments. This regimen also kills other rapidly dividing normal cells, such as the epithelial cells lining the GI tract and their precursors. Data in mice indicate the damage to the GI epithelium results in leakage of bacterial endotoxin from the GI tract into the systemic circulation. This systemic endotoxin exposure has been linked to a concomitant inflammatory response that amplifies the donor anti-host T cell activities with resultant worsening of aGvHD (Cooke et al., 1998). Furthermore, when an endotoxin antagonist was administered from Day 0 through Day +6 after transplant, the severity of aGvHD was significantly reduced without evidence of any other interference with T cell function and the duration of leukemia-free survival was prolonged (Cooke et al., 2001). These results support the use of opebacan, an endotoxin antagonist, as a therapeutic agent to prevent aGvHD and suggest that this approach has potential to reach separate effects on GvHD from those on GvL.

3.2.1.3 Endogenous BPI and LPS Levels Following Hematopoietic Stem Cell Transplant

Levy, Guinan and colleagues, and XOMA are presently conducting a study to investigate the endogenous background levels of BPI and LPS in patients undergoing HSCT. Recent human data have shown that, post-conditioning, patient blood levels of endogenous BPI drop to undetectable levels rapidly as the ANC falls. During this period there is a concomitant rise in LBP (indicating endotoxemia); furthermore, as neutrophils recover in these patients, circulating levels of BPI rise and LBP diminish to normal pre-inflammatory levels (Levy, Guinan, et al., unpublished observations, Figure 2).

Figure 2
Plasma Concentrations in Patients Undergoing HSCT

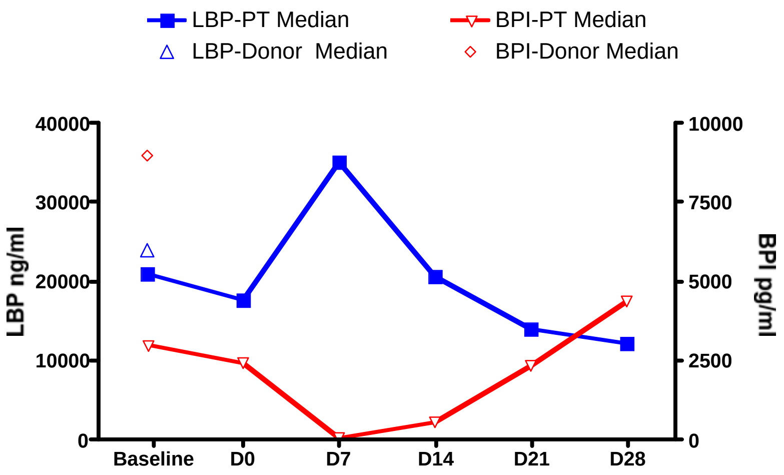

Based on all of the above, it is hypothesized that continuous administration of opebacan therapy to provide continuous drug levels, before infusion of donor cells, will make BPI available to neutralize circulating endotoxin and ameliorate endotoxin-mediated activation of the innate immune system, thus preventing or reducing complications associated with endotoxemia, including aGvHD.

3.2.1.4 Nonclinical Safety Assessment Summary

The general toxicity of opebacan was assessed in the rat and the monkey using the IV route of administration (bolus administration, continuous and intermittent infusion) for up to 28 days. Doses evaluated in these studies ranged from 2 to 200 mg/kg/day in the rat and from 10 to 200 mg/kg/day in the monkey.

Dose-related myocardial toxicity was observed in a single rat and a single monkey study following continuous infusion of formulated opebacan at ≥ 100 mg/kg/day for 3 to 5 days. No cardiotoxicity was seen in a number of other studies where formulated opebacan was continuously administered at doses up to 120 mg/kg/day in the rat and up to 75 mg/kg/day in the monkey for the same or longer duration, and where formulated opebacan was administered intermittently 4 times a day for 28 days at doses up to 150 mg/kg/day in the rat and up to 100 mg/kg/day in the monkey. The later data suggests that the duration of exposure may pose a greater risk of cardiac toxicity than the total daily dose.

Steady-state plasma opebacan levels were calculated for the rat and monkey studies where cardiotoxicity was evident. In the rat study, the steady-state plasma opebacan level corresponding to the cardiotoxic dose was approximately 3 μg/mL, and the no-observed-

adverse-effect-level (NOAEL) dose was approximately 1 µg/mL. These plasma values were determined using data from a rat pharmacokinetics study. Analysis of the data from the monkey study showed that the cardiotoxic dose for steady-state opebacan level in the monkey was 23 µg/mL. The steady-state plasma opebacan level corresponding to the NOAEL dose (10 µg/mL) was calculated using data from this study as well as from another study where 75 mg/kg/day was continuously infused for 28 days.

Based on these toxicology studies, a maximum desirable steady-state plasma concentration of 1 µg/mL was selected for this study. Using this steady-state level, estimates from human modeling of trauma subjects indicate that a dose of 24 mg/kg/day by continuous infusion should provide a blood level approaching 1 µg/mL (see Figure 3).

Figure 3
Plasma Concentration-time of Opebacan for Dosing Regimens in HSCT Subjects

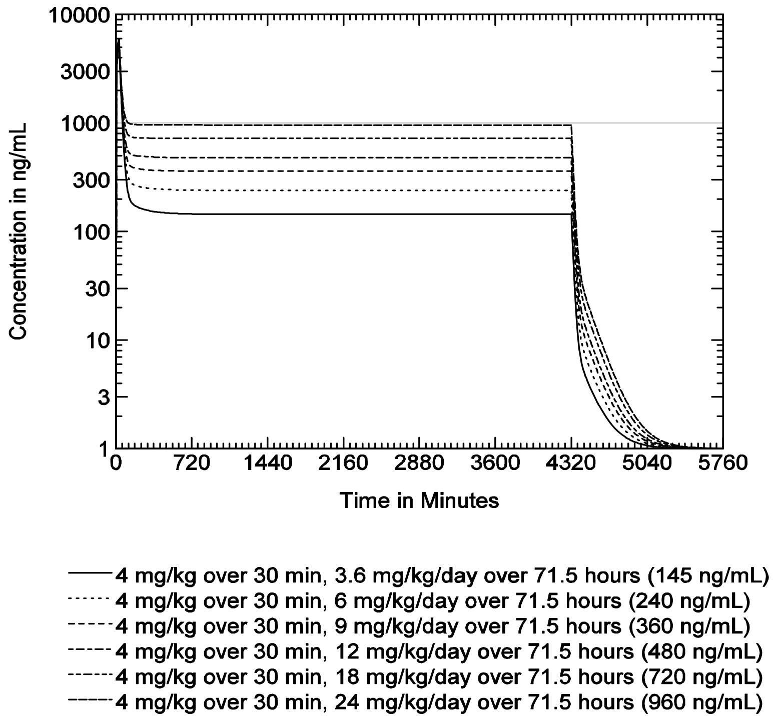

With this in mind, the rationale for the various components of the dosing regimens selected for this trial is as follows:

1. **Initial loading dose of 4 mg/kg × 30 minutes:**

   The initial dose of opebacan is meant to provide a transient high level of drug to initiate endotoxin neutralization and begin to replace missing natural BPI (decreased due to neutropenia from the conditioning regimen). This dose was selected because it gives transient high blood levels and is supported as safe by both animal toxicology data and human experience.

2. **Initial starting continuous dose of 6 mg/kg/day × 3 days:**

   The maximum dose of opebacan administered in humans in prior trials (hemorrhagic trauma) was 6 mg/kg/day × 3 days by continuous IV infusion, with no reported drug toxicities. The steady-state plasma levels following this regimen were about 250 – 280 ng/mL. According to XOMA's pharmocokinetic modeling, this dose is a quarter of the dose estimated to yield a steady-state plasma level of 1 µg/mL, the NOAEL in animal models.

3. **Escalation to 12 mg/kg/day × 3 days:**

   This dose is expected to give half the calculated dose capable of achieving the maximum desirable blood level (1 µg/mL).

4. **Escalation of infusion duration from 3 to 7 days (12 mg/kg/day × 7 days):**

   This infusion duration is approximately double the previous dose and half the proposed highest dose duration of 14 days. Neutropenia is expected to last well beyond 7 days.

5. **Escalation of infusion duration from 7 to 14 days (12 mg/kg/day × 14 days):**

   Continuous IV studies in rats and monkeys for 14 and 28 days have been completed. In rats, the NOAEL for cardiotoxicity was 50 mg/kg/day following continuous infusion for 14 days, with a steady-state plasma level of about 1 µg/mL. In monkeys, the NOAEL was 75 mg/kg/day for continuous infusion for 28 days, and yielding plasma levels about 10 µg/mL. Thus, these studies support 14-day continuous infusion in patients at doses that yield similar steady-state plasma levels. A dose of 12 mg/kg/day for 14 days is expected to yield plasma levels of about 0.5 µg/mL.

3.2.2 Rationale for Study Design

Opebacan has a well-established safety profile based on other trials but has never been administered to this patient population. Thus, a phase I/II open label, dose- and duration-escalation trial appears to be the appropriate trial design for ensuring an adequate safety margin while testing the hypothesis that opebacan may neutralize circulating endotoxin and thereby benefit stem cell transplant patients.

3.3 Selection of Doses Used in the Study

3.3.1 Dosing Groups

Eligible subjects in five successive dose groups of six subjects each will be treated with opebacan at the dose levels and dose durations shown in Table 1. All subjects will receive a 4 mg/kg continuous IV infusion for 30 minutes. Subjects enrolled in Dose Groups 1, 2, and 3 will then receive a continuous IV infusion at 6 (Group 1), 9 (Group 2) or 12 mg/kg/day (Group 3) for 3 days. For subjects in Dose Groups 4 and 5, the original rapid infusion will be followed, by a continuous IV infusion at 12 mg/kg/day for 7 (Group 4) or 14 (Group 5) days.

Subjects whose conditioning regimen includes total body irradiation (TBI) will have three 1-hour interruptions in their opebacan infusions (one on Day −1 and two on Day 0) to accommodate TBI treatment. Following the final interruption on Day 0, the infusion must be restarted at least 2 hours prior to the HSCT.

3.3.2 Definition and Management of Dose-limiting Toxicity

A DLT is defined as a toxicity of CTC grade 3 or greater severity (based on CTCAE Version 3.0) that is deemed by the Investigator related to the investigational agent, occurs within 4 weeks of administration, and is confirmed by the Investigator and Sponsor, or by the DSMB. A toxicity of CTC grade 2 that is deemed by the Investigator related to the investigational agent and occurs within 4 weeks of administration may also be reported as a potential DLT if the Investigator considers it a clinically significant safety concern.

Given the long list of expected toxicities experienced by patients undergoing HSCT, careful determination of attribution will be particularly important (see Section 5.2).

Sites must notify the Sponsor within 24 hours of having knowledge of a potential DLT. In cases where it is not clear whether an event should be considered related, the Investigator should discuss the case with the Sponsor’s medical monitor. Within 24 hours of notification of a potential DLT, the Sponsor will contact the Investigator to discuss the event and together will confirm if it is a DLT. If it is determined that an AE meets all of the criteria for DLT, the subject will discontinue study drug. After receiving treatment for the toxicity (if necessary), the subject will be closely monitored until the toxicity is resolved to no worse than grade 2 severity (grade 1 if reported toxicity is initially grade 2) or is stabilized, at which point he/she will be removed from the study.

If the Investigator and Sponsor cannot agree on the determination of a DLT, the issue will be escalated to the DSMB and the subject will discontinue study drug. Within 7 days of the Sponsor receiving notification of a potential DLT, the DSMB must convene (most likely by teleconference) to determine whether the event is to be classified as a DLT. After receiving treatment for the toxicity (if necessary), the subject will be closely monitored until the

toxicity is resolved to no worse than grade 2 severity (grade 1 if reported toxicity is initially grade 2) or is stabilized, at which point he/she will be removed from the study.

The Sponsor will provide written notification of all DLT determination decisions to the Investigator and DSMB members.

Dose escalation must be delayed until a confirmed DLT has resolved or stabilized at grade 2 (grade 1 if reported toxicity is initially grade 2) or less. All Investigators in the study will be notified of all reported toxicities at each dose level before escalation. The Investigators, Sponsor, and DSMB must agree that proceeding with dose/duration escalation is appropriate before the first subject can be treated in the next study group.

The DSMB will be an independent, interdisciplinary body with four members, including two oncologists, one additional specialist (possibly a cardiologist), and XOMA's biostatistician. The DSMB collectively will have substantial experience in the treatment of subjects undergoing HSCT and in the conduct and monitoring of randomized clinical trials. A full description of the DSMB's responsibilities and procedures will be provided in the DSMB Charter under separate cover.

3.3.3 Definition of Maximum Tolerated Dose

The MTD is defined as the highest dose at which at least six subjects complete the treatment course with no more than one subject experiencing a DLT. If two or more subjects experience a DLT at a given dose level, the MTD will have been exceeded and no additional subjects will be treated at that level. The DSMB will convene to examine the data and review the drug-relatedness of the relevant toxicities in light of the typically complex course and safety experience of HSCT patients. If the DSMB agrees that the relevant toxicities were drug-related and met the other criteria for DLT, no further dose escalation will occur and, depending on the nature of the DLTs, the study may be stopped. Throughout the study, the Sponsor will report any significant findings by the DSMB to the FDA.

3.3.4 Dose Escalation Plan

Subjects will be enrolled sequentially according to the dose escalation diagram shown in Figure 4. If a DLT occurs in any subject, it must be treated, if necessary, and monitored until it stabilizes or recovers to no worse than CTC grade 2 toxicity (grade 1 if reported toxicity is initially grade 2) prior to additional enrollment. Subjects will be monitored for toxicity with assessments listed in Appendix 1 (Schedule of Assessments).

To minimize the chance of simultaneous graft failures possibly due to exposure to opebacan, only two subjects may be simultaneously enrolled in any dose group until one of them successfully engrafts, defined as ANC ≥ 500/µL for three consecutive days, with the first day (the day of engraftment) occurring no later than Day 28. Once engraftment occurs in one of the first two subjects in a dose group, the third subject may be enrolled. When engraftment occurs in a second subject within a dose group, the fourth subject may be enrolled, and when

engraftment occurs in a third subject in a dose group, the fifth subject may be enrolled. Finally, when engraftment occurs in a fourth subject in a dose group, the sixth subject may be enrolled.

After all subjects in a dose group have completed study procedures through Day 28, all available safety information will be reviewed by the DSMB. Dosing may begin for the next dose group at the next higher dose level if no more than one subject in the previous group has experienced a DLT and, in the opinion of the DSMB, there are no clinically significant safety concerns at the previous dose level. If, in the opinion of the DSMB, there are clinically significant safety concerns, the DSMB may recommend that the study be discontinued.

If graft failure occurs in any dose group, that dose group will be expanded to 12 subjects. If a second graft failure occurs in the expanded cohort, the DSMB will determine if the study should be stopped after looking at the data for the two subjects whose grafts failed. As with other dose groups, enrollment in the expanded cohort will be staggered to minimize the chance of multiple graft failures. Only two subjects may be simultaneously enrolled until one of them successfully engrafts, at which time an additional subject may be enrolled.

Figure 4
Dose Escalation Diagram

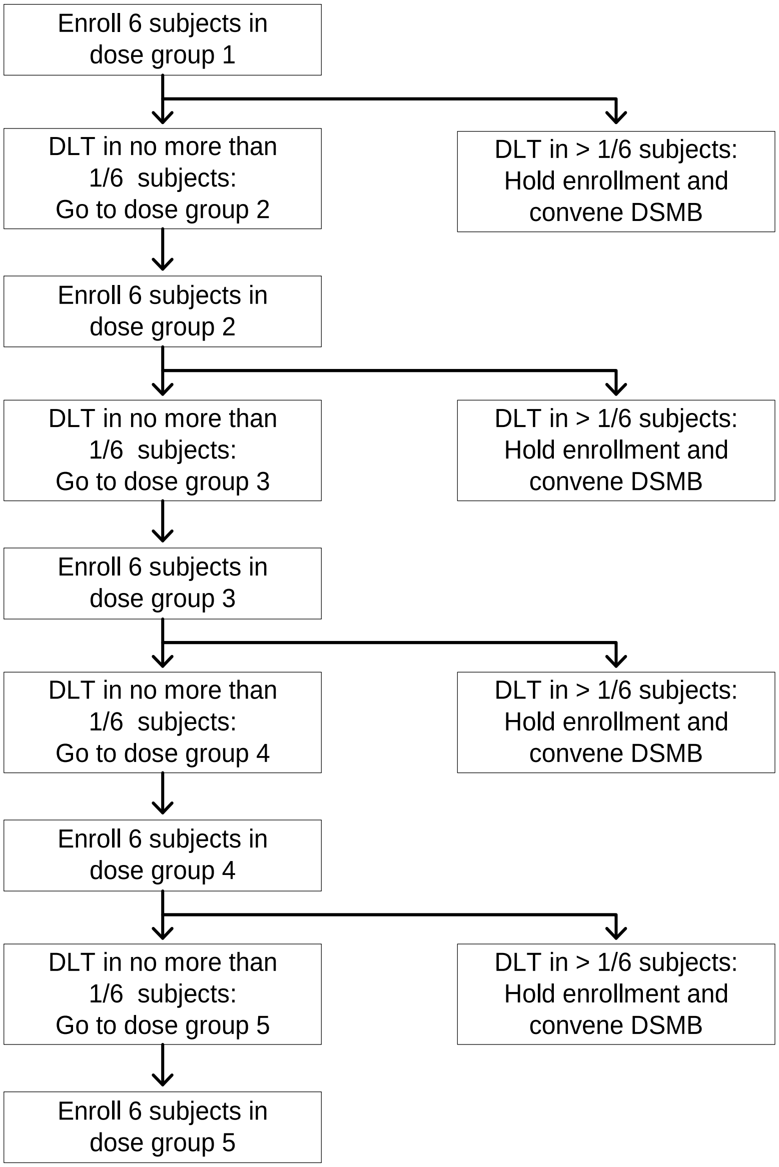

Note: To minimize the chance of simultaneous graft failures possibly due to exposure to opebacan, only two subjects may be simultaneously enrolled in any dose group until one of them successfully engrafts, defined as ANC ≥ 500/µL for three consecutive days, with the first day (the day of engraftment) occurring no later than Day 28. If a subject experiences graft failure, the subject's cohort will be expanded to 12 subjects. If a second graft failure occurs in the expanded cohort, the DSMB will determine whether the study should be stopped after reviewing all available information on the two subjects whose grafts failed.

Table 2 shows the probability of dose escalating assuming different DLT incidence rates. The calculations of these probabilities are based on binomial distributions.

Table 2
Probabilities of Dose Escalating Assuming Different DLT Incidence Rates

Assumed DLT Incidence Rate[1]	Probability of Dose Escalating (DLT in ≤ 1 Subject)[2]
.02 (2%)	.9943
.04 (4%)	.9784
.07 (7%)	.9392
.10 (10%)	.8857
.20 (20%)	.6554
.30 (30%)	.4202
.40 (40%)	.2333
.50 (50%)	.1094

1 The assumed DLT incidence rate is the assumed probability of any single subject experiencing a DLT.

2 The probability of dose escalating is the probability of ≤ 1 of the 6 subjects in a cohort experiencing a DLT.

3.4 Selection of Study Population

Screening for eligibility will proceed in two parts. The first, a prescreen, will establish potential eligibility for treatment while a second, a final screen, will be used to determine whether drug administration will be initiated. Prescreening assessments must be performed within 30 days of admission to the hospital for the conditioning regimen and subsequent HSCT procedure. The final screen must occur on Day −1. If the final screening criteria are met, treatment with opebacan will begin on Day −1, 18 to 36 hours prior to the infusion of donor cells on Day 0.

This protocol is designed for patients undergoing non-T cell depleted myeloablative HSCT. Thus, T cell depletion regimens, including those using Campath or ATG either pre- or post-transplant, are not permitted, and those who have received such regimens are not eligible for enrollment.

3.4.1 Prescreen Eligibility Criteria

3.4.1.1 Prescreen Inclusion Criteria

Subjects may be included in the study if they meet **all** of the following criteria:

- Subject has been informed about the study and signed a XOMA/IRB-approved informed consent prior to performance of any study-related procedures

- Subject consents to an IV catheter inserted and maintained for the purpose of opebacan administration
- Age ≥ 18 and ≤ 60, and undergoing allogeneic HSCT
- Life expectancy ≥ 8 weeks
- Scheduled for treatment with a conditioning regimen intended to be myeloablative, defined as containing one of the following:
  - Busulfan at ≥ 14 mg/kg PO (or IV equivalent)
  - TBI ≥ 1000 cGy
  - Melphalan 180 mg/M^2 (or per kg equivalent)

  (Note: Subjects with Fanconi anemia who are undergoing matched sibling transplant with any radiation or busulfan (IV or PO) regimen are eligible regardless of dose intensity.)
- Meets the following standard performance and end-organ function criteria for stem cell transplantation:
  - Pulmonary function: Room air O_2 saturation > 95% and no clinical evidence of pulmonary insufficiency
  - Renal function: Serum creatinine must be < 1.5× the upper limit of normal for age or, if serum creatinine is elevated beyond normal for age, must have creatinine clearance or GFR > 50% of lower limit of normal for age.
  - Liver function: AST and ALT ≤ 3 times the upper limit of normal for age and total serum bilirubin ≤ 1.5 times the upper limit of normal for age. (Note: In the presence of documented intravascular hemolysis, the direct bilirubin component must be less than 2× the upper limit of normal for age.)
  - ≥ 80% performance score on the Karnofsky Scale; see Appendix 3)
  - Cardiac function: Adequate cardiac function by clinical assessment, normal shortening fraction or ejection fraction for age, and normal cardiac troponin T level (< 0.1 ng/mL)
- Female subjects of child-bearing age must have a negative urine or serum pregnancy test. Sexually active male and female subjects of reproductive age must be using a form of contraception considered effective and medically acceptable by the Investigator.

3.4.1.2 *Prescreen Exclusion Criteria*

Subjects will be excluded from the study if they meet **any** of the following criteria:

- Treatment with T cell depletion regimens, including those using Campath or ATG, either pre- or post-transplant

- Cumulative lifetime exposure of > 300 mg/M^2 of anthracycline (expressed as doxorubicin equivalent dose)
- Active infection. Freedom from active infection is defined as the absence of an infectious diagnosis or (in subjects who have had a recent positive infectious diagnosis) the resolution of fever plus documentation of negative cultures or antigen testing, continuation or completion of a course of appropriate therapy, and the presence of stable to resolving clinical symptoms and/or radiographic signs. The Investigator must document the subject's infectious disease status in the chart prior to enrolling the subject in the study.
- Prophylactic antibacterial antibiotics beyond standard institutional practice. Subjects whose individual risk of infection is so great as to necessitate additional prophylactic administration of systemic antibiotics are excluded. If a subject does not require prophylaxis but the Investigator believes prophylaxis may be advisable, the Investigator should contact the XOMA Medical Monitor before enrolling the subject. Use of gut decontamination is permitted if it is standard institutional practice.
- Positive for HIV
- Any prior stem cell transplant
- Prior history of CHF
- Projected need for heparin anticoagulation during the study medication infusion period. Use of dextran sulfate is also prohibited during the infusion period.
- Enrollment on a chemotherapy regimen where conditioning extends beyond Day −1
- Enrollment on a regimen where it will not be possible to achieve an 18 to 36 hour window between initiation of opebacan and infusion of donor cells.
- Cord blood is the source of a subject's transplanted cells.

3.4.2 Final Pretreatment Eligibility Criteria

3.4.2.1 Final Pretreatment Inclusion Criteria

Subjects may be included in the study if **all** of the following occurs:

1. The Principal Investigator or designee has reviewed and approved subject eligibility for the HSCT prior to proceeding with opebacan administration and confirmed that the interval between initiating opebacan and administering donor cells will be between 18 and 36 hours.
2. All Day −1 pre-dose assessments described in Appendix 1 have been performed. Results for some of these assessments may not be available prior to proceeding with opebacan administration.

3.4.2.2 *Final Pretreatment Exclusion Criteria*

Subjects will be excluded from the study upon re-evaluation at Day −1 if they have evidence of CTC Grade 4 toxicity, including but not limited to any of the following:

- Active infection
- Dialysis dependence
- Need for continuous venovenous hemofiltration
- Requirement for mechanical ventilation
- Veno-occlusive disease
- Requirement for pressor support
- Presence of disseminated intravascular coagulation

3.5 Enrollment

Once prescreening is completed and subject eligibility is confirmed, a subject will be assigned a subject number by the Sponsor. Donors who sign an informed consent will be assigned a tracking number that can be used for tracking donor samples and will link those samples with the subject who received the donor's stem cells.

Sites must contact the following individual for subject number assignment:

Sally Bernard	Fax:	(510) 548-8616
	Phone:	(510) 204-7467

Eligible subjects will be assigned to one of five successive dose groups of six subjects each (see Section 3.1 and Table 1). Enrolled subjects who discontinue the trial prior to receiving opebacan will be replaced. Subjects will also be replaced if they do not receive at least 80% of their scheduled dose of opebacan and/or do not complete assessments for at least 7 days following the end of their opebacan infusion.

3.6 Treatment

3.6.1 Formulation

Opebacan is supplied as a clear, colorless, sterile non-pyrogenic solution in 10 mL single use glass vials at a concentration of 2 mg/mL in 5 mM sodium citrate/0.15 M sodium chloride buffer, pH 5.0 with 0.2% poloxamer 188 and 0.002% polysorbate 80, containing no preservative.

For further details, see the Investigator's Brochure.

3.6.2 *Labeling*

All vial labels will include the information specified in Table 3.

Table 3
Required Label Information

Logo of manufacturer:	XOMA
Protocol number:	BPSC030
Subject number:	[TBD]
Name of study drug:	opebacan, $rBPI_{21}$, NEUPREX®
Instructions:	Sterile solution for intravenous use. See clinical protocol for dosage and administration.
Part number:	12580
Contents:	10 mL (2 mg/mL)
Lot number:	[TBD]
Storage instructions:	Store at 2-8°C (36-46°F)
Caution:	New Drug — Limited by Federal Law to Investigational Use
Manufactured by:	XOMA (US) LLC, Berkeley, CA 94710

3.6.3 *Shipping*

A sufficient number of vials of opebacan for all doses for each subject will be shipped to the study site. Each vial will contain 10 mL of opebacan.

If the packaging is damaged upon receipt or if there is anything unusual about the appearance or attributes of this study drug, the drug should not be used and the problem should be immediately reported to the following individual by phone:

Peter Foote
Manager, Clinical Operations and Data Management
XOMA (US) LLC
Phone: (510) 204-7465

3.6.4 *Warnings*

Humans administered foreign proteins are at risk of developing allergic reactions including anaphylaxis. When an anaphylactoid reaction is suspected, opebacan should be discontinued immediately and appropriate therapy (including oxygen, epinephrine, diphenhydramine, corticosteroids, and volume expansion) should be administered as medically indicated.

Subjects must not receive heparin anticoagulation during the study medication infusion period.

The study drug should be used only by physicians who are qualified by training and experience in the safe use and handling of investigational drugs. Do not use if the solution is turbid or contains particulate matter. No long-term animal studies have been performed with opebacan to determine carcinogenic potential or impairment of fertility in males or females.

3.6.5 Dosage, Administration, and Storage

3.6.5.1 Dosage

All subjects will receive an initial 4 mg/kg continuous IV infusion for 30 minutes. This will be followed immediately by a continuous infusion that varies, according to the dose group, in dose level and duration (see Table 1). Subjects whose conditioning regimen includes TBI will have their opebacan infusion interrupted for 1-hour TBI treatments once on Day −1 and twice on Day 0 prior to the HSCT procedure, which will take place after the opebacan infusion has been restarted for at least 2 hours.

3.6.5.2 Administration

The study drug (opebacan) should be brought to room temperature prior to infusion. Further details of administration are included in the drug administration information sheet. Throughout the dosing procedure, aseptic technique for intravenous administration should be followed. The study drug will be administered by intravenous infusion into a central or peripheral vein. Suitability of intravenous access will be determined by easy withdrawal of blood from the access, as well as easy infusion of intravenous fluids without infiltration.

The study medication must be the sole agent administered in the chosen port during the course of the infusion protocol. The venous access port will not be heparinized, but may be flushed as necessary with physiologic saline for injection. Any sign of a reaction at a site of infusion should be recorded on the subject's CRF and source document as an adverse experience. If the infusion is interrupted, it should be resumed as soon as possible. However, if more than two hours has elapsed, call XOMA's Medical Monitor at (800) 858-2783 before restarting the infusion. Any unexpected attributes or appearance of the investigational drug should be reported to XOMA immediately.

3.6.5.3 Storage and Handling

All study drug must be stored refrigerated at 2-8°C (35.6-46.4°F). The study drug should not be allowed to freeze, should not be shaken, and should be stored upright.

The contents of the vials supplied are sterile and pyrogen free. Aseptic techniques must be used during withdrawal, preparation and administration. Vials contain no preservative and are for single use only.

3.6.6 Prior and Concomitant Therapy

3.6.6.1 Excluded Concomitant Medications

Subjects may not participate in any other investigational drug or device study or receive other phase I agents from the signing of the informed consent form through Day 100. If a subject has a clinical indication for emergent use of any phase I agent, the Principal Investigator at the specific site must be informed and decide with the Sponsor if the subject should be removed from the study. Consistent with good clinical care, non-standard therapies should be avoided during the study period.

Subjects must not receive heparin anticoagulation during the study medication infusion period. Use of dextran sulfate is also prohibited during the infusion period. Subjects who are receiving heparin for routine line care (e.g., a heparin lock or an intermittent small volume bolus) are eligible to receive treatment.

3.6.6.2 Allowed Concomitant Medications

Subjects undergoing HSCT receive a broad range of medications to treat the side effects of the conditioning regimen and transplant, as well as the underlying disease. All concomitant medications used during the study must be recorded on the appropriate page of the CRF. The following list of drug classes encompasses many of the concomitant medications Investigators will administer to subjects and which are acceptable for use during this trial:

- Antihypertensives
- Antibiotics
- Antivirals
- Antifungals
- IVIG
- Blood products
- Hematologic growth factors
- Antacids
- Immunosuppressives
- Phenothiazines
- Antiemetics
- Analgesics
- Electrolyte and mineral supplements
- Nutritional Support

3.7 Supportive Care Guidelines

The following supportive care guidelines must be observed:

- Subjects who are CMV seropositive and/or have seropositive donors must be monitored for CMV weekly using institutional guidelines for assay choice until Day 100. Subjects should be treated for ANY positive antigen or PCR result per institutional practice.
- Subjects must receive pre-emptive PCP treatment during the conditioning regimen according to institutional practice and reinitiate PCP prophylaxis after hematologic recovery. The total duration of PCP prophylaxis will be determined by institutional practice but it should be continued for at least 100 days after transplant. Therapy may be modified for drug intolerance.
- All subjects must receive prophylactic antifungal therapy during transplant. Choice of agents is per institutional practice.
- Any use of hematopoietic growth factors must be recorded in the study documentation. All blood products should be irradiated and administered per institutional practice.
- General support of fluids, electrolytes, nutrition and other medical needs should be per institutional guidelines.
- Subjects must not use heparin anticoagulation during the study medication infusion period. Use of dextran sulfate is also prohibited during the infusion period.
- The study medication must be the sole agent administered in the chosen port during the course of the infusion protocol.

3.8 Outcome Measures

3.8.1 Biological and Clinical Activity

Biological and clinical activity will be assessed through an evaluation of the following outcome measures:

- Time to engraftment, defined as the first of three consecutive daily measurements of ANC $\geq$ 500/µL
- Inflammatory markers (such as LPS, LBP, IL-6, CRP and other exploratory biomarkers)
- Inflammatory states such as presence of fever and infection
- Transplant-related complications including, but not limited to, aGvHD, inadequate oral intake, presence of oral mucositis, veno-occlusive disease of the liver, and idiopathic pneumonitis.

3.8.2 Safety Outcome Measures

Safety will be assessed by pre- and post-treatment serial measurements of vital signs and clinical laboratory assessments and by the recording of adverse clinical events with an emphasis on infectious and non-infectious regimen-related toxicities.

3.8.3 Pharmacokinetic Assessment and Sampling Schedule

The pharmacokinetics of opebacan will be determined from samples collected from each subject at the time points shown in Appendix 2. Note that the timing of PK sampling varies by dosing group. The timing of PK draws for the various dose groups and the acceptable windows for those draws are as follows:

- **Subjects in Dose Groups 1, 2, and 3** will receive 3-day opebacan infusions and will have PK samples collected at prescreening (to measure the level of endogenous BPI), on Day −1 (predose and 30 [± 5 minutes], 45 [± 5 minutes], 90 [± 15 minutes], and 270 [± 30 minutes] minutes following the start of infusion), Day 0 (24 hours [± 60 minutes] following the start of infusion), Day 1 (48 hours [± 60 minutes] following the start of infusion), Day 2 (within 1 hour prior to and 15 [± 5 minutes], 30 [± 5 minutes], 60 [± 10 minutes], and 240 [± 30 minutes] minutes following the end of infusion), Day 3 (24 hours [± 60 minutes] following the end of infusion), and Day 100.
- **Subjects in Dose Group 4** will receive 7-day opebacan infusions and will have PK samples collected at prescreening (to measure the level of endogenous BPI), on Day −1 (predose and 30 [± 5 minutes], 45 [± 5 minutes], 90 [± 15 minutes], and 270 [± 30 minutes] minutes following the start of infusion), Day 0, Day 1, Day 6 (within 1 hour prior to and 15 [± 5 minutes], 30 [± 5 minutes], 60 [± 10 minutes], and 240 [± 30 minutes] minutes following the end of infusion), Day 7 (24 hours [± 60 minutes] following the end of infusion), and Day 100.
- **Subjects in Dose Group 5** will receive 14-day opebacan infusions and will have PK samples collected at prescreening (to measure the level of endogenous BPI), on Day −1 (predose and 30 [± 5 minutes], 45 [± 5 minutes], 90 [± 15 minutes], and 270 [± 30 minutes] minutes following the start of infusion), Day 0, Day 1, Day 6, Day 10, Day 13 (within 1 hour prior to and 15 [± 5 minutes], 30 [± 5 minutes], 60 [± 10 minutes], and 240 [± 30 minutes] minutes following the end of infusion), Day 14 (24 hours [± 60 minutes] following the end of infusion), and Day 100.

3.8.4 Immunogenicity Assessment

All subjects must have samples for anti-opebacan antibody analysis drawn at prescreening. Samples will also be collected at Day 28 ± 3 days to detect whether subjects have developed anti-opebacan antibodies. In addition, donors who consent must have samples drawn for anti-opebacan antibody analysis prior to stem cell harvest.

3.9 Assay Methods

3.9.1 Pharmacokinetics Assay

A sandwich ELISA was developed for the measurement of opebacan in plasma. The ELISA utilizes an affinity-purified rabbit anti-BPI antibody for solid-phase capture and a biotin-labeled rabbit anti-BPI antibody (followed by streptavidin-alkaline phosphatase) as the detector. A standard curve is plotted as A_{405} versus concentration. A four-parameter fit is performed and concentrations are determined for samples and controls by interpolation from the standard curve.

3.9.2 Immunological Assay

An immunoassay will be developed for the measurement of anti-opebacan antibodies in human plasma. The assay will utilize control plasma from either a primate and/or a non-primate species.

4.0 STUDY OPERATIONS AND EVALUATIONS

4.1 Overview

To decrease variability in this study, reassessments requiring interpretation or manual measurements should, as consistently as possible, be made by the same individual performing the original assessment.

4.2 Reporting and Recording of Data

Case Report Forms (CRFs) will be supplied by XOMA and should be completed according to the instructions provided in the CRF Completion Guidelines and ICH/GCP Guidelines.

A CRF must be completed for each subject enrolled under this protocol. Information collected on the screen failure subjects will include subject initials, date of birth, and the reason the subject was not enrolled.

All information collected in the CRF must be verifiable in the source documents. Source documents for this trial may include hospital records, clinic records, and any study-specific worksheets or laboratory result reporting documents. Subject medical records must contain reference to the study title and assigned subject identification number. The signed consent form must be filed with the subject's medical record.

CRF completion must be kept current to reflect subject status during the course of the trial. All CRF data must be reviewed, signed and dated by the Principal Investigator.

All CRFs should be completed in a neat and legible manner to ensure accurate interpretation of data. When making changes or corrections, cross out the original entry with a single line, and initial and date the change. DO NOT ERASE, OVERWRITE, OR USE CORRECTION FLUID ON THE ORIGINAL CRF.

CRF pages will be double data entered at XOMA, after which the data may be manually or electronically reviewed by the clinical team. Any resulting data queries will be sent back to the site for resolution.

Study subjects will be identified by subject ID number, initials, and date of birth. Subject names or other personal identification (e.g., subject social security number) must be blacked out on any documents submitted to XOMA or their designee, with subject ID number, initials and protocol number transcribed onto the upper right hand corner of each document page.

Information on donors who consent, including the donor ID number and the data from assessments performed on the donor, will be entered on the appropriate pages of the CRF of the subject who received that donor's stem cells.

4.3 Study Assessments

Study assessments are shown in the Schedule of Assessments (Appendix 1). Routine laboratory analyses, including chemistries, CBC, coagulation measures, and urinalysis will be performed locally at the study sites. Surface markers requiring same day analysis (e.g., TLR4) will also be processed and analyzed locally, though in some circumstances these samples may be frozen, batched and sent to a central lab. All other samples will be analyzed at a central lab.

The following assessments will be performed daily from Day −1 until Day 49 or until the subject is released from the hospital, whichever occurs first:

- Metabolic assessment (Intake and output [I&O] and total days of parenteral nutrition [TPN] only). Assessments requiring daily totals should be measured by calendar day (i.e., from midnight to midnight) unless otherwise specified by institutional standard practice.
- Vital signs including pulse, blood pressure (systolic/diastolic), respiratory rate, and temperature taken in the morning (i.e., the first waking set of vital signs). If a subject experiences an SAE, vital signs must be taken more frequently, per standard institutional practice.

In addition, the oral mucositis score must be recorded at prescreening and then daily from Day −1 through Day 7 (see Appendix 5 for grading scale). After Day 7, the oral mucositis score must be recorded daily until the condition resolves to grade 0. If oral mucositis reappears after resolving, daily assessments must be reinstituted through Day 49 or until the subject is released from the hospital, whichever occurs first.

All assessments scheduled on Days 14, 21, and 28 must be performed for all subjects whether or not the subjects have been discharged from the hospital. Assessments scheduled on Days 35, 42 and 49 will be performed only for those subjects who are still hospitalized on those days. Note that assessments scheduled between Day 14 and Day 49 (excluding Days 21, 28, 35, and 42) are not shown in the table for Appendix 1 (Study BPSC030 Schedule of Assessments), though they are mentioned in the footnotes for the assessments that are affected (i.e., Footnote 14 [metabolic assessment], Footnote 15 [vital signs], and Footnote 21 [oral mucositis]).

Several of the assessments performed during this study are considered “over and above” those normally performed as part of routine patient care for HSCT procedures. These special assessments are as follows:

- DNA testing (SNPs for markers such as TLR and TNF) at select sites only
- Opebacan blood levels (PK assays)
- Inflammatory markers such as LPS and LBP
- LPS-induced TNF and IL-6 at select sites only

- Soluble markers that may include, but may not be limited to, sCD14, TNF-RI, IL-1, IL-6, IL-8, IL-10, IL-12, and CRP
- Surface markers at select sites only; may include, but may not be limited to, TLR4 and mCD14
- Cardiac serology including CPK-MB, troponin T, and troponin I
- Anti-opebacan antibody response

4.3.1 Screening Assessments

Before any screening evaluations or measurements are performed, subjects must be fully informed and sign a XOMA/IRB-approved informed consent form for participation in this study. All screening evaluations must be performed prior to the start of study drug dosing (Day −1).

Prescreening (Within 30 Days of Hospital Admission for Conditioning, HSCT)

- Signed XOMA/IRB-approved informed consent
- Review of inclusion and exclusion criteria
- Demographic information (sex, age, race/ethnicity)
- Medical history, including virology and serology status
- HIV antibody
- ECG
- Urine or serum pregnancy test for women of childbearing potential only
- DNA testing (SNPs for markers such as TLR and TNF) at select sites only
- Review and recording of concomitant medications
- PK sample for measuring level of endogenous BPI
- Laboratory evaluations:
    - Chemistries: Sodium, potassium, chloride, bicarbonate, glucose, BUN, creatinine, calcium, phosphorus, magnesium, bilirubin (total, and direct if bilirubin > 2.0 mg/dL), albumin, ALT, AST, LDH, alkaline phosphatase, and total protein
    - Coagulation: PT, PTT, fibrinogen, and D-Dimers (or FDP or FSP)
    - Urinalysis: Chemistry and microscopic examination
    - Hematology: CBC with differential and platelet count
- Physical examination

- Vital signs including pulse, blood pressure (systolic/diastolic), respiratory rate, and temperature taken in the morning (i.e., the first waking set of vital signs). If a subject experiences an SAE, vital signs must be taken more frequently, per standard institutional practice. (Note: Vital signs must be recorded daily from prescreening through Day 49 or until the subject is released from the hospital, whichever occurs first.)
- LPS and LBP
- LPS-induced TNF and IL-6 at select sites only
- Oral mucositis score (see Appendix 5)
- Cardiac serology (CPK-MB, cardiac troponin T, and troponin I)
- LVEF by ECHO or MUGA
- Anti-opebacan antibody response

Day –3 (All Subjects)

- LPS
- Surface markers at select sites only; may include, but may not be limited to, TLR4 and mCD14

Final Screening (Day –1)

The following final screen assessments must be completed prior to dosing with opebacan:

- Review of inclusion and exclusion criteria
- Interim medical history
- Metabolic assessment. The subject or legal guardian will be asked to provide the subject's recent dietary history.
- Review and recording of concomitant medications
- Confirmation and documentation by the Principal Investigator or designee that the subject meets all eligibility criteria for the HSCT and that the initiation of study drug administration will precede donor cell administration by the appropriate 18 to 36 hours
- Approval of initiation of drug administration by the Principal Investigator or designee

4.3.2 *Assessments for Treatment and Follow-up Period*

If a subject has a clinically significant abnormal laboratory test value that was not present at baseline (Day −1 prior to opebacan dosing), the test will be repeated and the subject will be followed until the test value has returned to the baseline range or the Investigator has determined that the abnormality is chronic or stable.

4.3.2.1 *Assessments for Treatment and Follow-up Period: Dose Groups 1, 2, and 3*

Day −1 (Dose Groups 1, 2, and 3)

- Interim medical history (predose)
- Adverse events (Note: The “signs and symptoms inventory” CRF page must be completed predose and all AEs collected postdose.)
- Review and recording of concomitant medications
- PK sample (predose and 30, 45, 90, and 270 minutes after the start of infusion). Note: PK sample must be drawn from a different vein than that used for opebacan administration.
- Laboratory evaluations (predose):
  - Chemistries: Sodium, potassium, chloride, bicarbonate, glucose, BUN, creatinine, calcium, phosphorus, magnesium, bilirubin (total, and direct if bilirubin > 2.0 mg/dL), albumin, ALT, AST, LDH, alkaline phosphatase, and total protein
  - Coagulation: PT, PTT, fibrinogen, and D-Dimers (or FDP or FSP)
  - Urinalysis: Chemistry and microscopic examination
  - Hematology: CBC with differential and platelet count
- Metabolic assessment (Caloric source documentation including volume and type for all fluids and nutritional sources, intake and output [I&O], and total days of parenteral nutrition [TPN]). Assessments requiring daily totals should be measured by calendar day (i.e., from midnight to midnight) unless otherwise specified by institutional standard practice.
- Vital signs including pulse, blood pressure (systolic/diastolic), respiratory rate, and temperature. Temperature is measured twice: predose (within one hour prior to the start of infusion) and postdose (within four hours after the start of infusion). In addition, body weight must be measured predose to calculate the study drug dose. If a subject experiences an SAE, vital signs must be taken more frequently, per standard institutional practice.
- Fever (T_{max}) measured for two separate periods: predose (within one hour prior to the start of infusion) and postdose (within four hours after the start of infusion).
- LPS and LBP (predose)

- LPS-induced TNF and IL-6 (predose) at select sites only
- Soluble markers (predose) that may include, but may not be limited to, sCD14, TNF-RI, IL-1, IL-6, IL-8, IL-10, IL-12, and CRP
- Surface markers (predose) at select sites only; may include, but may not be limited to, TLR4 and mCD14
- Oral mucositis score (predose; see Appendix 5)
- Cardiac serology (CPK-MB, cardiac troponin T, and troponin I) (predose)
- LVEF by ECHO or MUGA (predose)
- Performance score (predose using Karnofsky criteria; see Appendix 3)
- Initiation of opebacan dosing. Subjects whose conditioning regimen includes TBI will have their opebacan infusion interrupted for a 1-hour TBI treatment.

Day 0 (Day of HSCT) (Dose Groups 1, 2, and 3)

- Adverse events
- Concomitant medications
- PK sample (24 hours after start of infusion). Note: PK sample must be drawn from a different vein than that used for opebacan administration.
- Laboratory evaluations:
    - Hematology: CBC with differential and platelet count
- Metabolic assessment (Caloric source documentation including volume and type for all fluids and nutritional sources, intake and output [I&O], and total days of parenteral nutrition [TPN]). Assessments requiring daily totals should be measured by calendar day (i.e., from midnight to midnight) unless otherwise specified by institutional standard practice.
- Vital signs including pulse, blood pressure (systolic/diastolic), respiratory rate, and temperature taken in the morning (i.e., the first waking set of vital signs). If a subject experiences an SAE, vital signs must be taken more frequently, per standard institutional practice.
- Fever (T_{max})
- LPS and LBP
- Soluble markers that may include, but may not be limited to, sCD14, TNF-RI, IL-1, IL-6, IL-8, IL-10, IL-12, and CRP
- Surface markers at select sites only; may include, but may not be limited to, TLR4 and mCD14
- Oral mucositis score (see Appendix 5)

- Continuation of opebacan dosing. Subjects whose conditioning regimen includes TBI will have their opebacan infusion interrupted for 1-hour TBI treatments twice on Day 0 prior to the HSCT procedure. The HSCT will take place after the opebacan infusion has been restarted for at least 2 hours.

Day 1 (Dose Groups 1, 2, and 3)

- Adverse events
- Concomitant medications
- PK sample. Note: PK sample must be drawn from a different vein than that used for opebacan administration.
- Metabolic assessment (Intake and output [I&O] and total days of parenteral nutrition [TPN] only). Assessments requiring daily totals should be measured by calendar day (i.e., from midnight to midnight) unless otherwise specified by institutional standard practice.
- Vital signs including pulse, blood pressure (systolic/diastolic), respiratory rate, and temperature taken in the morning (i.e., the first waking set of vital signs). If a subject experiences an SAE, vital signs must be taken more frequently, per standard institutional practice.
- Fever (T_{max})
- Oral mucositis score (see Appendix 5)
- Cardiac serology (CPK-MB, cardiac troponin T, and troponin I)
- Continuation of opebacan dosing

Day 2 (Dose Groups 1, 2, and 3)

Unless otherwise noted, the following assessments should be performed immediately after the end of the opebacan infusion:

- Adverse events
- Concomitant medications
- PK sample (prior to end of infusion and 15, 30, 60, and 240 minutes after the end of infusion). Note: PK sample must be drawn from a different vein than that used for opebacan administration.
- Laboratory evaluations
  - Hematology: CBC with differential and platelet count
- Metabolic assessment (Intake and output [I&O] and total days of parenteral nutrition [TPN] only). Assessments requiring daily totals should be measured by

calendar day (i.e., from midnight to midnight) unless otherwise specified by institutional standard practice.

- Vital signs including pulse, blood pressure (systolic/diastolic), respiratory rate, and temperature taken in the morning (i.e., the first waking set of vital signs). If a subject experiences an SAE, vital signs must be taken more frequently, per standard institutional practice.
- Fever (T_{max})
- LPS and LBP
- Soluble markers that may include, but may not be limited to, sCD14, TNF-RI, IL-1, IL-6, IL-8, IL-10, IL-12, and CRP
- Surface markers at select sites only; may include, but may not be limited to, TLR4 and mCD14
- Oral mucositis score (see Appendix 5)
- End of opebacan dosing

Day 3 (Dose Groups 1, 2, and 3)

- Adverse events
- Concomitant medications
- PK sample (24 hours after the end of infusion). Note: PK sample must be drawn from a different vein than that used for opebacan administration.
- Laboratory evaluations:
  - Chemistries: Sodium, potassium, chloride, bicarbonate, glucose, BUN, creatinine, calcium, phosphorus, magnesium, bilirubin (total, and direct if bilirubin > 2.0 mg/dL), albumin, ALT, AST, LDH, alkaline phosphatase, CPK (with MB fraction), and total protein
  - Coagulation: PT, PTT, fibrinogen, and D-Dimers (or FDP or FSP)
  - Urinalysis: Chemistry and microscopic examination
  - Hematology: CBC with differential and platelet count
- Metabolic assessment (Caloric source documentation including volume and type for all fluids and nutritional sources, intake and output [I&O], and total days of parenteral nutrition [TPN]). Assessments requiring daily totals should be measured by calendar day (i.e., from midnight to midnight) unless otherwise specified by institutional standard practice.
- Vital signs including pulse, blood pressure (systolic/diastolic), respiratory rate, and temperature taken in the morning (i.e., the first waking set of vital signs). If a subject experiences an SAE, vital signs must be taken more frequently, per standard institutional practice.

- Fever (T_{max})
- Oral mucositis score (see Appendix 5)

Day 4 (Dose Groups 1, 2, and 3)

- Adverse events
- Concomitant medications
- Metabolic assessment (Intake and output [I&O] and total days of parenteral nutrition [TPN] only). Assessments requiring daily totals should be measured by calendar day (i.e., from midnight to midnight) unless otherwise specified by institutional standard practice.
- Vital signs including pulse, blood pressure (systolic/diastolic), respiratory rate, and temperature taken in the morning (i.e., the first waking set of vital signs). If a subject experiences an SAE, vital signs must be taken more frequently, per standard institutional practice.
- Fever (T_{max})
- LPS and LBP
- Oral mucositis score (see Appendix 5)
- Cardiac serology (CPK-MB, cardiac troponin T, and troponin I)

Day 5 (Dose Groups 1, 2, and 3)

- Adverse events
- Concomitant medications
- Metabolic assessment (Intake and output [I&O] and total days of parenteral nutrition [TPN] only). Assessments requiring daily totals should be measured by calendar day (i.e., from midnight to midnight) unless otherwise specified by institutional standard practice.
- Vital signs including pulse, blood pressure (systolic/diastolic), respiratory rate, and temperature taken in the morning (i.e., the first waking set of vital signs). If a subject experiences an SAE, vital signs must be taken more frequently, per standard institutional practice.
- Fever (T_{max})
- Oral mucositis score (see Appendix 5)

Day 6 (Dose Groups 1, 2, and 3)

- Adverse events

- Concomitant medications
- Metabolic assessment (Intake and output [I&O] and total days of parenteral nutrition [TPN] only). Assessments requiring daily totals should be measured by calendar day (i.e., from midnight to midnight) unless otherwise specified by institutional standard practice.
- Vital signs including pulse, blood pressure (systolic/diastolic), respiratory rate, and temperature taken in the morning (i.e., the first waking set of vital signs). If a subject experiences an SAE, vital signs must be taken more frequently, per standard institutional practice.
- Fever (T_{max})
- LPS and LBP
- Oral mucositis score (see Appendix 5)

Day 7 (Dose Groups 1, 2, and 3)

- Adverse events
- Concomitant medications
- Laboratory evaluations:
  - Hematology: CBC with differential and platelet count
- Metabolic assessment (Caloric source documentation including volume and type for all fluids and nutritional sources, intake and output [I&O], and total days of parenteral nutrition [TPN]). Assessments requiring daily totals should be measured by calendar day (i.e., from midnight to midnight) unless otherwise specified by institutional standard practice.
- Vital signs including pulse, blood pressure (systolic/diastolic), respiratory rate, and temperature taken in the morning (i.e., the first waking set of vital signs). If a subject experiences an SAE, vital signs must be taken more frequently, per standard institutional practice.
- Fever (T_{max})
- Soluble markers that may include, but may not be limited to, sCD14, TNF-RI, IL-1, IL-6, IL-8, IL-10, IL-12, and CRP
- Surface markers at select sites only; may include, but may not be limited to, TLR4 and mCD14
- Oral mucositis score (see Appendix 5)
- LVEF by ECHO or MUGA
- Grading for aGVHD (see Appendix 4)
- Performance score (Karnofsky criteria; see Appendix 3)

Day 8 (Dose Groups 1, 2, and 3)

- Adverse events
- Concomitant medications
- Metabolic assessment (Intake and output [I&O] and total days of parenteral nutrition [TPN] only). Assessments requiring daily totals should be measured by calendar day (i.e., from midnight to midnight) unless otherwise specified by institutional standard practice.
- Vital signs including pulse, blood pressure (systolic/diastolic), respiratory rate, and temperature taken in the morning (i.e., the first waking set of vital signs). If a subject experiences an SAE, vital signs must be taken more frequently, per standard institutional practice.
- Fever (T_{max})
- LPS and LBP
- Oral mucositis score recorded daily until condition resolves to grade 0 (see Appendix 5)
- Cardiac serology (CPK-MB, cardiac troponin T, and troponin I)

Day 9 (Dose Groups 1, 2, and 3)

- Adverse events
- Concomitant medications
- Metabolic assessment (Intake and output [I&O] and total days of parenteral nutrition [TPN] only). Assessments requiring daily totals should be measured by calendar day (i.e., from midnight to midnight) unless otherwise specified by institutional standard practice.
- Vital signs including pulse, blood pressure (systolic/diastolic), respiratory rate, and temperature taken in the morning (i.e., the first waking set of vital signs). If a subject experiences an SAE, vital signs must be taken more frequently, per standard institutional practice.
- Fever (T_{max})
- Oral mucositis score recorded daily until condition resolves to grade 0 (see Appendix 5)

Day 10 (Dose Groups 1, 2, and 3)

- Adverse events

- Concomitant medications
- Laboratory evaluations:
  - Hematology: CBC with differential and platelet count
- Metabolic assessment (Caloric source documentation including volume and type for all fluids and nutritional sources, intake and output [I&O], and total days of parenteral nutrition [TPN]). Assessments requiring daily totals should be measured by calendar day (i.e., from midnight to midnight) unless otherwise specified by institutional standard practice.
- Vital signs including pulse, blood pressure (systolic/diastolic), respiratory rate, and temperature taken in the morning (i.e., the first waking set of vital signs). If a subject experiences an SAE, vital signs must be taken more frequently, per standard institutional practice.
- Fever (T_{max})
- LPS and LBP
- Oral mucositis score recorded daily until condition resolves to grade 0 (see Appendix 5)

Day 11 (Dose Groups 1, 2, and 3)

- Adverse events
- Concomitant medications
- Metabolic assessment (Intake and output [I&O] and total days of parenteral nutrition [TPN] only). Assessments requiring daily totals should be measured by calendar day (i.e., from midnight to midnight) unless otherwise specified by institutional standard practice.
- Vital signs including pulse, blood pressure (systolic/diastolic), respiratory rate, and temperature taken in the morning (i.e., the first waking set of vital signs). If a subject experiences an SAE, vital signs must be taken more frequently, per standard institutional practice.
- Fever (T_{max})
- Oral mucositis score recorded daily until condition resolves to grade 0 (see Appendix 5)

Day 12 (Dose Groups 1, 2, and 3)

- Adverse events
- Concomitant medications

- Metabolic assessment (Intake and output [I&O] and total days of parenteral nutrition [TPN] only). Assessments requiring daily totals should be measured by calendar day (i.e., from midnight to midnight) unless otherwise specified by institutional standard practice.
- Vital signs including pulse, blood pressure (systolic/diastolic), respiratory rate, and temperature taken in the morning (i.e., the first waking set of vital signs). If a subject experiences an SAE, vital signs must be taken more frequently, per standard institutional practice.
- Fever (T_{max})
- Oral mucositis score recorded daily until condition resolves to grade 0 (see Appendix 5)

Day 13 (Dose Groups 1, 2, and 3)

- Adverse events
- Concomitant medications
- Metabolic assessment (Intake and output [I&O] and total days of parenteral nutrition [TPN] only). Assessments requiring daily totals should be measured by calendar day (i.e., from midnight to midnight) unless otherwise specified by institutional standard practice.
- Vital signs including pulse, blood pressure (systolic/diastolic), respiratory rate, and temperature taken in the morning (i.e., the first waking set of vital signs). If a subject experiences an SAE, vital signs must be taken more frequently, per standard institutional practice.
- Fever (T_{max})
- Oral mucositis score recorded daily until condition resolves to grade 0 (see Appendix 5)

Day 14 (Dose Groups 1, 2, and 3)

- Adverse events
- Concomitant medications
- Metabolic assessment (Caloric source documentation including volume and type for all fluids and nutritional sources, intake and output [I&O], and total days of parenteral nutrition [TPN]). Assessments requiring daily totals should be measured by calendar day (i.e., from midnight to midnight) unless otherwise specified by institutional standard practice.
- Vital signs including pulse, blood pressure (systolic/diastolic), respiratory rate, and temperature taken in the morning (i.e., the first waking set of vital signs). If a

subject experiences an SAE, vital signs must be taken more frequently, per standard institutional practice.

- Laboratory evaluations:
  - Chemistries: Sodium, potassium, chloride, bicarbonate, glucose, BUN, creatinine, calcium, phosphorus, magnesium, bilirubin (total, and direct if bilirubin > 2.0 mg/dL), albumin, ALT, AST, LDH, alkaline phosphatase, and total protein
  - Coagulation: PT, PTT, fibrinogen, and D-Dimers (or FDP or FSP)
  - Urinalysis: Chemistry and microscopic examination
  - Hematology: CBC with differential and platelet count
- LPS and LBP
- LPS-induced TNF and IL-6 at select sites only
- Soluble markers that may include, but may not be limited to, sCD14, TNF-RI, IL-1, IL-6, IL-8, IL-10, IL-12, and CRP
- Surface markers at select sites only; may include, but may not be limited to, TLR4 and mCD14
- Oral mucositis score recorded daily until condition resolves to grade 0 (see Appendix 5)
- Cardiac serology (CPK-MB, cardiac troponin T, and troponin I)
- LVEF by ECHO or MUGA
- Grading for aGVHD (see Appendix 4)
- Performance score (Karnofsky criteria; see Appendix 3)

Day 15 through Day 20 (Dose Groups 1, 2, and 3)

Unless otherwise noted, the following assessments will be performed daily until the subject is released from the hospital:

- Metabolic assessment (Intake and output [I&O] and total days of parenteral nutrition [TPN]; on Day 17 only, caloric source documentation including volume and type for all fluids and nutritional sources). Assessments requiring daily totals should be measured by calendar day (i.e., from midnight to midnight) unless otherwise specified by institutional standard practice.
- Vital signs including pulse, blood pressure (systolic/diastolic), respiratory rate, and temperature taken in the morning (i.e., the first waking set of vital signs). If a subject experiences an SAE, vital signs must be taken more frequently, per standard institutional practice.

- Oral mucositis score recorded daily until condition resolves to grade 0 (see Appendix 5)

Day 21 (± 3 days) (Dose Groups 1, 2, and 3)

- Adverse events
- Concomitant medications
- Metabolic assessment (Caloric source documentation including volume and type for all fluids and nutritional sources, intake and output [I&O], and total days of parenteral nutrition [TPN]). Assessments requiring daily totals should be measured by calendar day (i.e., from midnight to midnight) unless otherwise specified by institutional standard practice.
- Vital signs including pulse, blood pressure (systolic/diastolic), respiratory rate, and temperature taken in the morning (i.e., the first waking set of vital signs). If a subject experiences an SAE, vital signs must be taken more frequently, per standard institutional practice.
- Laboratory evaluations:
  - Chemistries: Sodium, potassium, chloride, bicarbonate, glucose, BUN, creatinine, calcium, phosphorus, magnesium, bilirubin (total, and direct if bilirubin > 2.0 mg/dL), albumin, ALT, AST, LDH, alkaline phosphatase, and total protein
  - Coagulation: PT, PTT, fibrinogen, and D-Dimers (or FDP or FSP)
  - Urinalysis: Chemistry and microscopic examination
  - Hematology: CBC with differential and platelet count
- LPS and LBP
- LPS-induced TNF and IL-6 at select sites only
- Soluble markers that may include, but may not be limited to, sCD14, TNF-RI, IL-1, IL-6, IL-8, IL-10, IL-12, and CRP
- Surface markers at select sites only; may include, but may not be limited to, TLR4 and mCD14
- Oral mucositis score recorded daily until condition resolves to grade 0 (see Appendix 5)
- Cardiac serology (CPK-MB, cardiac troponin T, and troponin I)
- LVEF by ECHO or MUGA
- Grading for aGVHD (see Appendix 4)
- Performance score (Karnofsky criteria; see Appendix 3)

Day 22 through Day 27 (Dose Groups 1, 2, and 3)

Unless otherwise noted, the following assessments will be performed daily until the subject is released from the hospital:

- Metabolic assessment (Intake and output [I&O] and total days of parenteral nutrition [TPN]; on Day 24 only, caloric source documentation including volume and type for all fluids and nutritional sources). Assessments requiring daily totals should be measured by calendar day (i.e., from midnight to midnight) unless otherwise specified by institutional standard practice.
- Vital signs including pulse, blood pressure (systolic/diastolic), respiratory rate, and temperature taken in the morning (i.e., the first waking set of vital signs). If a subject experiences an SAE, vital signs must be taken more frequently, per standard institutional practice.
- Oral mucositis score recorded daily until condition resolves to grade 0 (see Appendix 5)

Day 28 (± 3 days) (Dose Groups 1, 2, and 3)

- Adverse events
- Concomitant medications
- Laboratory evaluations:
  - Chemistries: Sodium, potassium, chloride, bicarbonate, glucose, BUN, creatinine, calcium, phosphorus, magnesium, bilirubin (total, and direct if bilirubin > 2.0 mg/dL), albumin, ALT, AST, LDH, alkaline phosphatase, and total protein
  - Coagulation: PT, PTT, fibrinogen, and D-Dimers (or FDP or FSP)
  - Urinalysis: Chemistry and microscopic examination
  - Hematology: CBC with differential and platelet count
- Metabolic assessment (Caloric source documentation including volume and type for all fluids and nutritional sources, intake and output [I&O], and total days of parenteral nutrition [TPN]). Assessments requiring daily totals should be measured by calendar day (i.e., from midnight to midnight) unless otherwise specified by institutional standard practice.
- Physical exam
- Vital signs including pulse, blood pressure (systolic/diastolic), respiratory rate, and temperature taken in the morning (i.e., the first waking set of vital signs). If a subject experiences an SAE, vital signs must be taken more frequently, per standard institutional practice.
- LPS and LBP

- LPS-induced TNF and IL-6 at select sites only
- Soluble markers that may include, but may not be limited to, sCD14, TNF-RI, IL-1, IL-6, IL-8, IL-10, IL-12, and CRP
- Surface markers at select sites only; may include, but may not be limited to, TLR4 and mCD14
- Oral mucositis score recorded daily until condition resolves to grade 0 (see Appendix 5)
- Cardiac serology (CPK-MB, cardiac troponin T, and troponin I)
- LVEF by ECHO or MUGA
- Grading for aGVHD (see Appendix 4)
- Performance score (Karnofsky criteria; see Appendix 3)
- Anti-opebacan antibody response

Day 29 through Day 34 (Dose Groups 1, 2, and 3)

The following assessments will be performed daily until the subject is released from the hospital:

- Metabolic assessment (Intake and output [I&O] and total days of parenteral nutrition [TPN] only). Assessments requiring daily totals should be measured by calendar day (i.e., from midnight to midnight) unless otherwise specified by institutional standard practice.
- Vital signs including pulse, blood pressure (systolic/diastolic), respiratory rate, and temperature taken in the morning (i.e., the first waking set of vital signs). If a subject experiences an SAE, vital signs must be taken more frequently, per standard institutional practice.
- Oral mucositis score recorded daily until condition resolves to grade 0 (see Appendix 5)

Day 35 (± 3 days) (Dose Groups 1, 2, and 3)

The following assessments will be performed only on subjects who are still hospitalized:

- Adverse events
- Concomitant medications
- Laboratory evaluations:
  - Hematology: CBC with differential and platelet count
- Metabolic assessment (Caloric source documentation including volume and type for all fluids and nutritional sources, intake and output [I&O], and total days of

parenteral nutrition [TPN]). Assessments requiring daily totals should be measured by calendar day (i.e., from midnight to midnight) unless otherwise specified by institutional standard practice.

- Vital signs including pulse, blood pressure (systolic/diastolic), respiratory rate, and temperature taken in the morning (i.e., the first waking set of vital signs). If a subject experiences an SAE, vital signs must be taken more frequently, per standard institutional practice.
- LPS and LBP
- Soluble markers that may include, but may not be limited to, sCD14, TNF-RI, IL-1, IL-6, IL-8, IL-10, IL-12, and CRP
- Surface markers at select sites only; may include, but may not be limited to, TLR4 and mCD14
- Oral mucositis score recorded daily until condition resolves to grade 0 (see Appendix 5)
- Cardiac serology (CPK-MB, cardiac troponin T, and troponin I)
- Performance score (Karnofsky criteria; see Appendix 3)

Day 36 through Day 41 (Dose Groups 1, 2, and 3)

The following assessments will be performed daily until the subject is released from the hospital:

- Metabolic assessment (Intake and output [I&O] and total days of parenteral nutrition [TPN] only). Assessments requiring daily totals should be measured by calendar day (i.e., from midnight to midnight) unless otherwise specified by institutional standard practice.
- Vital signs including pulse, blood pressure (systolic/diastolic), respiratory rate, and temperature taken in the morning (i.e., the first waking set of vital signs). If a subject experiences an SAE, vital signs must be taken more frequently, per standard institutional practice.
- Oral mucositis score recorded daily until condition resolves to grade 0 (see Appendix 5)

Day 42 (± 3 days) (Dose Groups 1, 2, and 3)

The following assessments will be performed only on subjects who are still hospitalized:

- Adverse events
- Concomitant medications
- Laboratory evaluations:

  - Hematology: CBC with differential and platelet count

- Metabolic assessment (Caloric source documentation including volume and type for all fluids and nutritional sources, intake and output [I&O], and total days of parenteral nutrition [TPN]). Assessments requiring daily totals should be measured by calendar day (i.e., from midnight to midnight) unless otherwise specified by institutional standard practice.
- Vital signs including pulse, blood pressure (systolic/diastolic), respiratory rate, and temperature taken in the morning (i.e., the first waking set of vital signs). If a subject experiences an SAE, vital signs must be taken more frequently, per standard institutional practice.
- Oral mucositis score recorded daily until condition resolves to grade 0 (see Appendix 5)
- Cardiac serology (CPK-MB, cardiac troponin T, and troponin I)
- Grading for aGVHD (see Appendix 4)
- Performance score (Karnofsky criteria; see Appendix 3)

Day 43 through Day 48 (Dose Groups 1, 2, and 3)

The following assessments will be performed daily until the subject is released from the hospital:

- Metabolic assessment (Intake and output [I&O] and total days of parenteral nutrition [TPN] only). Assessments requiring daily totals should be measured by calendar day (i.e., from midnight to midnight) unless otherwise specified by institutional standard practice.
- Vital signs including pulse, blood pressure (systolic/diastolic), respiratory rate, and temperature taken in the morning (i.e., the first waking set of vital signs). If a subject experiences an SAE, vital signs must be taken more frequently, per standard institutional practice.
- Oral mucositis score recorded daily until condition resolves to grade 0 (see Appendix 5)

Day 49 (± 3 days) (Dose Groups 1, 2, and 3)

The following assessments will be performed only on subjects who are still hospitalized:

- Adverse events
- Concomitant medications
- Laboratory evaluations:
  - Hematology: CBC with differential and platelet count

- Metabolic assessment (Caloric source documentation including volume and type for all fluids and nutritional sources, intake and output [I&O], and total days of parenteral nutrition [TPN]). Assessments requiring daily totals should be measured by calendar day (i.e., from midnight to midnight) unless otherwise specified by institutional standard practice.
- Vital signs including pulse, blood pressure (systolic/diastolic), respiratory rate, and temperature taken in the morning (i.e., the first waking set of vital signs). If a subject experiences an SAE, vital signs must be taken more frequently, per standard institutional practice.
- Oral mucositis score recorded daily until condition resolves to grade 0 (see Appendix 5)
- Cardiac serology (CPK-MB, cardiac troponin T, and troponin I)
- Performance score (Karnofsky criteria; see Appendix 3)

Day 100 (± 14 days) and/or ≥ Grade 2 aGvHD Diagnosis (Dose Groups 1, 2, and 3)

- Adverse events
- Concomitant medications
- PK sample
- Laboratory evaluations:
  - Chemistries: Sodium, potassium, chloride, bicarbonate, glucose, BUN, creatinine, calcium, phosphorus, magnesium, bilirubin (total, and direct if bilirubin > 2.0 mg/dL), albumin, ALT, AST, LDH, alkaline phosphatase, CPK (with MB fraction), and total protein
  - Coagulation: PT, PTT, fibrinogen, and D-Dimers (or FDP or FSP)
  - Urinalysis: Chemistry and microscopic examination
  - Hematology: CBC with differential and platelet count
- Physical exam
- Vital signs including pulse, blood pressure (systolic/diastolic), respiratory rate, and temperature taken in the morning (i.e., the first waking set of vital signs). If a subject experiences an SAE, vital signs must be taken more frequently, per standard institutional practice.
- LPS and LBP
- LPS-induced TNF and IL-6 at select sites only
- Soluble markers that may include, but may not be limited to, sCD14, TNF-RI, IL-1, IL-6, IL-8, IL-10, IL-12, and CRP

- Surface markers at select sites only; may include, but may not be limited to, TLR4 and mCD14
- LVEF by ECHO or MUGA
- Grading for aGVHD (see Appendix 4)

Hospital Discharge and/or Early Termination (Dose Groups 1, 2, and 3)

- Adverse Events
- Concomitant medications
- Metabolic assessment (Caloric source documentation including volume and type for all fluids and nutritional sources, intake and output [I&O], and total days of parenteral nutrition [TPN]). Assessments requiring daily totals should be measured by calendar day (i.e., from midnight to midnight) unless otherwise specified by institutional standard practice.
- Physical exam
- Vital signs including pulse, blood pressure (systolic/diastolic), respiratory rate, and temperature taken in the morning (i.e., the first waking set of vital signs). If a subject experiences an SAE, vital signs must be taken more frequently, per standard institutional practice.

4.3.2.2 Assessments for Treatment and Follow-up Period: Dose Group 4

Day −1 (Dose Group 4)

- Interim medical history (predose)
- Adverse events (Note: The “signs and symptoms inventory” CRF page must be completed predose and all AEs collected postdose.)
- Review and recording of concomitant medications
- PK sample (predose and 30, 45, 90, and 270 minutes after the start of infusion). Note: PK sample must be drawn from a different vein than that used for opebacan administration.
- Laboratory evaluations (predose):
  - Chemistries: Sodium, potassium, chloride, bicarbonate, glucose, BUN, creatinine, calcium, phosphorus, magnesium, bilirubin (total, and direct if bilirubin > 2.0 mg/dL), albumin, ALT, AST, LDH, alkaline phosphatase, and total protein
  - Coagulation: PT, PTT, fibrinogen, and D-Dimers (or FDP or FSP)
  - Urinalysis: Chemistry and microscopic examination
  - Hematology: CBC with differential and platelet count

- Metabolic assessment (Caloric source documentation including volume and type for all fluids and nutritional sources, intake and output [I&O], and total days of parenteral nutrition [TPN]). Assessments requiring daily totals should be measured by calendar day (i.e., from midnight to midnight) unless otherwise specified by institutional standard practice.
- Vital signs including pulse, blood pressure (systolic/diastolic), respiratory rate, and temperature. Temperature is measured twice: predose (within one hour prior to the start of infusion) and postdose (within four hours after the start of infusion). In addition, body weight must be measured predose to calculate the study drug dose. If a subject experiences an SAE, vital signs must be taken more frequently, per standard institutional practice.
- Fever (T_{max}) measured for two separate periods: predose (within one hour prior to the start of infusion) and postdose (within four hours after the start of infusion).
- LPS and LBP
- LPS-induced TNF and IL-6 (predose) at select sites only
- Soluble markers (predose) that may include, but may not be limited to, sCD14, TNF-RI, IL-1, IL-6, IL-8, IL-10, IL-12, and CRP
- Surface markers (predose) at select sites only; may include, but may not be limited to, TLR4 and mCD14
- Oral mucositis score (predose; see Appendix 5)
- Cardiac serology (CPK-MB, cardiac troponin T, and troponin I) (predose)
- LVEF by ECHO or MUGA (predose)
- Performance score (predose using Karnofsky criteria; see Appendix 3)
- Initiation of opebacan dosing. Subjects whose conditioning regimen includes TBI will have their opebacan infusion interrupted for a 1-hour TBI treatment.

Day 0 (Day of HSCT) (Dose Group 4)

- Adverse events
- Concomitant medications
- PK sample (24 hours after start of infusion). Note: PK sample must be drawn from a different vein than that used for opebacan administration.
- Laboratory evaluations
  - Hematology: CBC with differential and platelet count
- Metabolic assessment (Caloric source documentation including volume and type for all fluids and nutritional sources, intake and output [I&O], and total days of parenteral nutrition [TPN]). Assessments requiring daily totals should be

measured by calendar day (i.e., from midnight to midnight) unless otherwise specified by institutional standard practice.

- Vital signs including pulse, blood pressure (systolic/diastolic), respiratory rate, and temperature taken in the morning (i.e., the first waking set of vital signs). If a subject experiences an SAE, vital signs must be taken more frequently, per standard institutional practice.
- Fever (T_{max})
- LPS and LBP
- Soluble markers that may include, but may not be limited to, sCD14, TNF-RI, IL-1, IL-6, IL-8, IL-10, IL-12, and CRP
- Surface markers at select sites only; may include, but may not be limited to, TLR4 and mCD14
- Oral mucositis score (see Appendix 5)
- Continuation of opebacan dosing. Subjects whose conditioning regimen includes TBI will have their opebacan infusion interrupted for 1-hour TBI treatments twice on Day 0 prior to the HSCT procedure. The HSCT will take place after the opebacan infusion has been restarted for at least 2 hours.

Day 1 (Dose Group 4)

- Adverse events
- Concomitant medications
- PK sample. Note: PK sample must be drawn from a different vein than that used for opebacan administration.
- Metabolic assessment (Intake and output [I&O] and total days of parenteral nutrition [TPN] only). Assessments requiring daily totals should be measured by calendar day (i.e., from midnight to midnight) unless otherwise specified by institutional standard practice.
- Vital signs including pulse, blood pressure (systolic/diastolic), respiratory rate, and temperature taken in the morning (i.e., the first waking set of vital signs). If a subject experiences an SAE, vital signs must be taken more frequently, per standard institutional practice.
- Fever (T_{max})
- Oral mucositis score (see Appendix 5)
- Cardiac serology (CPK-MB, cardiac troponin T, and troponin I)
- Continuation of opebacan dosing

Day 2 (Dose Group 4)

- Adverse events
- Concomitant medications
- Metabolic assessment (Intake and output [I&O] and total days of parenteral nutrition [TPN] only). Assessments requiring daily totals should be measured by calendar day (i.e., from midnight to midnight) unless otherwise specified by institutional standard practice.
- Vital signs including pulse, blood pressure (systolic/diastolic), respiratory rate, and temperature taken in the morning (i.e., the first waking set of vital signs). If a subject experiences an SAE, vital signs must be taken more frequently, per standard institutional practice.
- Fever (T_{max})
- LPS and LBP
- Soluble markers that may include, but may not be limited to, sCD14, TNF-RI, IL-1, IL-6, IL-8, IL-10, IL-12, and CRP
- Surface markers at select sites only; may include, but may not be limited to, TLR4 and mCD14
- Oral mucositis score (see Appendix 5)
- Continuation of opebacan dosing

Day 3 (Dose Group 4)

- Adverse events
- Concomitant medications
- Metabolic assessment (Caloric source documentation including volume and type for all fluids and nutritional sources, intake and output [I&O], and total days of parenteral nutrition [TPN]). Assessments requiring daily totals should be measured by calendar day (i.e., from midnight to midnight) unless otherwise specified by institutional standard practice.
- Vital signs including pulse, blood pressure (systolic/diastolic), respiratory rate, and temperature taken in the morning (i.e., the first waking set of vital signs). If a subject experiences an SAE, vital signs must be taken more frequently, per standard institutional practice.
- Fever (T_{max})
- Oral mucositis score (see Appendix 5)
- Continuation of opebacan dosing

Day 4 (Dose Group 4)

- Adverse events
- Concomitant medications
- Metabolic assessment (Intake and output [I&O] and total days of parenteral nutrition [TPN] only). Assessments requiring daily totals should be measured by calendar day (i.e., from midnight to midnight) unless otherwise specified by institutional standard practice.
- Vital signs including pulse, blood pressure (systolic/diastolic), respiratory rate, and temperature taken in the morning (i.e., the first waking set of vital signs). If a subject experiences an SAE, vital signs must be taken more frequently, per standard institutional practice.
- Fever (T_{max})
- LPS and LBP
- Oral mucositis score (see Appendix 5)
- Cardiac serology (CPK-MB, cardiac troponin T, and troponin I)
- Continuation of opebacan dosing

Day 5 (Dose Group 4)

- Adverse events
- Concomitant medications
- Metabolic assessment (Intake and output [I&O] and total days of parenteral nutrition [TPN] only). Assessments requiring daily totals should be measured by calendar day (i.e., from midnight to midnight) unless otherwise specified by institutional standard practice.
- Vital signs including pulse, blood pressure (systolic/diastolic), respiratory rate, and temperature taken in the morning (i.e., the first waking set of vital signs). If a subject experiences an SAE, vital signs must be taken more frequently, per standard institutional practice.
- Fever (T_{max})
- Oral mucositis score (see Appendix 5)
- Continuation of opebacan dosing

Day 6 (Dose Group 4)

Unless otherwise noted, the following assessments should be performed immediately after the end of the opebacan infusion:

- Adverse events
- Concomitant medications
- PK sample (prior to end of infusion and 15, 30, 60, and 240 minutes after the end of infusion). Note: PK sample must be drawn from a different vein than that used for opebacan administration.
- Laboratory evaluations:
  - Hematology: CBC with differential and platelet count
- Metabolic assessment (Intake and output [I&O] and total days of parenteral nutrition [TPN] only). Assessments requiring daily totals should be measured by calendar day (i.e., from midnight to midnight) unless otherwise specified by institutional standard practice.
- Vital signs including pulse, blood pressure (systolic/diastolic), respiratory rate, and temperature taken in the morning (i.e., the first waking set of vital signs). If a subject experiences an SAE, vital signs must be taken more frequently, per standard institutional practice.
- Fever (T_{max})
- LPS and LBP
- Oral mucositis score (see Appendix 5)
- End of opebacan dosing

Day 7 (Dose Group 4)

- Adverse events
- Concomitant medications
- PK sample (24 hours after the end of infusion). Note: PK sample must be drawn from a different vein than that used for opebacan administration.
- Laboratory evaluations:
  - Chemistries: Sodium, potassium, chloride, bicarbonate, glucose, BUN, creatinine, calcium, phosphorus, magnesium, bilirubin (total, and direct if bilirubin > 2.0 mg/dL), albumin, ALT, AST, LDH, alkaline phosphatase, CPK (with MB fraction), and total protein
  - Coagulation: PT, PTT, fibrinogen, and D-Dimers (or FDP or FSP)
  - Urinalysis: Chemistry and microscopic examination
  - Hematology: CBC with differential and platelet count
- Metabolic assessment (Caloric source documentation including volume and type for all fluids and nutritional sources, intake and output [I&O], and total days of parenteral nutrition [TPN]). Assessments requiring daily totals should be

measured by calendar day (i.e., from midnight to midnight) unless otherwise specified by institutional standard practice.

- Vital signs including pulse, blood pressure (systolic/diastolic), respiratory rate, and temperature taken in the morning (i.e., the first waking set of vital signs). If a subject experiences an SAE, vital signs must be taken more frequently, per standard institutional practice.
- Fever (T_{max})
- Soluble markers that may include, but may not be limited to, sCD14, TNF-RI, IL-1, IL-6, IL-8, IL-10, IL-12, and CRP
- Surface markers at select sites only; may include, but may not be limited to, TLR4 and mCD14
- Oral mucositis score (see Appendix 5)
- LVEF by ECHO or MUGA
- Grading for aGVHD (see Appendix 4)
- Performance score (Karnofsky criteria; see Appendix 3)

Day 8 (Dose Group 4)

- Adverse events
- Concomitant medications
- Metabolic assessment (Intake and output [I&O] and total days of parenteral nutrition [TPN] only). Assessments requiring daily totals should be measured by calendar day (i.e., from midnight to midnight) unless otherwise specified by institutional standard practice.
- Vital signs including pulse, blood pressure (systolic/diastolic), respiratory rate, and temperature taken in the morning (i.e., the first waking set of vital signs). If a subject experiences an SAE, vital signs must be taken more frequently, per standard institutional practice.
- Fever (T_{max})
- LPS and LBP
- Oral mucositis score recorded daily until condition resolves to grade 0 (see Appendix 5)
- Cardiac serology (CPK-MB, cardiac troponin T, and troponin I)

Day 9 (Dose Group 4)

- Adverse events

- Concomitant medications
- Metabolic assessment (Intake and output [I&O] and total days of parenteral nutrition [TPN] only). Assessments requiring daily totals should be measured by calendar day (i.e., from midnight to midnight) unless otherwise specified by institutional standard practice.
- Vital signs including pulse, blood pressure (systolic/diastolic), respiratory rate, and temperature taken in the morning (i.e., the first waking set of vital signs). If a subject experiences an SAE, vital signs must be taken more frequently, per standard institutional practice.
- Fever (T_{max})
- Oral mucositis score recorded daily until condition resolves to grade 0 (see Appendix 5)

Day 10 (Dose Group 4)

- Adverse events
- Concomitant medications
- Laboratory evaluations:
  - Hematology: CBC with differential and platelet count
- Metabolic assessment (Caloric source documentation including volume and type for all fluids and nutritional sources, intake and output [I&O], and total days of parenteral nutrition [TPN]). Assessments requiring daily totals should be measured by calendar day (i.e., from midnight to midnight) unless otherwise specified by institutional standard practice.
- Vital signs including pulse, blood pressure (systolic/diastolic), respiratory rate, and temperature taken in the morning (i.e., the first waking set of vital signs). If a subject experiences an SAE, vital signs must be taken more frequently, per standard institutional practice.
- Fever (T_{max})
- LPS and LBP
- Oral mucositis score recorded daily until condition resolves to grade 0 (see Appendix 5)

Day 11 (Dose Group 4)

- Adverse events
- Concomitant medications

- Metabolic assessment (Intake and output [I&O] and total days of parenteral nutrition [TPN] only). Assessments requiring daily totals should be measured by calendar day (i.e., from midnight to midnight) unless otherwise specified by institutional standard practice.
- Vital signs including pulse, blood pressure (systolic/diastolic), respiratory rate, and temperature taken in the morning (i.e., the first waking set of vital signs). If a subject experiences an SAE, vital signs must be taken more frequently, per standard institutional practice.
- Fever (T_{max})
- Oral mucositis score recorded daily until condition resolves to grade 0 (see Appendix 5)

Day 12 (Dose Group 4)

- Adverse events
- Concomitant medications
- Metabolic assessment (Intake and output [I&O] and total days of parenteral nutrition [TPN] only). Assessments requiring daily totals should be measured by calendar day (i.e., from midnight to midnight) unless otherwise specified by institutional standard practice.
- Vital signs including pulse, blood pressure (systolic/diastolic), respiratory rate, and temperature taken in the morning (i.e., the first waking set of vital signs). If a subject experiences an SAE, vital signs must be taken more frequently, per standard institutional practice.
- Fever (T_{max})
- Oral mucositis score recorded daily until condition resolves to grade 0 (see Appendix 5)

Day 13 (Dose Group 4)

- Adverse events
- Concomitant medications
- Metabolic assessment (Intake and output [I&O] and total days of parenteral nutrition [TPN] only). Assessments requiring daily totals should be measured by calendar day (i.e., from midnight to midnight) unless otherwise specified by institutional standard practice.
- Vital signs including pulse, blood pressure (systolic/diastolic), respiratory rate, and temperature taken in the morning (i.e., the first waking set of vital signs). If a

subject experiences an SAE, vital signs must be taken more frequently, per standard institutional practice.

- Fever (T_{max})
- Oral mucositis score recorded daily until condition resolves to grade 0 (see Appendix 5)

Day 14 (Dose Group 4)

- Adverse events
- Concomitant medications
- Metabolic assessment (Caloric source documentation including volume and type for all fluids and nutritional sources, intake and output [I&O], and total days of parenteral nutrition [TPN]). Assessments requiring daily totals should be measured by calendar day (i.e., from midnight to midnight) unless otherwise specified by institutional standard practice.
- Vital signs including pulse, blood pressure (systolic/diastolic), respiratory rate, and temperature taken in the morning (i.e., the first waking set of vital signs). If a subject experiences an SAE, vital signs must be taken more frequently, per standard institutional practice.
- Laboratory evaluations:
  - Chemistries: Sodium, potassium, chloride, bicarbonate, glucose, BUN, creatinine, calcium, phosphorus, magnesium, bilirubin (total, and direct if bilirubin > 2.0 mg/dL), albumin, ALT, AST, LDH, alkaline phosphatase, and total protein
  - Coagulation: PT, PTT, fibrinogen, and D-Dimers (or FDP or FSP)
  - Urinalysis: Chemistry and microscopic examination
  - Hematology: CBC with differential and platelet count
- LPS and LBP
- LPS-induced TNF and IL-6 at select sites only
- Soluble markers that may include, but may not be limited to, sCD14, TNF-RI, IL-1, IL-6, IL-8, IL-10, IL-12, and CRP
- Surface markers at select sites only; may include, but may not be limited to, TLR4 and mCD14
- Oral mucositis score recorded daily until condition resolves to grade 0 (see Appendix 5)
- Cardiac serology (CPK-MB, cardiac troponin T, and troponin I)
- LVEF by ECHO or MUGA

- Grading for aGVHD (see Appendix 4)
- Performance score (Karnofsky criteria; see Appendix 3)

Day 15 through Day 20 (Dose Group 4)

Unless otherwise noted, the following assessments will be performed daily until the subject is released from the hospital:

- Metabolic assessment (Intake and output [I&O] and total days of parenteral nutrition [TPN]; on Day 17 only, caloric source documentation including volume and type for all fluids and nutritional sources). Assessments requiring daily totals should be measured by calendar day (i.e., from midnight to midnight) unless otherwise specified by institutional standard practice.
- Vital signs including pulse, blood pressure (systolic/diastolic), respiratory rate, and temperature taken in the morning (i.e., the first waking set of vital signs). If a subject experiences an SAE, vital signs must be taken more frequently, per standard institutional practice.
- Oral mucositis score recorded daily until condition resolves to grade 0 (see Appendix 5)

Day 21 (± 3 days) (Dose Group 4)

- Adverse events
- Concomitant medications
- Laboratory evaluations:
  - Chemistries: Sodium, potassium, chloride, bicarbonate, glucose, BUN, creatinine, calcium, phosphorus, magnesium, bilirubin (total, and direct if bilirubin > 2.0 mg/dL), albumin, ALT, AST, LDH, alkaline phosphatase, and total protein
  - Coagulation: PT, PTT, fibrinogen, and D-Dimers (or FDP or FSP)
  - Urinalysis: Chemistry and microscopic examination
  - Hematology: CBC with differential and platelet count
- Metabolic assessment (Caloric source documentation including volume and type for all fluids and nutritional sources, intake and output [I&O], and total days of parenteral nutrition [TPN]). Assessments requiring daily totals should be measured by calendar day (i.e., from midnight to midnight) unless otherwise specified by institutional standard practice.
- Vital signs including pulse, blood pressure (systolic/diastolic), respiratory rate, and temperature taken in the morning (i.e., the first waking set of vital signs). If a

subject experiences an SAE, vital signs must be taken more frequently, per standard institutional practice.

- LPS and LBP
- LPS-induced TNF and IL-6 at select sites only
- Soluble markers that may include, but may not be limited to, sCD14, TNF-RI, IL-1, IL-6, IL-8, IL-10, IL-12, and CRP
- Surface markers at select sites only; may include, but may not be limited to, TLR4 and mCD14
- Oral mucositis score recorded daily until condition resolves to grade 0 (see Appendix 5)
- Cardiac serology (CPK-MB, cardiac troponin T, and troponin I)
- LVEF by ECHO or MUGA
- Grading for aGVHD (see Appendix 4)
- Performance score (Karnofsky criteria; see Appendix 3)

Day 22 through Day 27 (Dose Group 4)

Unless otherwise noted, the following assessments will be performed daily until the subject is released from the hospital:

- Metabolic assessment (Intake and output [I&O] and total days of parenteral nutrition [TPN]; on Day 24 only, caloric source documentation including volume and type for all fluids and nutritional sources). Assessments requiring daily totals should be measured by calendar day (i.e., from midnight to midnight) unless otherwise specified by institutional standard practice.
- Vital signs including pulse, blood pressure (systolic/diastolic), respiratory rate, and temperature taken in the morning (i.e., the first waking set of vital signs). If a subject experiences an SAE, vital signs must be taken more frequently, per standard institutional practice.
- Oral mucositis score recorded daily until condition resolves to grade 0 (see Appendix 5)

Day 28 (± 3 days) (Dose Group 4)

- Adverse events
- Concomitant medications
- Laboratory evaluations:
  - Chemistries: Sodium, potassium, chloride, bicarbonate, glucose, BUN, creatinine, calcium, phosphorus, magnesium, bilirubin (total, and direct if

bilirubin > 2.0 mg/dL), albumin, ALT, AST, LDH, alkaline phosphatase, and total protein

- Coagulation: PT, PTT, fibrinogen, and D-Dimers (or FDP or FSP)
- Urinalysis: Chemistry and microscopic examination
- Hematology: CBC with differential and platelet count

- Metabolic assessment (Caloric source documentation including volume and type for all fluids and nutritional sources, intake and output [I&O], and total days of parenteral nutrition [TPN]). Assessments requiring daily totals should be measured by calendar day (i.e., from midnight to midnight) unless otherwise specified by institutional standard practice.
- Physical exam
- Vital signs including pulse, blood pressure (systolic/diastolic), respiratory rate, and temperature taken in the morning (i.e., the first waking set of vital signs). If a subject experiences an SAE, vital signs must be taken more frequently, per standard institutional practice.
- LPS and LBP
- LPS-induced TNF and IL-6 at select sites only
- Soluble markers that may include, but may not be limited to, sCD14, TNF-RI, IL-1, IL-6, IL-8, IL-10, IL-12, and CRP
- Surface markers at select sites only; may include, but may not be limited to, TLR4 and mCD14
- Oral mucositis score recorded daily until condition resolves to grade 0 (see Appendix 5)
- Cardiac serology (CPK-MB, cardiac troponin T, and troponin I)
- LVEF by ECHO or MUGA
- Grading for aGVHD (see Appendix 4)
- Performance score (Karnofsky criteria; see Appendix 3)
- Anti-opebacan antibody response

Day 29 through Day 34 (Dose Group 4)

The following assessments will be performed daily until the subject is released from the hospital:

- Metabolic assessment (Intake and output [I&O] and total days of parenteral nutrition [TPN] only). Assessments requiring daily totals should be measured by calendar day (i.e., from midnight to midnight) unless otherwise specified by institutional standard practice.

- Vital signs including pulse, blood pressure (systolic/diastolic), respiratory rate, and temperature taken in the morning (i.e., the first waking set of vital signs). If a subject experiences an SAE, vital signs must be taken more frequently, per standard institutional practice.
- Oral mucositis score recorded daily until condition resolves to grade 0 (see Appendix 5)

Day 35 (± 3 days) (Dose Group 4)

The following assessments will be performed only on subjects who are still hospitalized:

- Adverse events
- Concomitant medications
- Laboratory evaluations:
  - Hematology: CBC with differential and platelet count
- Metabolic assessment (Caloric source documentation including volume and type for all fluids and nutritional sources, intake and output [I&O], and total days of parenteral nutrition [TPN]). Assessments requiring daily totals should be measured by calendar day (i.e., from midnight to midnight) unless otherwise specified by institutional standard practice.
- Vital signs including pulse, blood pressure (systolic/diastolic), respiratory rate, and temperature taken in the morning (i.e., the first waking set of vital signs). If a subject experiences an SAE, vital signs must be taken more frequently, per standard institutional practice.
- LPS and LBP
- Soluble markers that may include, but may not be limited to, sCD14, TNF-RI, IL-1, IL-6, IL-8, IL-10, IL-12, and CRP
- Surface markers at select sites only; may include, but may not be limited to, TLR4 and mCD14
- Oral mucositis score recorded daily until condition resolves to grade 0 (see Appendix 5)
- Cardiac serology (CPK-MB, troponin T, and troponin I)
- Performance score (Karnofsky criteria; see Appendix 3)

Day 36 through Day 41 (Dose Group 4)

The following assessments will be performed daily until the subject is released from the hospital:

- Metabolic assessment (Intake and output [I&O] and total days of parenteral nutrition [TPN] only). Assessments requiring daily totals should be measured by calendar day (i.e., from midnight to midnight) unless otherwise specified by institutional standard practice.
- Vital signs including pulse, blood pressure (systolic/diastolic), respiratory rate, and temperature taken in the morning (i.e., the first waking set of vital signs). If a subject experiences an SAE, vital signs must be taken more frequently, per standard institutional practice.
- Oral mucositis score recorded daily until condition resolves to grade 0 (see Appendix 5)

Day 42 (± 3 days) (Dose Group 4)

The following assessments will be performed only on subjects who are still hospitalized:

- Adverse events
- Concomitant medications
- Laboratory evaluations:
  - Hematology: CBC with differential and platelet count
- Metabolic assessment (Caloric source documentation including volume and type for all fluids and nutritional sources, intake and output [I&O], and total days of parenteral nutrition [TPN]). Assessments requiring daily totals should be measured by calendar day (i.e., from midnight to midnight) unless otherwise specified by institutional standard practice.
- Vital signs including pulse, blood pressure (systolic/diastolic), respiratory rate, and temperature taken in the morning (i.e., the first waking set of vital signs). If a subject experiences an SAE, vital signs must be taken more frequently, per standard institutional practice.
- Oral mucositis score recorded daily until condition resolves to grade 0 (see Appendix 5)
- Cardiac serology (CPK-MB, troponin T, and troponin I)
- Grading for aGVHD (see Appendix 4)
- Performance score (Karnofsky criteria; see Appendix 3)

Day 43 through Day 48 (Dose Group 4)

The following assessments will be performed daily until the subject is released from the hospital:

- Metabolic assessment (Intake and output [I&O] and total days of parenteral nutrition [TPN] only). Assessments requiring daily totals should be measured by calendar day (i.e., from midnight to midnight) unless otherwise specified by institutional standard practice.
- Vital signs including pulse, blood pressure (systolic/diastolic), respiratory rate, and temperature taken in the morning (i.e., the first waking set of vital signs). If a subject experiences an SAE, vital signs must be taken more frequently, per standard institutional practice.
- Oral mucositis score recorded daily until condition resolves to grade 0 (see Appendix 5)

Day 49 (± 3 days) (Dose Group 4)

The following assessments will be performed only on subjects who are still hospitalized:

- Adverse events
- Concomitant medications
- Laboratory evaluations:
  - Hematology: CBC with differential and platelet count
- Metabolic assessment (Caloric source documentation including volume and type for all fluids and nutritional sources, intake and output [I&O], and total days of parenteral nutrition [TPN]). Assessments requiring daily totals should be measured by calendar day (i.e., from midnight to midnight) unless otherwise specified by institutional standard practice.
- Vital signs including pulse, blood pressure (systolic/diastolic), respiratory rate, and temperature taken in the morning (i.e., the first waking set of vital signs). If a subject experiences an SAE, vital signs must be taken more frequently, per standard institutional practice.
- Oral mucositis score recorded daily until condition resolves to grade 0 (see Appendix 5)
- Cardiac serology (CPK-MB, troponin T, and troponin I)
- Performance score (Karnofsky criteria; see Appendix 3)

Day 100 (± 14 days) and/or ≥ Grade 2 aGvHD Diagnosis (Dose Group 4)

- Adverse events
- Concomitant medications
- PK sample
- Laboratory evaluations:

  - Chemistries: Sodium, potassium, chloride, bicarbonate, glucose, BUN, creatinine, calcium, phosphorus, magnesium, bilirubin (total, and direct if bilirubin > 2.0 mg/dL), albumin, ALT, AST, LDH, alkaline phosphatase, CPK (with MB fraction), and total protein
  - Coagulation: PT, PTT, fibrinogen, and D-Dimers (or FDP or FSP)
  - Urinalysis: Chemistry and microscopic examination
  - Hematology: CBC with differential and platelet count
- Physical exam
- Vital signs including pulse, blood pressure (systolic/diastolic), respiratory rate, and temperature taken in the morning (i.e., the first waking set of vital signs). If a subject experiences an SAE, vital signs must be taken more frequently, per standard institutional practice.
- LPS and LBP
- LPS-induced TNF and IL-6 at select sites only
- Soluble markers that may include, but may not be limited to, sCD14, TNF-RI, IL-1, IL-6, IL-8, IL-10, IL-12, and CRP
- Surface markers at select sites only; may include, but may not be limited to, TLR4 and mCD14
- LVEF by ECHO or MUGA
- Grading for aGVHD (see Appendix 4)

Hospital Discharge and/or Early Termination (Dose Group 4)

- Adverse Events
- Concomitant medications
- Metabolic assessment (Caloric source documentation including volume and type for all fluids and nutritional sources, intake and output [I&O], and total days of parenteral nutrition [TPN]). Assessments requiring daily totals should be measured by calendar day (i.e., from midnight to midnight) unless otherwise specified by institutional standard practice.
- Physical exam
- Vital signs including pulse, blood pressure (systolic/diastolic), respiratory rate, and temperature taken in the morning (i.e., the first waking set of vital signs). If a subject experiences an SAE, vital signs must be taken more frequently, per standard institutional practice.

4.3.2.3 *Assessments for Treatment and Follow-up Period: Dose Group 5*

Day –1 (Dose Group 5)

- Interim medical history (predose)
- Adverse events (Note: The “signs and symptoms inventory” CRF page must be completed predose and all AEs collected postdose.)
- Review and recording of concomitant medications
- PK sample (predose and 30, 45, 90, and 270 minutes after the start of infusion). Note: PK sample must be drawn from a different vein than that used for opebacan administration.
- Laboratory evaluations (predose):
  - Chemistries: Sodium, potassium, chloride, bicarbonate, glucose, BUN, creatinine, calcium, phosphorus, magnesium, bilirubin (total, and direct if bilirubin > 2.0 mg/dL), albumin, ALT, AST, LDH, alkaline phosphatase, and total protein
  - Coagulation: PT, PTT, fibrinogen, and D-Dimers (or FDP or FSP)
  - Urinalysis: Chemistry and microscopic examination
  - Hematology: CBC with differential and platelet count
- Metabolic assessment (Caloric source documentation including volume and type for all fluids and nutritional sources, intake and output [I&O], and total days of parenteral nutrition [TPN]). Assessments requiring daily totals should be measured by calendar day (i.e., from midnight to midnight) unless otherwise specified by institutional standard practice.
- Vital signs including pulse, blood pressure (systolic/diastolic), respiratory rate, and temperature. Temperature is measured twice: predose (within one hour prior to the start of infusion) and postdose (within four hours after the start of infusion). In addition, body weight must be measured predose to calculate the study drug dose. If a subject experiences an SAE, vital signs must be taken more frequently, per standard institutional practice.
- Fever (T_{max}) measured for two separate periods: predose (within one hour prior to the start of infusion) and postdose (within four hours after the start of infusion).
- LPS and LBP
- LPS-induced TNF and IL-6 (predose) at select sites only
- Soluble markers (predose) that may include, but may not be limited to, sCD14, TNF-RI, IL-1, IL-6, IL-8, IL-10, IL-12, and CRP
- Surface markers (predose) at select sites only; may include, but may not be limited to, TLR4 and mCD14
- Oral mucositis score (predose; see Appendix 5)

- Cardiac serology (CPK-MB, cardiac troponin T, and troponin I) (predose)
- LVEF by ECHO or MUGA (predose)
- Performance score (predose using Karnofsky criteria; see Appendix 3)
- Initiation of opebacan dosing. Subjects whose conditioning regimen includes TBI will have their opebacan infusion interrupted for a 1-hour TBI treatment.

Day 0 (Day of HSCT) (Dose Group 5)

- Adverse events
- Concomitant medications
- PK sample (24 hours after start of infusion). Note: PK sample must be drawn from a different vein than that used for opebacan administration.
- Laboratory evaluations:
  - Hematology: CBC with differential and platelet count
- Metabolic assessment (Caloric source documentation including volume and type for all fluids and nutritional sources, intake and output [I&O], and total days of parenteral nutrition [TPN]). Assessments requiring daily totals should be measured by calendar day (i.e., from midnight to midnight) unless otherwise specified by institutional standard practice.
- Vital signs including pulse, blood pressure (systolic/diastolic), respiratory rate, and temperature taken in the morning (i.e., the first waking set of vital signs). If a subject experiences an SAE, vital signs must be taken more frequently, per standard institutional practice.
- Fever (T_{max})
- LPS and LBP
- Soluble markers that may include, but may not be limited to, sCD14, TNF-RI, IL-1, IL-6, IL-8, IL-10, IL-12, and CRP
- Surface markers at select sites only; may include, but may not be limited to, TLR4 and mCD14
- Oral mucositis score (see Appendix 5)
- Continuation of opebacan dosing. Subjects whose conditioning regimen includes TBI will have their opebacan infusion interrupted for 1-hour TBI treatments twice on Day 0 prior to the HSCT procedure. The HSCT will take place after the opebacan infusion has been restarted for at least 2 hours.

Day 1 (Dose Group 5)

- Adverse events

- Concomitant medications
- PK sample. Note: PK sample must be drawn from a different vein than that used for opebacan administration.
- Metabolic assessment (Intake and output [I&O] and total days of parenteral nutrition [TPN] only). Assessments requiring daily totals should be measured by calendar day (i.e., from midnight to midnight) unless otherwise specified by institutional standard practice.
- Vital signs including pulse, blood pressure (systolic/diastolic), respiratory rate, and temperature taken in the morning (i.e., the first waking set of vital signs). If a subject experiences an SAE, vital signs must be taken more frequently, per standard institutional practice.
- Fever (T_{max})
- Oral mucositis score (see Appendix 5)
- Cardiac serology (CPK-MB, troponin T, and troponin I)
- Continuation of opebacan dosing

Day 2 (Dose Group 5)

- Adverse events
- Concomitant medications
- Metabolic assessment (Intake and output [I&O] and total days of parenteral nutrition [TPN] only). Assessments requiring daily totals should be measured by calendar day (i.e., from midnight to midnight) unless otherwise specified by institutional standard practice.
- Vital signs including pulse, blood pressure (systolic/diastolic), respiratory rate, and temperature taken in the morning (i.e., the first waking set of vital signs). If a subject experiences an SAE, vital signs must be taken more frequently, per standard institutional practice.
- Fever (T_{max})
- LPS and LBP
- Soluble markers that may include, but may not be limited to, sCD14, TNF-RI, IL-1, IL-6, IL-8, IL-10, IL-12, and CRP
- Surface markers at select sites only; may include, but may not be limited to, TLR4 and mCD14
- Oral mucositis score (see Appendix 5)
- Continuation of opebacan dosing

Day 3 (Dose Group 5)

- Adverse events
- Concomitant medications
- Metabolic assessment (Caloric source documentation including volume and type for all fluids and nutritional sources, intake and output [I&O], and total days of parenteral nutrition [TPN]). Assessments requiring daily totals should be measured by calendar day (i.e., from midnight to midnight) unless otherwise specified by institutional standard practice.
- Vital signs including pulse, blood pressure (systolic/diastolic), respiratory rate, and temperature taken in the morning (i.e., the first waking set of vital signs). If a subject experiences an SAE, vital signs must be taken more frequently, per standard institutional practice.
- Fever (T_{max})
- Oral mucositis score (see Appendix 5)
- Continuation of opebacan dosing

Day 4 (Dose Group 5)

- Adverse events
- Concomitant medications
- Metabolic assessment (Intake and output [I&O] and total days of parenteral nutrition [TPN] only). Assessments requiring daily totals should be measured by calendar day (i.e., from midnight to midnight) unless otherwise specified by institutional standard practice.
- Vital signs including pulse, blood pressure (systolic/diastolic), respiratory rate, and temperature taken in the morning (i.e., the first waking set of vital signs). If a subject experiences an SAE, vital signs must be taken more frequently, per standard institutional practice.
- Fever (T_{max})
- LPS and LBP
- Oral mucositis score (see Appendix 5)
- Cardiac serology (CPK-MB, troponin T, and troponin I)
- Continuation of opebacan dosing

Day 5 (Dose Group 5)

- Adverse events

- Concomitant medications
- Metabolic assessment (Intake and output [I&O] and total days of parenteral nutrition [TPN] only). Assessments requiring daily totals should be measured by calendar day (i.e., from midnight to midnight) unless otherwise specified by institutional standard practice.
- Vital signs including pulse, blood pressure (systolic/diastolic), respiratory rate, and temperature taken in the morning (i.e., the first waking set of vital signs). If a subject experiences an SAE, vital signs must be taken more frequently, per standard institutional practice.
- Fever (T_{max})
- Oral mucositis score (see Appendix 5)
- Continuation of opebacan dosing

Day 6 (Dose Group 5)

- Adverse events
- Concomitant medications
- PK sample. Note: PK sample must be drawn from a different vein than that used for opebacan administration.
- Metabolic assessment (Intake and output [I&O] and total days of parenteral nutrition [TPN] only). Assessments requiring daily totals should be measured by calendar day (i.e., from midnight to midnight) unless otherwise specified by institutional standard practice.
- Vital signs including pulse, blood pressure (systolic/diastolic), respiratory rate, and temperature taken in the morning (i.e., the first waking set of vital signs). If a subject experiences an SAE, vital signs must be taken more frequently, per standard institutional practice.
- Fever (T_{max})
- LPS and LBP
- Oral mucositis score (see Appendix 5)
- Continuation of opebacan dosing

Day 7 (Dose Group 5)

- Adverse events
- Concomitant medications
- Laboratory evaluations:

  - Hematology: CBC with differential and platelet count

- Metabolic assessment (Caloric source documentation including volume and type for all fluids and nutritional sources, intake and output [I&O], and total days of parenteral nutrition [TPN]). Assessments requiring daily totals should be measured by calendar day (i.e., from midnight to midnight) unless otherwise specified by institutional standard practice.
- Vital signs including pulse, blood pressure (systolic/diastolic), respiratory rate, and temperature taken in the morning (i.e., the first waking set of vital signs). If a subject experiences an SAE, vital signs must be taken more frequently, per standard institutional practice.
- Fever (T_{max})
- Soluble markers that may include, but may not be limited to, sCD14, TNF-RI, IL-1, IL-6, IL-8, IL-10, IL-12, and CRP
- Surface markers at select sites only; may include, but may not be limited to, TLR4 and mCD14
- Oral mucositis score (see Appendix 5)
- LVEF by ECHO or MUGA
- Grading for aGVHD (see Appendix 4)
- Performance score (Karnofsky criteria; see Appendix 3)
- Continuation of opebacan dosing

Day 8 (Dose Group 5)

- Adverse events
- Concomitant medications
- Laboratory evaluations (predose)
  - Chemistries: Sodium, potassium, chloride, bicarbonate, glucose, BUN, creatinine, calcium, phosphorus, magnesium, bilirubin (total, and direct if bilirubin > 2.0 mg/dL), albumin, ALT, AST, LDH, alkaline phosphatase, and total protein
  - Coagulation: PT, PTT, fibrinogen, and D-Dimers (or FDP or FSP)
  - Urinalysis: Chemistry and microscopic examination
- Metabolic assessment (Intake and output [I&O] and total days of parenteral nutrition [TPN] only). Assessments requiring daily totals should be measured by calendar day (i.e., from midnight to midnight) unless otherwise specified by institutional standard practice.

- Vital signs including pulse, blood pressure (systolic/diastolic), respiratory rate, and temperature taken in the morning (i.e., the first waking set of vital signs). If a subject experiences an SAE, vital signs must be taken more frequently, per standard institutional practice.
- Fever (T_{max})
- LPS and LBP
- Oral mucositis score recorded daily until condition resolves to grade 0 (see Appendix 5)
- Cardiac serology (CPK-MB, cardiac troponin T, and troponin I)
- Continuation of opebacan dosing

Day 9 (Dose Group 5)

- Adverse events
- Concomitant medications
- Metabolic assessment (Intake and output [I&O] and total days of parenteral nutrition [TPN] only). Assessments requiring daily totals should be measured by calendar day (i.e., from midnight to midnight) unless otherwise specified by institutional standard practice.
- Vital signs including pulse, blood pressure (systolic/diastolic), respiratory rate, and temperature taken in the morning (i.e., the first waking set of vital signs). If a subject experiences an SAE, vital signs must be taken more frequently, per standard institutional practice.
- Fever (T_{max})
- Oral mucositis score recorded daily until condition resolves to grade 0 (see Appendix 5)
- Continuation of opebacan dosing

Day 10 (Dose Group 5)

- Adverse events
- Concomitant medications
- PK sample. Note: PK sample must be drawn from a different vein than that used for opebacan administration.
- Laboratory evaluations:
  - Hematology: CBC with differential and platelet count

- Metabolic assessment (Caloric source documentation including volume and type for all fluids and nutritional sources, intake and output [I&O], and total days of parenteral nutrition [TPN]). Assessments requiring daily totals should be measured by calendar day (i.e., from midnight to midnight) unless otherwise specified by institutional standard practice.
- Vital signs including pulse, blood pressure (systolic/diastolic), respiratory rate, and temperature taken in the morning (i.e., the first waking set of vital signs). If a subject experiences an SAE, vital signs must be taken more frequently, per standard institutional practice.
- Fever (T_{max})
- LPS and LBP
- Oral mucositis score recorded daily until condition resolves to grade 0 (see Appendix 5)
- Continuation of opebacan dosing

Day 11 (Dose Group 5)

- Adverse events
- Concomitant medications
- Metabolic assessment (Intake and output [I&O] and total days of parenteral nutrition [TPN] only). Assessments requiring daily totals should be measured by calendar day (i.e., from midnight to midnight) unless otherwise specified by institutional standard practice.
- Vital signs including pulse, blood pressure (systolic/diastolic), respiratory rate, and temperature taken in the morning (i.e., the first waking set of vital signs). If a subject experiences an SAE, vital signs must be taken more frequently, per standard institutional practice.
- Fever (T_{max})
- Oral mucositis score recorded daily until condition resolves to grade 0 (see Appendix 5)
- Continuation of opebacan dosing

Day 12 (Dose Group 5)

- Adverse events
- Concomitant medications
- Metabolic assessment (Intake and output [I&O] and total days of parenteral nutrition [TPN] only). Assessments requiring daily totals should be measured by

calendar day (i.e., from midnight to midnight) unless otherwise specified by institutional standard practice.

- Vital signs including pulse, blood pressure (systolic/diastolic), respiratory rate, and temperature taken in the morning (i.e., the first waking set of vital signs). If a subject experiences an SAE, vital signs must be taken more frequently, per standard institutional practice.
- Fever (T_{max})
- Oral mucositis score recorded daily until condition resolves to grade 0 (see Appendix 5)
- Continuation of opebacan dosing

Day 13 (Dose Group 5)

Unless otherwise noted, the following assessments should be performed immediately after the end of the opebacan infusion:

- Adverse events
- Concomitant medications
- PK sample (prior to end of infusion and 15, 30, 60, and 240 minutes after the end of infusion). Note: PK sample must be drawn from a different vein than that used for opebacan administration.
- Laboratory evaluations
  - Hematology: CBC with differential and platelet count
- Metabolic assessment (Intake and output [I&O] and total days of parenteral nutrition [TPN] only). Assessments requiring daily totals should be measured by calendar day (i.e., from midnight to midnight) unless otherwise specified by institutional standard practice.
- Vital signs including pulse, blood pressure (systolic/diastolic), respiratory rate, and temperature taken in the morning (i.e., the first waking set of vital signs). If a subject experiences an SAE, vital signs must be taken more frequently, per standard institutional practice.
- Fever (T_{max})
- Oral mucositis score recorded daily until condition resolves to grade 0 (see Appendix 5)
- End of opebacan dosing

Day 14 (Dose Group 5)

- Adverse events

- Concomitant medications
- PK sample (24 hours after the end of infusion). Note: PK sample must be drawn from a different vein than that used for opebacan administration.
- Metabolic assessment (Caloric source documentation including volume and type for all fluids and nutritional sources, intake and output [I&O], and total days of parenteral nutrition [TPN]). Assessments requiring daily totals should be measured by calendar day (i.e., from midnight to midnight) unless otherwise specified by institutional standard practice.
- Vital signs including pulse, blood pressure (systolic/diastolic), respiratory rate, and temperature taken in the morning (i.e., the first waking set of vital signs). If a subject experiences an SAE, vital signs must be taken more frequently, per standard institutional practice.
- Laboratory evaluations:
  - Chemistries: Sodium, potassium, chloride, bicarbonate, glucose, BUN, creatinine, calcium, phosphorus, magnesium, bilirubin (total, and direct if bilirubin > 2.0 mg/dL), albumin, ALT, AST, LDH, alkaline phosphatase, and total protein
  - Coagulation: PT, PTT, fibrinogen, and D-Dimers (or FDP or FSP)
  - Urinalysis: Chemistry and microscopic examination
  - Hematology: CBC with differential and platelet count
- LPS and LBP
- LPS-induced TNF and IL-6 at select sites only
- Soluble markers that may include, but may not be limited to, sCD14, TNF-RI, IL-1, IL-6, IL-8, IL-10, IL-12, and CRP
- Surface markers at select sites only; may include, but may not be limited to, TLR4 and mCD14
- Oral mucositis score recorded daily until condition resolves to grade 0 (see Appendix 5)
- Cardiac serology (CPK-MB, cardiac troponin T, and troponin I)
- LVEF by ECHO or MUGA
- Grading for aGVHD (see Appendix 4)
- Performance score (Karnofsky criteria; see Appendix 3)

Day 15 through Day 20 (Dose Group 5)

Unless otherwise noted, the following assessments will be performed daily until the subject is released from the hospital:

- Metabolic assessment (Intake and output [I&O] and total days of parenteral nutrition [TPN]; on Day 17 only, caloric source documentation including volume and type for all fluids and nutritional sources). Assessments requiring daily totals should be measured by calendar day (i.e., from midnight to midnight) unless otherwise specified by institutional standard practice.
- Vital signs including pulse, blood pressure (systolic/diastolic), respiratory rate, and temperature taken in the morning (i.e., the first waking set of vital signs). If a subject experiences an SAE, vital signs must be taken more frequently, per standard institutional practice.
- Oral mucositis score recorded daily until condition resolves to grade 0 (see Appendix 5)

Day 21 (± 3 days) (Dose Group 5)

- Adverse events
- Concomitant medications
- Laboratory evaluations:
  - Chemistries: Sodium, potassium, chloride, bicarbonate, glucose, BUN, creatinine, calcium, phosphorus, magnesium, bilirubin (total, and direct if bilirubin > 2.0 mg/dL), albumin, ALT, AST, LDH, alkaline phosphatase, and total protein
  - Coagulation: PT, PTT, fibrinogen, and D-Dimers (or FDP or FSP)
  - Urinalysis: Chemistry and microscopic examination
  - Hematology: CBC with differential and platelet count
- Metabolic assessment (Caloric source documentation including volume and type for all fluids and nutritional sources, intake and output [I&O], and total days of parenteral nutrition [TPN]). Assessments requiring daily totals should be measured by calendar day (i.e., from midnight to midnight) unless otherwise specified by institutional standard practice.
- Vital signs including pulse, blood pressure (systolic/diastolic), respiratory rate, and temperature taken in the morning (i.e., the first waking set of vital signs). If a subject experiences an SAE, vital signs must be taken more frequently, per standard institutional practice.
- LPS and LBP
- LPS-induced TNF and IL-6 at select sites only
- Soluble markers that may include, but may not be limited to, sCD14, TNF-RI, IL-1, IL-6, IL-8, IL-10, IL-12, and CRP
- Surface markers at select sites only; may include, but may not be limited to, TLR4 and mCD14

- Oral mucositis score recorded daily until condition resolves to grade 0 (see Appendix 5)
- Cardiac serology (CPK-MB, cardiac troponin T, and troponin I)
- LVEF by ECHO or MUGA
- Grading for aGVHD (see Appendix 4)
- Performance score (Karnofsky criteria; see Appendix 3)

Day 22 through Day 27 (Dose Group 5)

Unless otherwise noted, the following assessments will be performed daily until the subject is released from the hospital:

- Metabolic assessment (Intake and output [I&O] and total days of parenteral nutrition [TPN]; on Day 24 only, caloric source documentation including volume and type for all fluids and nutritional sources). Assessments requiring daily totals should be measured by calendar day (i.e., from midnight to midnight) unless otherwise specified by institutional standard practice.
- Vital signs including pulse, blood pressure (systolic/diastolic), respiratory rate, and temperature taken in the morning (i.e., the first waking set of vital signs). If a subject experiences an SAE, vital signs must be taken more frequently, per standard institutional practice.
- Oral mucositis score recorded daily until condition resolves to grade 0 (see Appendix 5)

Day 28 (± 3 days) (Dose Group 5)

- Adverse events
- Concomitant medications
- Laboratory evaluations:
    - Chemistries: Sodium, potassium, chloride, bicarbonate, glucose, BUN, creatinine, calcium, phosphorus, magnesium, bilirubin (total, and direct if bilirubin > 2.0 mg/dL), albumin, ALT, AST, LDH, alkaline phosphatase, and total protein
    - Coagulation: PT, PTT, fibrinogen, and D-Dimers (or FDP or FSP)
    - Urinalysis: Chemistry and microscopic examination
    - Hematology: CBC with differential and platelet count
- Metabolic assessment (Caloric source documentation including volume and type for all fluids and nutritional sources, intake and output [I&O], and total days of parenteral nutrition [TPN]). Assessments requiring daily totals should be

measured by calendar day (i.e., from midnight to midnight) unless otherwise specified by institutional standard practice.

- Physical exam
- Vital signs including pulse, blood pressure (systolic/diastolic), respiratory rate, and temperature taken in the morning (i.e., the first waking set of vital signs). If a subject experiences an SAE, vital signs must be taken more frequently, per standard institutional practice.
- LPS and LBP
- LPS-induced TNF and IL-6 at select sites only
- Soluble markers that may include, but may not be limited to, sCD14, TNF-RI, IL-1, IL-6, IL-8, IL-10, IL-12, and CRP
- Surface markers at select sites only; may include, but may not be limited to, TLR4 and mCD14
- Oral mucositis score recorded daily until condition resolves to grade 0 (see Appendix 5)
- Cardiac serology (CPK-MB, cardiac troponin T, and troponin I)
- LVEF by ECHO or MUGA
- Grading for aGVHD (see Appendix 4)
- Performance score (Karnofsky criteria; see Appendix 3)
- Anti-opebacan antibody response

Day 29 through Day 34 (Dose Group 5)

The following assessments will be performed daily until the subject is released from the hospital:

- Metabolic assessment (Intake and output [I&O] and total days of parenteral nutrition [TPN] only). Assessments requiring daily totals should be measured by calendar day (i.e., from midnight to midnight) unless otherwise specified by institutional standard practice.
- Vital signs including pulse, blood pressure (systolic/diastolic), respiratory rate, and temperature taken in the morning (i.e., the first waking set of vital signs). If a subject experiences an SAE, vital signs must be taken more frequently, per standard institutional practice.
- Oral mucositis score recorded daily until condition resolves to grade 0 (see Appendix 5)

Day 35 (± 3 days) (Dose Group 5)

The following assessments will be performed only on subjects who are still hospitalized:

- Adverse events
- Concomitant medications
- Laboratory evaluations:
  - Hematology: CBC with differential and platelet count
- Metabolic assessment (Caloric source documentation including volume and type for all fluids and nutritional sources, intake and output [I&O], and total days of parenteral nutrition [TPN]). Assessments requiring daily totals should be measured by calendar day (i.e., from midnight to midnight) unless otherwise specified by institutional standard practice.
- Vital signs including pulse, blood pressure (systolic/diastolic), respiratory rate, and temperature taken in the morning (i.e., the first waking set of vital signs). If a subject experiences an SAE, vital signs must be taken more frequently, per standard institutional practice.
- LPS and LBP
- Soluble markers that may include, but may not be limited to, sCD14, TNF-RI, IL-1, IL-6, IL-8, IL-10, IL-12, and CRP
- Surface markers at select sites only; may include, but may not be limited to, TLR4 and mCD14
- Oral mucositis score recorded daily until condition resolves to grade 0 (see Appendix 5)
- Cardiac serology (CPK-MB, troponin T, and troponin I)
- Performance score (Karnofsky criteria; see Appendix 3)

Day 36 through Day 41 (Dose Group 5)

The following assessments will be performed daily until the subject is released from the hospital:

- Metabolic assessment (Intake and output [I&O] and total days of parenteral nutrition [TPN] only). Assessments requiring daily totals should be measured by calendar day (i.e., from midnight to midnight) unless otherwise specified by institutional standard practice.
- Vital signs including pulse, blood pressure (systolic/diastolic), respiratory rate, and temperature taken in the morning (i.e., the first waking set of vital signs). If a subject experiences an SAE, vital signs must be taken more frequently, per standard institutional practice.

- Oral mucositis score recorded daily until condition resolves to grade 0 (see Appendix 5)

Day 42 (± 3 days) (Dose Group 5)

The following assessments will be performed only on subjects who are still hospitalized:

- Adverse events
- Concomitant medications
- Laboratory evaluations:
  - Hematology: CBC with differential and platelet count
- Metabolic assessment (Caloric source documentation including volume and type for all fluids and nutritional sources, intake and output [I&O], and total days of parenteral nutrition [TPN]). Assessments requiring daily totals should be measured by calendar day (i.e., from midnight to midnight) unless otherwise specified by institutional standard practice.
- Vital signs including pulse, blood pressure (systolic/diastolic), respiratory rate, and temperature taken in the morning (i.e., the first waking set of vital signs). If a subject experiences an SAE, vital signs must be taken more frequently, per standard institutional practice.
- Oral mucositis score recorded daily until condition resolves to grade 0 (see Appendix 5)
- Cardiac serology (CPK-MB, troponin T, and troponin I)
- Grading for aGVHD (see Appendix 4)
- Performance score (Karnofsky criteria; see Appendix 3)

Day 43 through Day 48 (Dose Group 5)

The following assessments will be performed daily until the subject is released from the hospital:

- Metabolic assessment (Intake and output [I&O] and total days of parenteral nutrition [TPN] only). Assessments requiring daily totals should be measured by calendar day (i.e., from midnight to midnight) unless otherwise specified by institutional standard practice.
- Vital signs including pulse, blood pressure (systolic/diastolic), respiratory rate, and temperature taken in the morning (i.e., the first waking set of vital signs). If a subject experiences an SAE, vital signs must be taken more frequently, per standard institutional practice.

- Oral mucositis score recorded daily until condition resolves to grade 0 (see Appendix 5)

Day 49 (± 3 days) (Dose Group 5)

The following assessments will be performed only on subjects who are still hospitalized:

- Adverse events
- Concomitant medications
- Laboratory evaluations:
  - Hematology: CBC with differential and platelet count
- Metabolic assessment (Caloric source documentation including volume and type for all fluids and nutritional sources, intake and output [I&O], and total days of parenteral nutrition [TPN]). Assessments requiring daily totals should be measured by calendar day (i.e., from midnight to midnight) unless otherwise specified by institutional standard practice.
- Vital signs including pulse, blood pressure (systolic/diastolic), respiratory rate, and temperature taken in the morning (i.e., the first waking set of vital signs). If a subject experiences an SAE, vital signs must be taken more frequently, per standard institutional practice.
- Oral mucositis score recorded daily until condition resolves to grade 0 (see Appendix 5)
- Cardiac serology (CPK-MB, troponin T, and troponin I)
- Performance score (Karnofsky criteria; see Appendix 3)

Day 100 (± 14 days) and/or ≥ Grade 2 aGvHD Diagnosis (Dose Group 5)

- Adverse events
- Concomitant medications
- PK sample
- Laboratory evaluations:
  - Chemistries: Sodium, potassium, chloride, bicarbonate, glucose, BUN, creatinine, calcium, phosphorus, magnesium, bilirubin (total, and direct if bilirubin > 2.0 mg/dL), albumin, ALT, AST, LDH, alkaline phosphatase, CPK (with MB fraction), and total protein
  - Coagulation: PT, PTT, fibrinogen, and D-Dimers (or FDP or FSP)
  - Urinalysis: Chemistry and microscopic examination
  - Hematology: CBC with differential and platelet count

- Physical exam
- Vital signs including pulse, blood pressure (systolic/diastolic), respiratory rate, and temperature taken in the morning (i.e., the first waking set of vital signs). If a subject experiences an SAE, vital signs must be taken more frequently, per standard institutional practice.
- LPS and LBP
- LPS-induced TNF and IL-6 at select sites only
- Soluble markers that may include, but may not be limited to, sCD14, TNF-RI, IL-1, IL-6, IL-8, IL-10, IL-12, and CRP
- Surface markers at select sites only; may include, but may not be limited to, TLR4 and mCD14
- LVEF by ECHO or MUGA
- Grading for aGVHD (see Appendix 4)

Hospital Discharge and/or Early Termination (Dose Group 5)

- Adverse Events
- Concomitant medications
- Metabolic assessment (Caloric source documentation including volume and type for all fluids and nutritional sources, intake and output [I&O], and total days of parenteral nutrition [TPN]). Assessments requiring daily totals should be measured by calendar day (i.e., from midnight to midnight) unless otherwise specified by institutional standard practice.
- Physical exam
- Vital signs including pulse, blood pressure (systolic/diastolic), respiratory rate, and temperature taken in the morning (i.e., the first waking set of vital signs). If a subject experiences an SAE, vital signs must be taken more frequently, per standard institutional practice.

4.4 Donor Assessments

Before any evaluations or measurements are performed on the donor, the donor or legal guardian must be fully informed and sign a XOMA/IRB-approved informed consent form for participation in this study. If a donor declines to consent to study participation, the subject who receives that donor's stem cells will continue to be included in the study. The following assessments must be performed and samples collected on donors prior to harvest of donor cells:

- Signed XOMA/IRB-approved informed consent
- Demographic information (sex, age, race/ethnicity)

- Medical history, including virology and serology status
- DNA testing (SNPs for markers such as TLR and TNF) at select sites only
- PK sample for measuring level of endogenous BPI. Note: PK sample must be drawn from a different vein than that used for opebacan administration.
- LPS and LBP
- LPS-induced TNF and IL-6 at select sites only
- Anti-opebacan antibody response

4.5 Discontinuation

4.5.1 Subject Discontinuation

Subjects may withdraw or be withdrawn from the study at any time. Every effort should be made to obtain complete follow-up information on subjects who receive any amount of opebacan and discontinue from the study prematurely. The reason(s) for a subject's discontinuation from the study must be clearly documented in the subject's medical records and on the CRF.

A subject who experiences DLT at any dose level will discontinue study drug. After receiving treatment for the toxicity (if necessary), the subject will be closely monitored until the toxicity is resolved to no worse than grade 2 (grade 1 if reported toxicity is initially grade 2) severity or is stabilized, at which point he/she will be removed from the study.

In the event that a subject discontinues participation in the study prior to receiving opebacan, the subject will be replaced. No follow-up or additional procedures will be performed on subjects discontinuing prior to receiving opebacan.

Subjects will also be replaced if they do not receive at least 80% of their scheduled dose of opebacan and/or do not complete assessments for at least 7 days following the end of their opebacan infusion.

If the subject discontinues participation in the study after receiving any amount of opebacan, every effort should be made to complete as many subsequent protocol evaluations as possible.

4.5.2 Study Discontinuation

XOMA has the right to terminate this study at any time for any reason. Reasons for terminating the study may include the following:

- The incidence or severity of AEs indicates a potential health hazard to subjects.
- Subject enrollment is unsatisfactory.

5.0 ASSESSMENT OF SAFETY

All regulations stated in 21 CFR Parts 50, 56 and 312 and recommendations outlined in the ICH Guidelines for Good Clinical Practice will be adhered to throughout this trial. The safety of the drug will be assessed by multiple subject assessments of vital signs, physical exams, clinical tests and laboratory evaluations. Concomitant medications and adverse events will be monitored and tracked.

Following a comprehensive baseline evaluation of each subject, subject safety will be monitored by evaluating all adverse events (AEs) that occur following treatment with study drug and by performing periodic physical examinations and laboratory tests (hematology, chemistry, and urinalysis).

Once any evidence of unexpected clinical and/or laboratory toxicity is noticed, the Investigator should take appropriate and prompt remedial measures while trying to elucidate the etiology of the condition. The subjects will be followed until the condition resolves or becomes chronic or stable. The Investigator should document and report the event to the Sponsor.

Safety will be monitored and tracked for every subject given any amount of study drug.

5.1 Adverse Events

The occurrence of an adverse event will be determined based on observed or volunteered signs and symptoms, as well as changes in the subject's physical examination and laboratory results.

Adverse events that occur during the study will be recorded on the appropriate AE pages of the CRF. On Day −1, a "signs and symptoms inventory" CRF page will be completed prior to initial dosing with opebacan.

An AE is defined as any untoward medical occurrence (e.g., sign, symptom, disease, syndrome, intercurrent illness, abnormal laboratory finding) that emerges or worsens relative to pretreatment baseline during the study, regardless of the suspected cause. Untoward medical events that occur from the time the subject signs the informed consent form to the time the administration of the drug starts are not considered adverse events, and should be recorded under medical history.

Acute infusional adverse events may include headache, fever, chills, myalgia or nausea experienced 12 to 48 hours following initiation of IV administration of study drug.

All AEs will be evaluated by the Investigator for their seriousness, severity, relationship to study drug, and outcome.

An AE occurring **at any dose** (including overdose) should be classified as **SERIOUS** if:

- It resulted in death (i.e., the AE caused or led to death).
- It was life threatening (i.e., the AE placed the subject at immediate risk of death; an AE should not be classified as life-threatening if it hypothetically might have caused death if it were more severe).
- It required or prolonged inpatient hospitalization (i.e., the AE required at least a 24-hour inpatient hospitalization or prolonged a hospitalization beyond the expected length of stay; hospitalizations for elective medical/surgical procedures, scheduled treatments, or routine check-ups are not SAEs by this criterion).
- It was disabling (i.e., the AE resulted in a substantial disruption of the subject's ability to carry out normal life functions).
- It resulted in a congenital anomaly/birth defect (i.e., an adverse outcome in a child or fetus of a subject exposed to the molecule or study drug prior to conception or during pregnancy).
- It does not meet any of the above criteria for a serious AE but may jeopardize the subject or may require medical or surgical intervention to prevent one of the outcomes listed above.

Note: As stated in Section 5.2, certain transplant-related experiences will not be reported as serious adverse events unless the event is life threatening or fatal.

All adverse events will be graded for severity according to the NCI Common Terminology Criteria for Adverse Events (CTCAE), version 3.0 (available on line at http://ctep.cancer.gov/reporting/ctc.html).

Using the following criteria, Investigators also need to assess whether there is a reasonable possibility that study drug caused or contributed to the AE:

Yes (possibly, probably, or definitely related):

- There is a clinically plausible time sequence between onset of the AE and study drug administration

  AND/OR
- There is a biologically plausible mechanism for study drug causing or contributing to the AE

No (unlikely, unrelated):

- Another cause of the AE is most plausible

  AND/OR
- A clinically plausible temporal sequence is inconsistent with the onset of the AE and study drug administration

AND/OR

- A causal relationship is considered biologically implausible

The Principal Investigator will be asked to list any other suspected causes.

5.2 Baseline Medical Conditions and Underlying Disease

Unchanged chronic conditions or those related to the underlying disease which are consistent with the disease’s natural progression are not AEs and are not to be recorded on the AE page of the CRF unless there is an exacerbation. These medical conditions are to be adequately documented on the medical history and/or signs and symptoms inventory page of the CRF. However, medical conditions present at baseline (Day −1) that worsen in intensity or frequency during the study must be reported and recorded as AEs.

All HSCT transplant patients are critically ill at the time of their transplant. The most common severe adverse events in this population are cardiopulmonary arrest, progressive respiratory failure requiring intubation, life-threatening opportunistic infection, severe veno-occlusive disease (VOD), and graft failure. It is expected that deaths will occur while this trial is ongoing because of the mortality associated with both allogeneic HSCT and the subjects’ underlying diseases. The rates of HSCT-related morbidity and mortality are well-established to be significant. All serious adverse events, including those associated with a fatality, will be carefully and thoroughly evaluated by the Principal Investigator both in the context of the subject’s specific history and in the context of the overall protocol. If, after any of these events, the DSMB, in consultation with the Investigators, determines that the continuation of the trial is no longer in the best interests of the subjects, the trial will be suspended immediately.

HSCT recipients are likely to have the following adverse events and laboratory abnormalities that are related to their transplant conditioning regimen or the transplant procedure: nausea, anorexia, vomiting, gastrointestinal injury including severe mucositis, epigastric discomfort and increased stool output during and in the two to three weeks after completion of conditioning, fever and neutropenia, opportunistic infection, VOD, fluid and electrolyte imbalance including hypomagnesemia, hypokalemia, hyponatremia, hypocalcemia, hypophosphatemia, blood sugar and lipid abnormalities, renal insufficiency, transaminitis, GvHD and hemorrhagic cystitis. These transplant-related experiences will not be reported as serious adverse events unless the event is life threatening or fatal. However, they will be collected as AEs and noted as not related to the use of study drug where appropriate. On Day −1, a “signs and symptoms inventory” CRF page will be completed prior to initial dosing with opebacan.

5.3 Laboratory Tests/Other Evaluations

This study involves various procedures and tests performed repeatedly throughout the study. If a subject has a clinically significant abnormal laboratory test value that is not an expected

result of study drug administration or was not present at baseline, the test will be repeated and the subject will be followed until the test value has returned to the normal range or the Investigator has determined that the abnormality is chronic or stable.

The Investigators are required to evaluate all abnormal laboratory results for their clinical significance. An isolated abnormal laboratory result in the absence of any associated clinical finding may not be considered an AE. An abnormal laboratory result will be considered clinically significant and recorded as an AE when it is part of a clinical abnormality requiring specific medical intervention or follow-up.

A complete physical examination will be performed at prescreening, Day 28, Day 100 and hospital discharge. Vital signs will be recorded at specified time points throughout the study. Vital sign measurements consist of blood pressure, pulse rate, body temperature and body weight.

For women of child-bearing potential, a urine or serum pregnancy test will be performed at screening.

5.4 Procedures for Eliciting, Recording, and Reporting Adverse Events

Adverse events may be directly observed or elicited by the Investigator using open-ended or directed questions, and/or volunteered by the subjects.

5.4.1 Recording Adverse Events

To improve the quality and precision of acquired AE data, Investigators should observe the following guidelines:

- Whenever possible, use recognized medical terms when recording AEs on the AE pages of the CRF. Do not use colloquialisms and/or abbreviations.
- If known, record the diagnosis (i.e., disease or syndrome) rather than component signs and symptoms on the AE pages of the CRF (e.g., record congestive heart failure rather than dyspnea, rales, and cyanosis). However, signs and symptoms that are considered unrelated to an encountered syndrome or disease should be recorded as individual AEs on the CRF (e.g., if congestive heart failure has not been documented, each symptom in the above example would be recorded as a separate AE).
- Adverse events occurring secondary to other events (e.g., sequelae) should be identified by the primary cause. A “primary” AE, if clearly identifiable, generally represents the most accurate clinical term to record on AE pages of the CRF. Events occurring secondary to the primary event should be described in the narrative description of the case. For example:

Orthostatic hypotension	→	Fainting and fall to floor	→	Head trauma	→	Neck pain

The primary AE in this example is orthostatic hypotension.

5.4.2 Adverse Events Requiring Expedited Reporting

All SAEs will be carefully monitored throughout the course of the study.

All SAEs, regardless of relationship to study drug, must be reported by the Investigator to XOMA or its designee within 24 hours of being notified of the event. The Investigator should contact XOMA or its designee by telephone, followed by a faxed initial SAE form. Additional written follow-up information, including a full description of the event and any sequelae, must follow as soon as available.

Telephone Report:

Alan Solinger, M.D.<br>Medical Monitor	Office:	(800) 858-2783<br>(510) 204-7448

Concurrent SAE Form:

Sally Bernard	Fax:<br>Phone:	(510) 548-8615<br>(510) 204-7467

For initial SAE reports, record all case details that can be gathered within 24 hours on the SAE Form. The completed SAE form should be faxed immediately upon completion. Relevant follow-up information should be submitted as soon as possible. All SAEs should also be reported to the IRB within the time frame determined by the institution at which the investigation is being carried out.

5.4.3 Special Reporting Situations

Fatal or life-threatening events thought to be caused by study drug should be immediately reported to the Medical Monitor by telephone.

5.4.3.1 Death

Death is an outcome of an event. The **event** that resulted in the death should be recorded and reported on the AE CRF, SAE Form, and Death Report Form.

5.4.3.2 *Hospitalizations for Surgical or Diagnostic Procedures*

The **illness** leading to the surgical or diagnostic procedure should be recorded as the SAE, not the procedure itself. The procedure should be captured in the case narrative as part of the action taken in response to the illness.

5.5 Type and Duration of Follow-up After Adverse Events

5.5.1 Post Treatment Follow-up

All subjects receiving any amount of drug will be followed until Day 100. All protocol-defined AEs occurring during the treatment and post-treatment periods should be fully evaluated by the Investigator, recorded on the appropriate pages of the CRF, and documented in the subjects' files.

SAEs that occur after the subject has completed the clinical trial generally need not to be reported unless the Investigator believes the event may be related to the administration of study drug.

5.5.2 Post Adverse Event Follow-up

All subjects that experience AEs should be followed until the event resolves or until the subject's participation in the study ends. For those AEs judged by the Investigator to be unrelated to study drug, the outcome at last observation should be recorded in the appropriate pages of the CRF.

Subjects experiencing AEs that were judged by the Investigator to be possibly, probably or definitely related to study drug should be followed until the events resolve or until the Investigator judges the event to be "chronic" or "stable." Resolution of such events are to be documented on the appropriate CRF pages.

6.0 DATA ANALYSIS AND STATISTICAL METHODS

Data will be analyzed for safety, pharmacokinetics, and biological endpoints. Safety analyses will include all subjects who received any amount of study drug. Analyses of the population of subjects who completed treatment may also be performed. Since the sample size is small, no formal statistical testing will be performed. Study results will be summarized by descriptive statistics and to generate by preliminary hypotheses by making comparisons to other patient cohorts when similar measures were made in patients with similar transplant-related characteristics.

6.1 Disposition of the Study Subjects and Conduct of the Study

The disposition of subjects will be described with summaries by dose group of the number of subjects enrolled, the number of subjects treated, and the number of subjects for whom study drug was permanently discontinued (including the reasons).

6.2 Determination of Sample Size

The sample size for this study is based primarily on PK/PD considerations. The planned accrual is six subjects in each of the five successive dose groups. Formal sample size calculations were not done but past experience with this type of study suggests that this number of subjects will be adequate to characterize the PK/PD profile at each dose level.

6.3 Analysis of Treatment Group Comparability

Demographic (age, sex, etc.) and baseline characteristics (pre-treatment variables and conditioning regimens) will be summarized by dose group.

6.4 Biological and Clinical Activity Analysis

The median time to engraftment will be compared by dose group. The mean of inflammatory markers (e.g., LPS and LBP) and opebacan plasma levels will be summarized by assessment time point and dose group. Subjects with inflammatory states such as presence of fever and infection will be summarized and correlated with level of inflammatory markers such as LPS and LBP by dose group. Transplant-related complications will be described for individual subjects experiencing such events and summarized, if sufficient cases warrant such summarization (see Section 3.8.1).

6.5 Safety Analysis

Safety analyses will involve examination of the incidence, severity, and type of treatment-emergent AEs reported, and changes in vital signs and laboratory test results from baseline (Day −1) to specified time points throughout the study.

6.5.1 Adverse Events

Treatment-emergent AEs reported during the study will be coded using the MedDRA dictionary. An AE will be summarized by dose group and the following:

- System organ class and preferred term
- System organ class and severity
- System organ class, preferred term and severity

These summaries will be presented for the following subsets:

- All adverse events
- Drug-related adverse events
- Adverse events resulting in discontinuation of study drug or the study
- Serious adverse events, including deaths
- Adverse events due to infectious and non-infectious regimen-related toxicities

For tables classifying AEs by severity, if a subject has multiple occurrences of an adverse event with the same MedDRA term, the most severe event will be chosen.

A summary and by-subject listing will be provided for any subject who died, experienced SAEs, or experienced AEs resulting in discontinuation of study drug or the study.

6.5.2 Clinical Laboratory Evaluation

Laboratory values outside of the corresponding normal ranges will be identified and reported to the Sponsor.

The incidence of clinically significant abnormal changes in laboratory values compared with baseline (hematology, chemistry, and urinalysis), and the incidence of DLTs will be summarized by dose group (see Section 3.3.2 for the definition of DLT). MTD will have been exceeded if two or more subjects in a dose group experience a DLT. If, in the subsequent DSMB review of the data, no explanation for the serious adverse events other than use of study drug can be determined, the study may be stopped.

Changes from baseline in special studies parameters (which may include, but may not be limited to, LPS, LBP, BPI, soluble CD14, TLR4, membrane CD14, TNF-RI, IL-1, IL-6, IL-8, IL-10, IL-12 p70 and CRP) will be summarized by dose group.

6.5.3 Vital Signs

For each assessment of vital signs, changes in vital signs from baseline will be summarized. Vital signs (including body weight, heart rate, blood pressure, and temperature) will be summarized by dose group and assessment time.

6.5.4 Antibody Response to Opebacan

The proportion of subjects with an antibody response to opebacan will be summarized by dose group.

6.5.5 Concomitant Medication

Concomitant medications will be classified according to ATC codes in the World Health Organization Drug (WHODRUG) dictionary. The number and percent of subjects using each class of concomitant medication will be tabulated by dose group. The table will be sorted alphabetically.

6.6 Missing Data

Safety results will be summarized using all available data. Multiple attempts will be made to obtain missing data. Missing data will not be imputed.

6.7 Pharmacokinetic, Pharmacodynamic, and Other Analyses

Non-compartmental pharmacokinetic analysis will be performed on the data. The following pharmacokinetic parameters will be obtained: peak concentration after the 30 minute dose, steady state concentration during long-term infusion, endogenous BPI levels measured at predose and 24 hours after the end of the last infusion, and opebacan clearance.

6.8 Interim Analyses

There is no formal Interim Analysis for this study. The decision to dose escalate to the next dose group will be based on safety and pharmacokinetic data.

7.0 INVESTIGATOR REQUIREMENTS AND QUALITY CONTROL

7.1 Study Initiation

Before the start of this study, the following documents must be on file with XOMA or a XOMA representative:

- Signed Protocol Signature Page
- Signed Statement of Investigator
- Current *curricula vitae* of Principal Investigator and all subinvestigators
- Signed financial disclosure report for Principal Investigator and all subinvestigators
- Written documentation of IRB approval of the current version of the protocol and informed consent document(s) (each document identified by XOMA protocol number or title and date of approval)
- A copy of the IRB-approved informed consent document(s) and any translation(s). The informed consent document(s) must be reviewed and approved by XOMA.
- Written documentation of IRB review and approval of any advertising materials to be used for study recruitment, if applicable
- Institutional Review Board (IRB) membership list and/or Department of Health and Human Services number
- Current laboratory certification of the laboratories performing the analyses (if other than a XOMA-approved central laboratory), as well as current normal laboratory ranges for all laboratory tests
- A signed clinical research/site agreement
- Any additional regulatory documentation as required by local and national regulations (completed by the Investigator)

7.2 Study Completion

The following data and materials are required by XOMA before a study can be considered complete or terminated:

- Laboratory findings, clinical data, and all special test results from screening through the end of the study follow-up period
- Case Report Forms (including correction forms) properly completed by appropriate study personnel and signed and dated by the Investigator
- Completed drug accountability records
- Signed copies of all protocol amendment signature pages (if applicable)

- Copies of IRB approval documentation for all protocol amendments (if applicable)
- Copies of all IRB informed consent documents (if applicable)
- A copy of the Principal Investigator's study summary prepared for her/his IRB

7.3 Informed Consent

A sample informed consent document will be provided. No major deviations should be made from the sample informed consent document without prior XOMA approval. The informed consent document must be signed by the subject or the subject's legally authorized representative before the subject participates in the study. A certified translation of the informed consent will be provided, if appropriate. Signed consent forms must be maintained as part of the Investigator's study file and must be available for verification by study monitors at any time. A copy of the signed informed consent document must be provided to the subject or the subject's legally authorized representative.

7.4 Institutional Review Board Approval

This protocol, the informed consent document(s), and relevant supporting information must be approved by the IRB before the study is initiated. The study will be conducted in accordance with U.S. FDA, applicable national and local health authorities, and IRB requirements.

The Principal Investigator is responsible for keeping the IRB apprised of the progress of the study and of any changes made to the protocol as deemed appropriate, but at a minimum the IRB must be updated at least once a year. The Principal Investigator must also keep the IRB and XOMA informed of any significant or serious adverse events.

Investigators are required to promptly notify their IRBs of all adverse events that are both serious and unexpected. This generally refers to serious adverse events that are not already identified in the Investigator's Brochure and are considered possibly or probably related to the study drug by the Investigator. Some IRBs may have additional adverse event reporting requirements with which Investigators are expected to comply. Investigators must immediately forward to their IRB all written safety reports or updates provided by XOMA (e.g., safety reports, the Investigator's Brochure, safety amendments and updates).

7.5 Ethical Considerations

This study will be conducted in accordance with the ethical principles stated in the most recent version of the Declaration of Helsinki or the applicable guidelines on good clinical practice, whichever affords the greater protection to the individual.

7.6 Study Monitoring Requirements

Site visits will be conducted by an authorized XOMA representative to inspect study data, subjects' medical records, and CRFs in accordance with current U.S. GCPs and the respective local and national government regulations and guidelines (if applicable). The Principal Investigator will permit authorized representatives of XOMA, the FDA, and appropriate national or local health authorities to inspect facilities and records relevant to this study.

7.7 Case Report Forms

CRFs for this study will be designed and distributed by authorized XOMA representatives. XOMA will be responsible for data management of this trial, including quality checking of CRFs. In the event of incomplete or inconsistent data, requests for data correction will be sent to the site for resolution. A complete audit trail of changes to the data will be maintained and made available in the clinical trial database. Central laboratory data will be collected electronically, and XOMA's standard procedures will be used to validate electronic transfer of this data. Records retention for study data will be consistent with XOMA's standard procedures. Routine system backup and archiving will also be performed based on XOMA's standard procedures.

7.8 Study Medication Accountability

All study drug required for completion of this study will be provided by XOMA. The recipient will report receipt of the drug, shipment content, and condition by returning the form accompanying the drug shipment. Damaged supplies will be replaced.

Accurate records of all study drug dispensed from and returned to the study site must be recorded by using a drug inventory log.

The site must not dispose of or return any study drug (used or unused) until directed to do so by an authorized XOMA representative. After the study is completed, an authorized XOMA representative will perform final study drug accountability.

7.9 Disclosure of Data

Subject medical information obtained by this study is confidential, and disclosure to third parties other than those noted below is prohibited.

With the permission of the subject or her/his legally authorized representative, the subject's medical information may be given to her/his personal physician or other appropriate medical personnel responsible for her/his welfare.

Data generated by this study must be available for inspection upon request by representatives of the U.S. FDA, national and local health authorities, XOMA or its designee, and the IRB for each study site, if appropriate.

7.10 Retention of Records

The Principal Investigator must comply with U.S. FDA IND regulations, the record retention policies of the relevant national and local health authorities, and the terms agreed upon in the clinical research/site agreement.

7.11 Quality Assurance

An authorized XOMA representative will check original source documents and clinical report forms at the study site. The study may be audited by XOMA, its representatives, and/or regulatory agencies at any time.

8.0 REFERENCES

Abreu MT and Arditi M. Innate immunity and toll-like receptors: clinical implications of basic science research. J Pediatr 2004;144(4):421-9.

Akira S and Takeda K. Toll-like receptor signaling. Nat Rev Immunol 2004;4(7):499-511.

Cooke KR, Gerbitz A, Crawford JM, et al. LPS antagonism reduces graft-versus-host disease and preserves graft-versus-leukemia activity after experimental bone marrow transplantation. J Clin Invest 2001;107(12):1581-9.

Cooke KR, Hill GR, Crawford JM, et al. Tumor necrosis factor- alpha production to lipopolysaccharide stimulation by donor cells predicts the severity of experimental acute graft-versus-host disease. J Clin Invest 1998;102(10):1882-91.

Cooke KR, Hill GR, Gerbitz A, et al. Tumor necrosis factor-alpha neutralization reduces lung injury after experimental allogeneic bone marrow transplantation. Transplantation 2000;70(2):272-9.

Cooke KR, Olkiewicz K, Erickson N and Ferrara JL. The role of endotoxin and the innate immune response in the pathophysiology of acute graft versus host disease. J Endotoxin Res 2002;8(6):441-8.

Dahlberg PS, Acton RD, Battafarano RJ, et al. A novel endotoxin antagonist attenuates tumor necrosis factor-α secretion. J Surg Res 1996;63:44-48.

Demetriades D, Smith JS, Jacobson LE, et al. Bactericidal/permeability-increasing protein ($rBPI_{21}$) in patients with hemorrhage due to trauma: results of a multicenter phase II clinical trial. Trauma 1999;46(4):667-677.

Gazzano-Santoro H, Parent JB, Grinna L, et al. High-affinity binding of the bactericidal/permeability-increasing protein and a recombinant amino-terminal fragment to the lipid A region of lipopolysaccharide. Infect Immun 1992;60(11):4754-4761.

Giroir B. Bactericidal/Permeability-Increasing Protein(BPI)-Lesson learned from the phase III, randomised, clinical trial of $rBPI_{21}$ for adjunctive treatment of children with severe meningococcal sepsis. Crit Care Med 2001;(29):5130.

Hoffman JK, C.; Janeway; Ezekowitz, R. Phylogenetic perspectives in innate immunity. Science 1999;(284):1313.

Holler E. Inflammatory reactions induced by pretransplant conditioning an alternative target for modulation of acute GvHD and complications following allogeneic bone marrow transplantation. 1995;(3-4):217-224.

Janeway CA, Jr. and Medzhitov R. Innate immune recognition. Annu Rev Immunol 2002;20:197-216.

Jin H, Yang R, Marsters S, et al. Protection against endotoxic shock by bactericidal/permeability-increasing protein in rats. J Clin Invest 1995;95:1947-1952.

Karnofsky D. The Clinical Evaluation of Chemotherapeutic Agents in Cancer. Columbia University Press 1949:196.

Lechner AJ, Lamprech KE, Johanns CA and Matuschak GM. The recombinant 23-kDa N-terminal fragment of bactericidal/permeability-increasing protein ($rBPI_{23}$) decreases *Escherichia coli*-induced mortality and organ injury during immunosuppression-related neutropenia. Shock 1995;4(4):298-306.

Levin M, Quint PA, Goldstein B, et al. Recombinant bactericidal/permeability-increasing protein ($rBPI_{21}$) as adjunctive treatment for children with severe meningococcal sepsis: a randomised trial. $rBPI_{21}$ Meningococcal Sepsis Study Group. Lancet 2000;356(9234):961-967.

Levy O. A neutrophil-derived anti-infective molecule: bactericidal/permeability-increasing protein. Antimicrob Agents Chemother 2000;44(11):2925-31.

Levy O. Therapeutic potential of the bactericidal/permeability-increasing protein. Expert Opin Investig Drugs 2002;11(2):159-67.

Levy O. Antimicrobial proteins and peptides: anti-infective molecules of mammalian leukocytes. J Leukoc Biol 2004;76(5):909-25.

Levy O, Ooi CE, Elsbach P, Doerfler ME, Lehrer RI and Weiss J. Antibacterial proteins of granulocytes differ in interaction with endotoxin. Comparison of bactericidal/permeability-increasing protein, p15s, and defensins. J Immunol 1995;154:5403-5410.

Levy O, Sisson RB, Fryer HE, et al. Neutrophil defense in patients undergoing bone marrow transplantation: bactericidal/permeability-increasing protein (BPI) and defensins in graft-derived neutrophils. Transplantation 2002;73(9):1522-6.

Levy O, Teixeira-Pinto A, White ML, et al. Endotoxemia and elevation of lipopolysaccharide-binding protein after hematopoietic stem cell transplantation. Pediatr Infect Dis J 2003;22(11):978-81.

Lin Y, Kohn FR, Kung AHC and Ammons WS. Protective effect of a recombinant fragment of bactericidal/permeability increasing protein against carbohydrate dyshomeostasis and tumor necrosis factor-α elevation in rat endotoxemia. Biochem Pharmacol 1994a;47(9):1553-1559.

Lin Y, Leach WJ and Ammons WS. Synergistic effect of a recombinant N-terminal fragment of bactericidal/permeability-increasing protein and cefamandole in treatment of rabbit gram-negative sepsis. Antimicrob Agents Chemother 1996;40(1):65-69.

Nijveldt RJ, Wiezer MJ, Meijer C, et al. Major liver resection results in a changed plasma amino acid pattern as reflected by a decreased Fischer ratio which improves by bactericidal/permeability increasing protein. Liver 2001;21(1):56-63.

Opal SM, Palardy JE, Marra MN, Fisher CJ, Jr., McKelligon BM and Scott RW. Relative concentrations of endotoxin-binding proteins in body fluids during infection. Lancet 1994;344:429-431.

Orange JS, Levy O and Geha RS. Human disease resulting from gene mutations that interfere with appropriate nuclear factor-kappaB activation. Immunol Rev 2005;203:21-37.

Przepiorka D. Meeting report-Consensus conference on acute GVHD grading. Bone Marrow Transplant 1995;(15):825-828.

Rogy MA, Moldawer LL, Oldenburg HS, et al. Anti-endotoxin therapy in primate bacteremia with HA-1A and BPI. Ann Surg 1994;220(1):77-85.

Ulevitch RJ and Tobias PS. Recognition of gram-negative bacteria and endotoxin by the innate immune system. Curr Opin Immunol 1999;11(1):19-22.

von der Mohlen M. Inhibition of endotoxin-induced activation of the coagulation and fibrinolytic pathways using recombinant endotoxin-binding protein ($rBPI_{23}$). Blood 1995a;(83):3437.

von der Mohlen MAM, Kimmings AN, Wedel NI, et al. Inhibition of endotoxin-induced cytokine release and neutrophil activation in humans using recombinant bactericidal/permeability-increasing protein. J Infect Dis 1995;172:144-151.

Weiss J, Elsbach P, Shu C, Castillo J, Grinna A and Theofan G. Human bactericidal/permeability-increasing protein and a recombinant NH_2-terminal fragment cause killing of serum-resistant gram-negative bacteria in whole blood and inhibit tumor necrosis factor release induced by the bacteria. J Clin Invest 1992;90(3):1122-1130.

Wiezer MJ, Langendoen SI, Meijer C, et al. Pharmacokinetics of a recombinant amino terminal fragment of bactericidal/permeability increasing protein ($rBPI_{21}$) after liver surgery in rats and humans. Shock 1998;10(3):161-166.

Wiezer MJ, Meijer C, Sietses C, et al. Bactericidal/permeability-increasing protein preserves leukocyte functions after major liver resection. Ann Surg 2000;232(2):208-215.

Yu B and Wright SD. Catalytic properties of lipopolysaccharide (LPS) binding protein. Transfer of LPS to soluble CD14. J Biol Chem 1996;271(8):4100-5.

Appendix 1: Study BPSC030 Schedule of Assessments

Timepoint	Donor[1]	Prescreen[2]	Day –3	Final Screen[3]	Predose[4]	Postdose[4]	HSCT[4]																				100\aGvHD[6]	Discharge
Day					–1		0	1	2	3	4	5	6	7	8	9	10	11	12	13	14[5]	21[5]	28[5]	35[5]	42[5]	49[5]		
Signed informed consent	X	X																										
Inclusion/exclusion		X		X																								
Demographics, med. History[7]	X	X		X	X																							
HIV antibody		X																										
ECG		X																										
Pregnancy test		X																										
DNA assessment[8]	X	X																										
Adverse events					X[9]	X	X	X	X	X	X	X	X	X	X	X	X	X	X	X	X	X	X	X	X	X	X	X
Concomitant medications		X		X	X	X	X	X	X	X	X	X	X	X	X	X	X	X	X	X	X	X	X	X	X	X	X	X
**Opebacan dosing (3-day)**						X →			X																			
PK for 3-day cohorts[10]	X[11]	X[11]			X[11]	X	X	X	X	X																	X	
Chem./coag./urinalysis[12]		X			X					X											X	X	X				X	
CBC w/ diff., platelets[13]		X			X		X		X	X				X			X				X	X	X	X	X	X	X	
**Opebacan dosing (7-day)**						X →							X															
PK for 7-day cohort[10]	X[11]	X[11]			X[11]	X	X	X					X	X													X	
Chem./coag./urinalysis[12]		X			X									X							X	X	X				X	
CBC w/ diff., platelets[13]		X			X		X						X	X			X				X	X	X	X	X	X	X	
**Opebacan dosing (14-day)**						X →														X								
PK for 14-day cohort[10]	X[11]	X[11]			X[11]	X	X	X					X				X			X	X						X	
Chem./coag./urinalysis[12]		X			X										X						X	X	X				X	
CBC w/ diff., platelets[13]		X			X		X							X			X			X	X	X	X	X	X	X	X	
Metabolic assessment[14]				X		X →																				X		X
Physical exam		X																					X				X	X
Vital signs[15]		X			X →																					X	X	X
Fever (T_{max})[16]					X	X	X	X	X	X	X	X	X	X	X	X	X	X	X	X								
LPS and LBP	X	X	X[17]		X		X		X		X		X		X		X				X	X	X	X			X	
LPS-induced TNF and IL-6[18]	X	X			X																X	X	X				X	
Soluble markers[19]					X		X		X					X							X	X	X	X			X	
Surface markers[20]			X		X		X		X					X							X	X	X	X			X	
Oral mucositis score[21]		X			X		X →																			X		
Cardiac serology[22]		X			X			X			X				X						X	X	X	X	X	X		
LVEF by ECHO or MUGA		X			X									X							X	X	X				X	
aGvHD grading[23]														X							X	X	X		X		X	
Performance score[24]					X									X							X	X	X	X	X	X		
Antibody response	X	X																					X					

Appendix 1 *(continued)*
Footnotes to Study BPSC030 Schedule of Assessments

1 Assessments performed on consenting donors must be performed prior to stem cell harvest.

2 Prescreening assessments must take place within 30 days of admission to the hospital for the conditioning regimen and subsequent HSCT procedure.

3 Final screening must take place within 12 hours prior to the initiation of treatment with opebacan. Prior to opebacan administration, the Principal Investigator or designee must review and approve subject eligibility and must confirm that the interval between initiating opebacan and administering donor cells will be between 18 and 36 hours.

4 The HSCT procedure must be performed no less than 18 hours and no more than 36 hours after completion of chemotherapy treatment. Subjects whose conditioning regimen consists of chemotherapy only will receive a continuous opebacan infusion following completion of chemotherapy treatment on Day –1. Subjects whose conditioning regimen includes TBI will have three 1-hour interruptions in their opebacan infusions (one on Day –1 and two on Day 0) to accommodate TBI treatment. Following the final interruption on Day 0, the infusion must be restarted at least 2 hours prior to the HSCT.

5 All assessments scheduled on Days 14, 21, and 28 must be completed for all subjects whether or not the subjects have been discharged from the hospital. Assessments scheduled on Days 35, 42 and 49 will be performed only for those subjects who are still hospitalized on those days. Note that assessments taking place between Day 14 and Day 49 (excluding Days 21, 28, 35, and 42) are not shown in the Schedule of Assessments, though they are mentioned in the footnotes for the assessments for the metabolic assessment (Footnote 14), vital signs (Footnote 15), and oral mucositis score (Footnote 21).

6 The assessments marked for Day 100/aGvHD should be performed at the Day 100 (± 14 day) final follow-up visit and whenever a subject is diagnosed with ≥ grade 2 aGvHD (see Appendix 4 for Consensus Grading Scale). The Day 100 visit should be performed in the clinic, if possible. If this is not possible, study personnel should call the subject or subject's attending physician to obtain as much of the Day 100 assessment information as possible.

7 Demographics and medical history, including virology and serology status taken at prescreen; interim medical history taken at final screen and predose.

8 DNA testing includes SNPs for markers such as TLR and TNF at select sites only. DNA samples obtained from consenting donors will be taken at the time of stem cell harvest.

9 The "Signs and Symptoms Inventory" CRF page must be completed on Day –1 prior to dosing.

10 See Appendix 2 for a table showing the multiple PK sample collection time points on Days –1, 2, 6 and 13 not captured in this table. For Dose Groups 1, 2, and 3, multiple PK time points occur on Days –1 and 2. For Dose Group 4, multiple PK time points occur on Days –1 and 6. For Dose Group 5, multiple PK time points occur on Days –1 and 13. Note: PK sample must be drawn from a different vein than that used for opebacan administration.

11 The PK samples for the donor and subject at prescreen and for the subject at predose on Day –1 are used for measuring the level of endogenous BPI.

Appendix 1 *(continued)*
Footnotes to Study BPSC030 Schedule of Assessments

[12] Chemistries include sodium, potassium, chloride, bicarbonate, glucose, BUN, creatinine, calcium, phosphorus, magnesium, bilirubin (total, and direct if bilirubin > 2.0 mg/dL), albumin, ALT, AST, LDH, alkaline phosphatase, CPK with MB fraction (not done if a cardiac serology assessment is done), and total protein. Coagulation includes PT, PTT, fibrinogen, D-Dimers (or FDP or FSP). Urinalysis includes chemistry and microscopic.

[13] Successful engraftment must be confirmed by three consecutive daily measurements of ANC ≥ 500/µL, with the first of the 3 days occurring no later than Day 28.

[14] The metabolic assessment includes the following: 1) Caloric source documentation, including volume and type for all fluids and nutritional sources, performed on Days –1, 0, 3, 7, 10, 14, 17, 21, 24, 28, 35, 42, and 49 or until the subject is released from the hospital, whichever occurs first; and 2) Intake and output (I&O) and total days of parenteral nutrition (TPN), performed daily from Day –1 through Day 49 or until the subject is released from the hospital, whichever occurs first. At final screening, the subject or legal guardian will be asked to provide the subject's recent dietary history. Metabolic assessments requiring daily totals should be measured by calendar day (i.e., from midnight to midnight) unless otherwise specified by institutional standard practice.

[15] Vital signs include pulse, blood pressure (systolic/diastolic), respiratory rate, and temperature taken in the morning (i.e., the first waking set of vital signs). On Day –1 only, body weight must be measured predose to calculate the study drug dose and temperature is measured twice: predose (within one hour prior to the start of infusion) and postdose (within four hours after the start of infusion). If a subject experiences an SAE, vital signs must be taken more frequently, per standard institutional practice. Vital signs must be recorded every day from Day –1 through Day 49 or until the subject is released from the hospital, whichever occurs first.

[16] T_{max} is the highest temperature recorded of all measurements taken during a calendar day (i.e., midnight to midnight). On Day –1 only, T_{max} is measured for two separate periods: predose (within one hour prior to the start of infusion) and postdose (within four hours after the start of infusion).

[17] On Day –3, LPS but not LBP will be assessed.

[18] Samples for LPS-induced TNF and IL-6 collected at select sites only.

[19] Soluble markers may include, but are not limited to, sCD14, TNF-RI, IL-1, IL-6, IL-8, IL-10, IL-12, and CRP.

[20] Surface markers may include, but are not limited to, TLR4 and mCD14. Samples for surface markers will be collected at select sites only.

[21] Oral mucositis will be assessed based on a scoring system adapted from the NCI CTC for Bone Marrow Transplantation (see Appendix 5). The oral mucositis score must be recorded at prescreening and daily from Day –1 through Day 7. After Day 7, the oral mucositis score must be recorded daily until the condition resolves to grade 0. If oral mucositis reappears after resolving, daily assessments must be reinstituted through Day 49 or until the subject is released from the hospital, whichever occurs first.

[22] The cardiac serology assessment includes CPK-MB, troponin T, and troponin I.

[23] aGvHD grading is based on an adapted version of the Consensus Grading for Acute GvHD scale developed by Przepiorka et al. (see Appendix 4). In addition to the days checked in the schedule, this assessment should be performed on Days 56, 70, and 84, if possible.

[24] The Karnofsky Performance Criteria is used to rate subjects (see Appendix 3).

Appendix 2: Study BPSC030 PK Sampling Schedule[1]

Study Day	Prescreen	–1					0	1	2					3	6					7	10	13					14	100
Minutes[2]		Pre	30	45	90	270	1440	2880	EOI	15	30	60	240	1440	EOI	15	30	60	240	1440		EOI	15	30	60	240	1440	
Group 1	X[3]	X[3]	X	X	X	X	X	X	X	X	X	X	X	X														X
Group 2	X[3]	X[3]	X	X	X	X	X	X	X	X	X	X	X	X														X
Group 3	X[3]	X[3]	X	X	X	X	X	X	X	X	X	X	X	X														X
Group 4	X[3]	X[3]	X	X	X	X	X	X							X	X	X	X	X	X								X
Group 5	X[3]	X[3]	X	X	X	X	X	X							X[4]						X	X	X	X	X	X	X	X
Donor	X[3]																											

1 PK sample must be drawn from a different vein than that used for opebacan administration.

2 For Days –1, 0, and 1, sampling time is shown in minutes following initiation of opebacan treatment. For Days 2 and 3 (Groups 1, 2, and 3), Days 6 and 7 (Group 4), and Days 13 and 14 (Group 5), sampling times are shown in minutes following the end of infusion (EOI). The "EOI" sample must be drawn within one hour prior to the end of the opebacan infusion. The acceptable windows for the other PK draws are as follows: 15 ± 5 minutes, 30 ± 5 minutes, 45 ± 5 minutes, 60 ± 10 minutes, 90 ± 15 minutes, 240 ± 30 minutes, 270 ± 30 minutes, 1440 ± 60 minutes, and 2880 ± 60 minutes.

3 The PK samples for the donor and subject at prescreen and for the subject at predose on Day –1 are used for measuring the level of endogenous BPI.

4 The PK sample for Group 5 subjects on Day 6 is drawn along with other blood draws on that day. Though it appears in the EOI column, the infusion for this dose group does not end until Day 13.

Appendix 3
Karnofsky Performance Criteria

The Karnofsky performance criteria are shown in the table below.

Percent Functionality	Karnofsky Performance Criteria
100%	Normal; no complaints; no evidence of disease
90%	Able to carry on normal activity; minor signs and symptoms of disease
80%	Normal activity with effort; some signs and symptoms of disease
70%	Cares for self; unable to carry on normal activity or do work
60%	Requires occasional assistance, but is able to care for most personal needs
50%	Requires considerable assistance and frequent medical care
40%	Disabled; requires special care and assistance
30%	Severely disabled; hospitalization indicated although death not imminent
20%	Very sick; hospitalization necessary; requires active support treatment
10%	Moribund; fatal processes progressing rapidly
0%	Dead

Sources: (Karnofsky, 1949)

Appendix 4
Consensus Grading for Acute GvHD

Subjects will be graded for aGvHD by first establishing the staging for extent of organ involvement using the criteria in Table A, and then determining the overall grade using the consensus grading criteria in Table B.

Table A: Staging for Extent of Organ Involvement

Organ	Stage	Extent of Organ Involvement	
Skin	1	A maculopapular eruption involving less than 25% of the body surface[a]	
	2	A maculopapular eruption involving 25-50% of the body surface[a]	
	3	Generalized erythroderma	
	4	Generalized erythroderma with bullous formation	
Liver	1	Bilirubin 2.0 – 2.99 mg/100 mL[b]	
	2	Bilirubin 3 – 5.99 mg/100 mL	
	3	Bilirubin 6 – 14.99 mg/100 mL	
	4	Bilirubin rise to greater than 15 mg/100 mL	
GI		Subjects ≥ 45 kg	Subjects < 45 kg
	1	500 – 1000 mL of stool/day[c] or persistent nausea[d]	10 – 20 mL/kg/day[c] or persistent nausea[d]
	2	> 1000 – 1500 mL of stool/day	> 20 – 35 mL/kg/day
	3	> 1500 mL of stool/day	> 35 mL/kg/day
	4	Severe pain with or without ileus	Severe pain with or without ileus

[a] Use “Rule of Nine” or burn chart to determine extent of rash

[b] Downgrade one stage if an additional cause of elevated bilirubin has been documented

[c] Downgrade one stage if an additional cause of diarrhea has been documented

[d] Persistent nausea with histologic evidence of aGvHD in the stomach or duodenum

Table B: Consensus Grading Scale

Grade	Stage by Organ System		
	Skin	Liver	GI
I	1 – 2	0	0
II	3 and/or	1 and/or	1
III	N/A[b]	2 – 3 and/or	2 – 4
IV[a]	4 and/or	4	N/A[b]

[a] Grade IV may also include lesser organ involvement but with extreme decrease in performance status.

[b] Not applicable, i.e., stage for this organ system does not affect grade on Consensus Grading Scale.

Source: Adapted from Przepiorka et al. (Przepiorka, 1995).

Appendix 5
Oral Mucositis Assessment

The following grading scale, adapted from the CTCAE Version 3.0, must be used in assessing oral mucositis during this study:

Clinical Exam

Grade	Symptoms
0	No changes
1	Erythema of the mucosa
2	Patchy ulcerations or pseudomembranes
3	Confluent ulcerations or pseudomembranes; bleeding with minor trauma
4	Tisue necrosis; significant spontaneous bleeding; life-threatening consequences
5	Death

Functional/Symptomatic

Grade	Symptoms (Upper Aerodigestive Tract)
0	No changes
1	Minimal symptoms, normal diet; minimal respiratory symptoms but not interfering with function
2	Symptomatic but can eat and swallow modified diet; respiratory symptoms interfering with function but not interfering with ADL
3	Symptomatic and unable to adequately aliment or hydrate orally; respiratory symptoms interfering with ADL
4	Symptoms associated with life-threatening consequences
5	Death

A Phase I/II Study of the Safety and Pharmacokinetics of Opebacan ($rBPI_{21}$) in Patients Undergoing Myeloablative Allogeneic Hematopoietic Stem Cell Transplantation (HSCT)
DFCI 06-155

Radiation Exposure/ Chemotherapy

<u>Relevance</u>
- HSCT/aGVHD
- Fever/Neutropenia
- Radiation exposure
- Biodefense

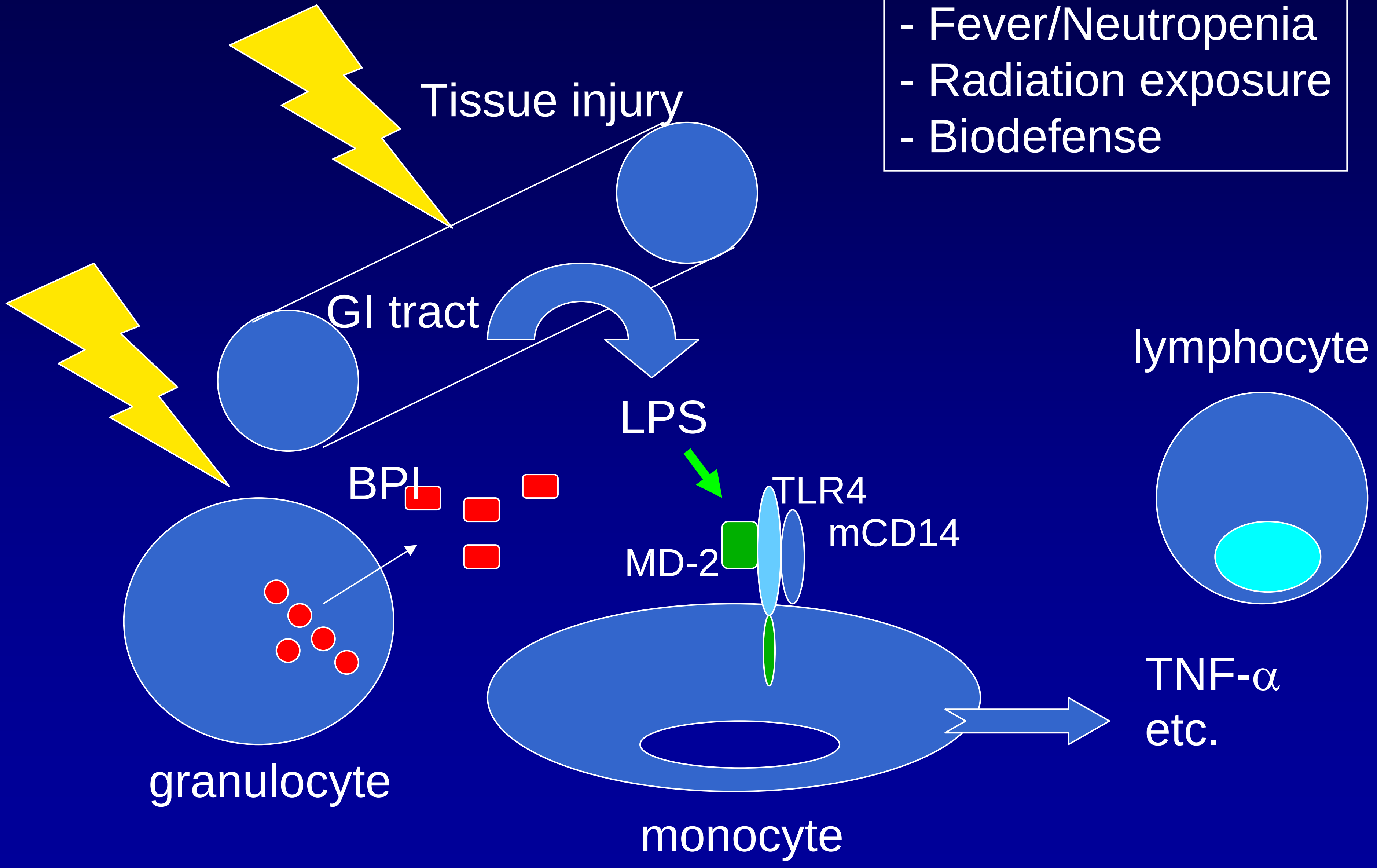

Innate Immunity

<u>Detect</u> foreign molecules (PAMP)

Plasma proteins

Cell surface receptors

Intracellular signaling cascades

<u>Release</u> cytokines

<u>Mobilize</u> defenses:

Kill microbes (O2, NO, antimicrobial proteins/peptides)

Neutralize microbial surface components

BPI <-->LPS

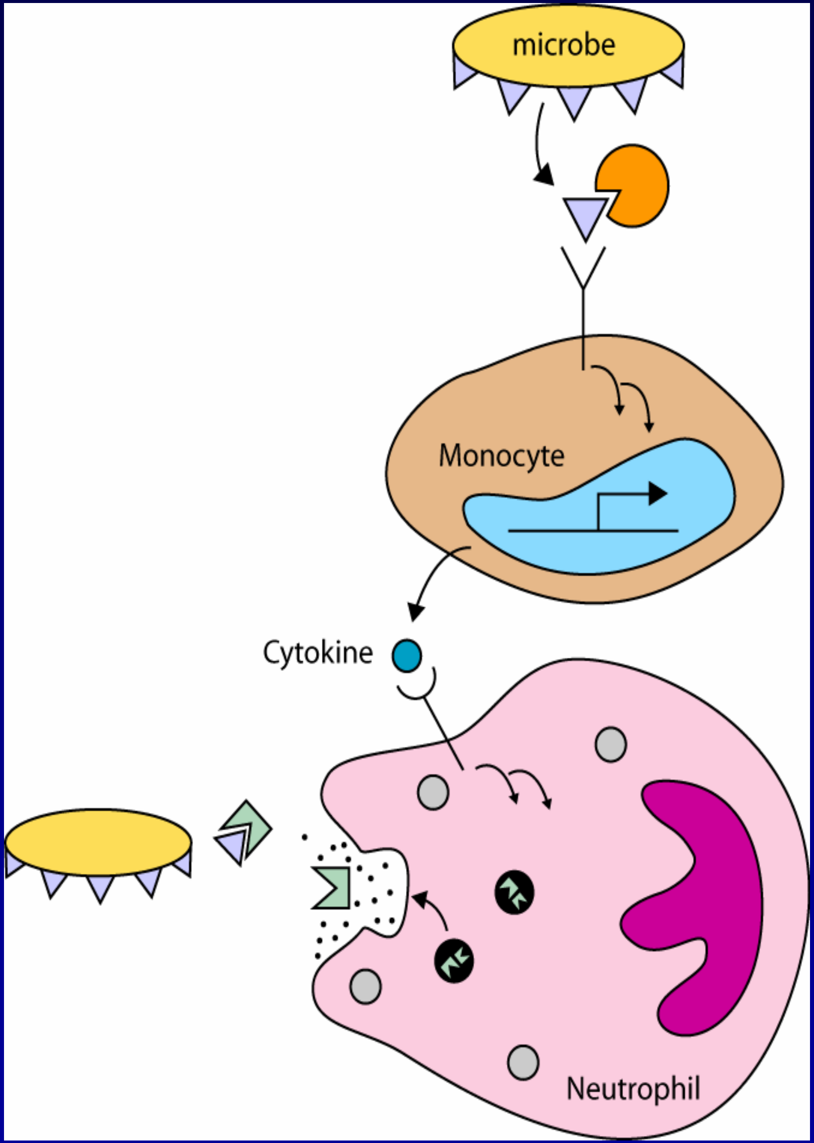

Multiple Host Innate Immune Molecules Bind LPS and Modulate its Activity

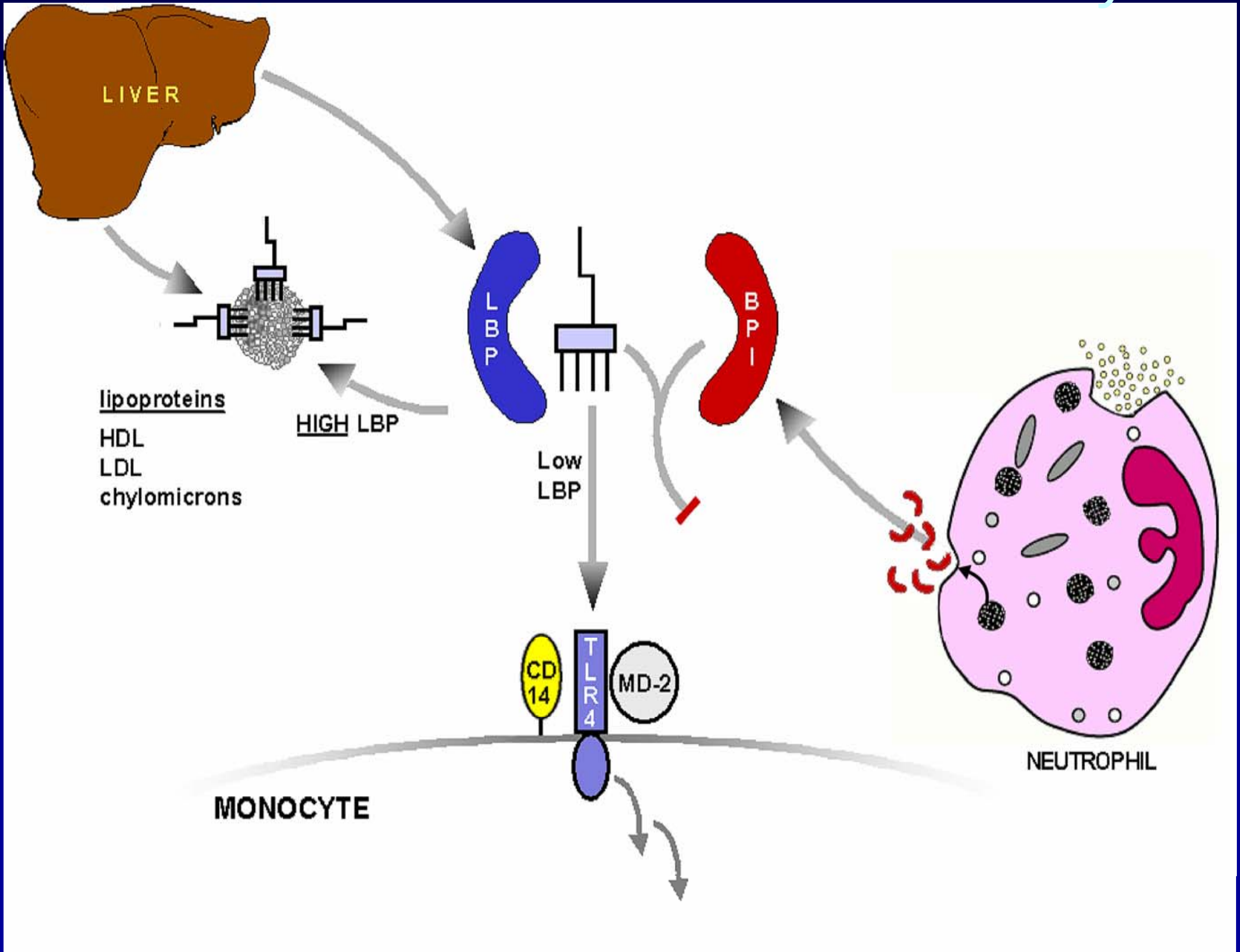

Bactericidal permeability inducing protein (BPI)

- 50-55 kDa cationic protein
- potently (nM) cytotoxic to Gram-negative species
  *increases outer membrane permeability
- LPS-neutralizing properties
  *inhibits LPS signaling
- found primarily in neutrophils

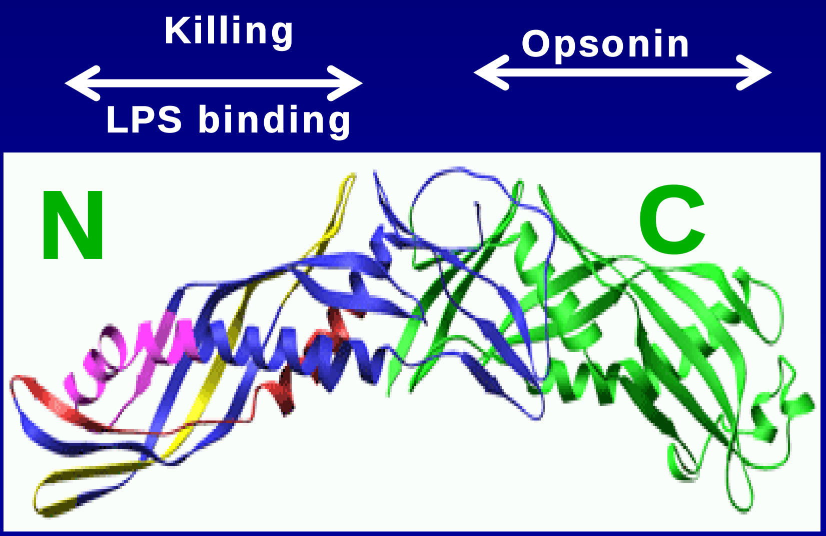

Human Trials of $rBPI_{21}$

- Safety
  - Well-tolerated (> 2,000 humans)
  - Non-toxic
  - Non-immunogenic
- In human volunteers, $rBPI_{21}$ blocks LPS-induced:
  - changes in cardiac index
  - cytokine production
  - activation of clotting cascades

Von der Mohlen *J Infect Dis* & *Blood* 1995

$rBPI_{21}$ is most likely to be beneficial for diseases in which endotoxin plays a prominent role and in which endogenous BPI may be limiting

Neutrophil-defense in patients undergoing BMT

Antimicrobial activity of engrafting neutrophils (day +20 to +100)

Measured granule antimicrobial proteins/peptides/function:

- Bactericidal/permeability-increasing protein (BPI)
- Defensin peptides
- Killing of E. coli K1/r by neutrophil extracts

- BPI and defensin content of patient-derived neutrophil extracts similar to that of normal controls
- BPI range is quite broad
- Severe neutropenia likely renders host deficient in total body content of antimicrobial proteins/peptides

Levy & Guinan (2002) *Transplantation* 73:1522-1526

Endotoxemia and elevation of lipopolysaccharide-binding protein after hematopoietic stem cell transplantation

OFER LEVY, MD, PHD, ARMANDO TEIXEIRA-PINTO, MS, MARK L. WHITE, BS, STEPHEN F. CARROLL, PHD, LESLIE LEHMANN, MD, DAVID WYPIJ, PHD AND EVA GUINAN, MD

THE PEDIATRIC INFECTIOUS DISEASE JOURNAL Vol. 22, No. 11, Nov. 2003

- 11/25 (44%) patients had endotoxemia at some-point after HSCT

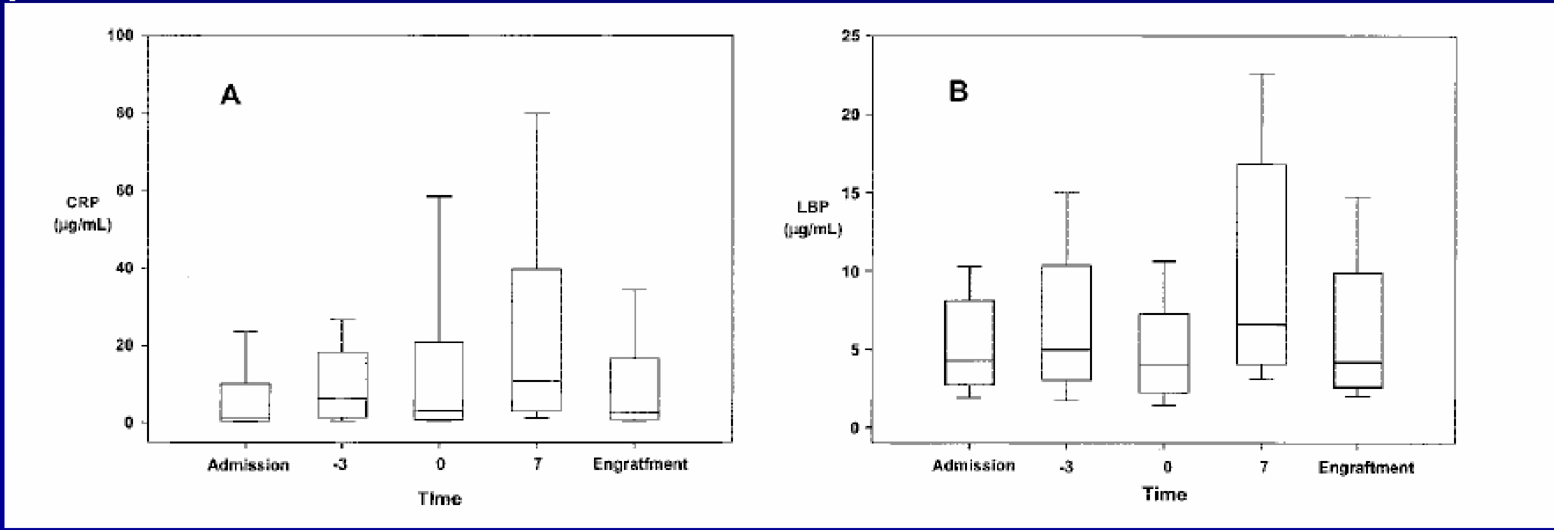

- CRP & LBP elevated at Day 7 and correlated with one another

Tumor Necrosis Factor–α Production to Lipopolysaccharide Stimulation by Donor Cells Predicts the Severity of Experimental Acute Graft-Versus-Host Disease

Kenneth R. Cooke,* Geoffrey R. Hill,* James M. Crawford,‡ David Bungard,* Yani S. Brinson,* John Delmonte, Jr.,* and James L.M. Ferrara*
**Department of Pediatric Oncology Dana-Farber Cancer Institute, Children's Hospital, and Harvard Medical School, Boston, Massachusetts 02115 and the ‡Department of Pathology, Yale University School of Medicine, New Haven, Connecticut 06520-8023*

J. Clin. Invest.
© The American Society for Clinical Investigation, Inc.
0021-9738/98/11/1882/10 $2.00
Volume 102, Number 10, November 1998, 1882–1891

- Murine GVHD model
- LPS-induced TNF-α predicts aGVHD
- Mice with hypomorphic mutations in TLR4 (part of LPS receptor complex) do not get aGVHD
- Interpretation of murine model:

  LPS translocates from gut to systemic circulation and triggers, via TLR4, TNF-α that results in aGVHD

Do endotoxemia and endotoxin-directed innate immunity play a role in human aGVHD?

$rBPI_{21}$ to Prevent aGVHD: Hypothesis

- Myeloablative conditioning regimens followed by HSCT will induce innate immune responses triggered in part by endotoxemia
- Endotoxemia is contributory/necessary (but not sufficient) for subsequent aGVHD
- Neutropenia is associated with low BPI levels
- Prior to engraftment, the BPI/LBP ratio will be low
- If the above is correct, then IV infusion of $rBPI_{21}$ early during HSCT may modulate subsequent aGVHD

Natural History Study Design

Measure endotoxin and endotoxin-directed proteins of the innate immune system in patients undergoing HSCT

- Peripheral blood collected from donors and from pediatric and adult patients undergoing sibling HSCT
  - Donors collected at a single time-point
  - Patients collected
    - Baseline
    - Day 0, +7, +14, +21, +28
    - At the time of diagnosis of aGVHD
- Preliminary correlation with characteristics and outcome

Results

- 30 recipient:donor sibling pairs
- 18 M:12 F
- 17 adult pairs (median age R:D 39:43)
- 13 pediatric pairs (median age R:D 14:11)
- ALL 8, AML 8, CML 4, MDS 4, HD 2, AA 2, CLL 1, NHL 1
- TBI=25 (83%), BU=3 (10%), CTX=30 (100%)

Prolonged neutropenia in patients undergoing HSCT

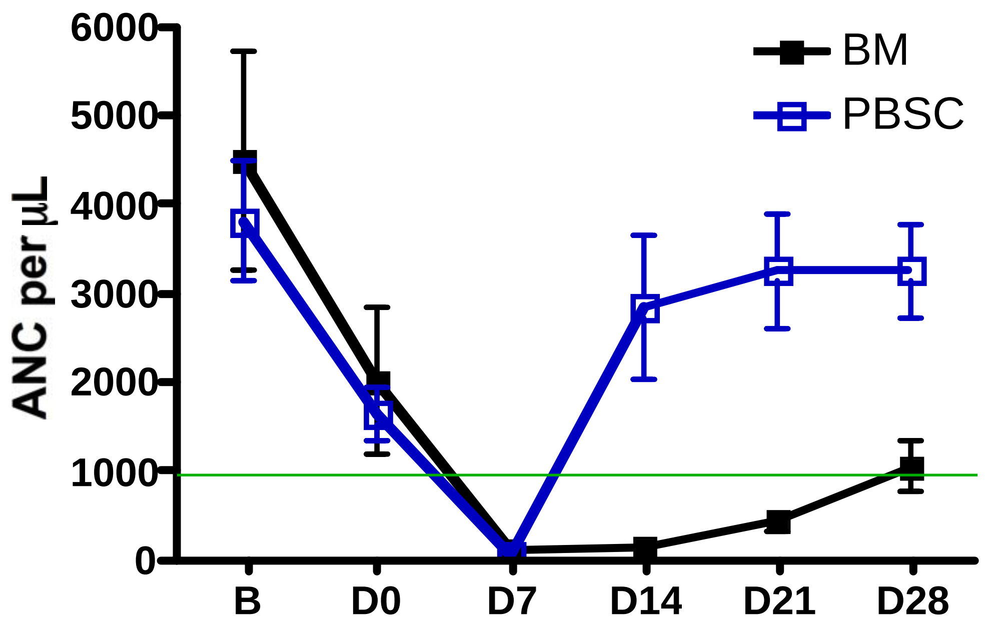

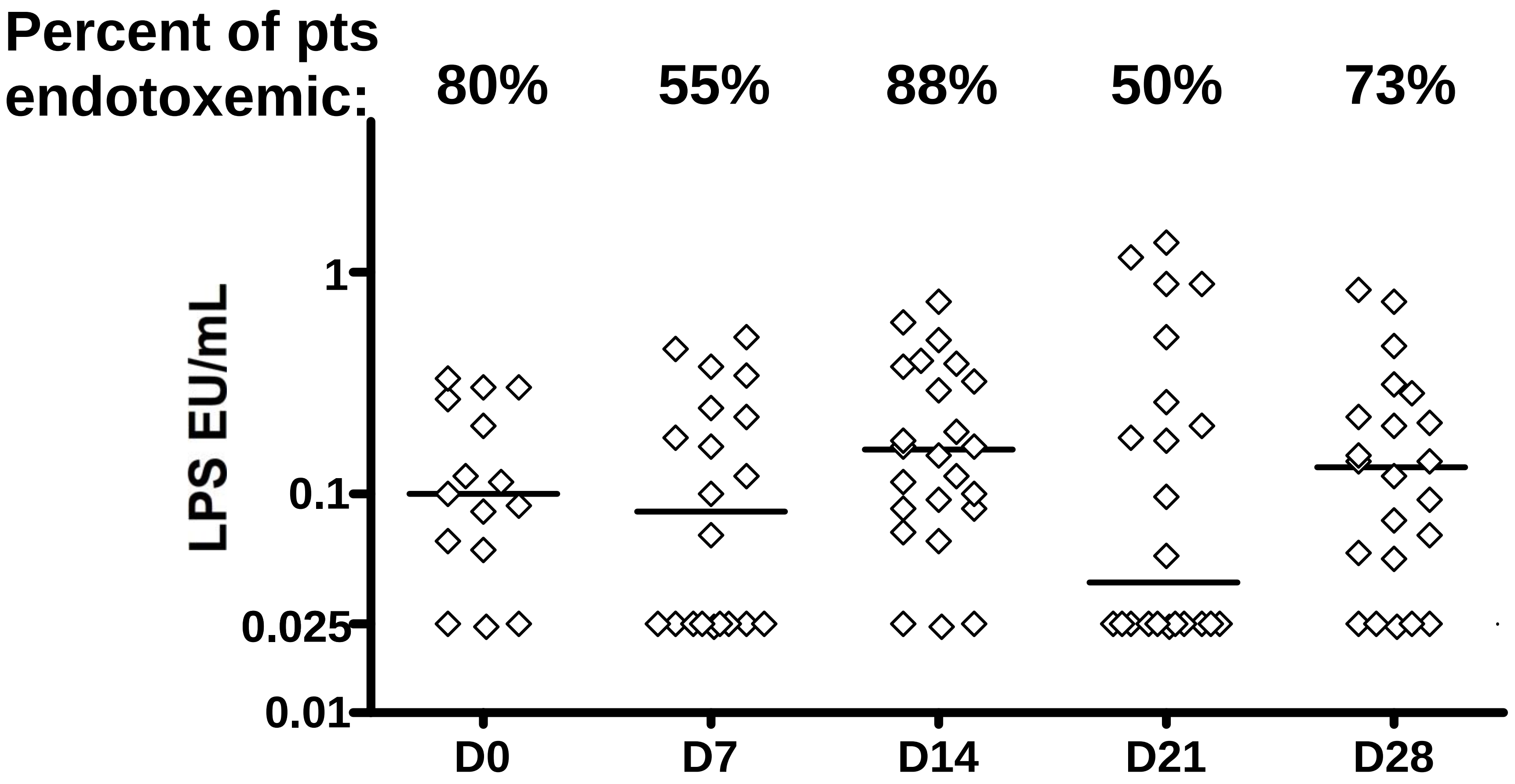
Percent of pts
endotoxemic:
80%
55%
88%
50%
73%
LPS EU/mL
1
0.1
0.025
0.01
D0
D7
D14
D21
D28

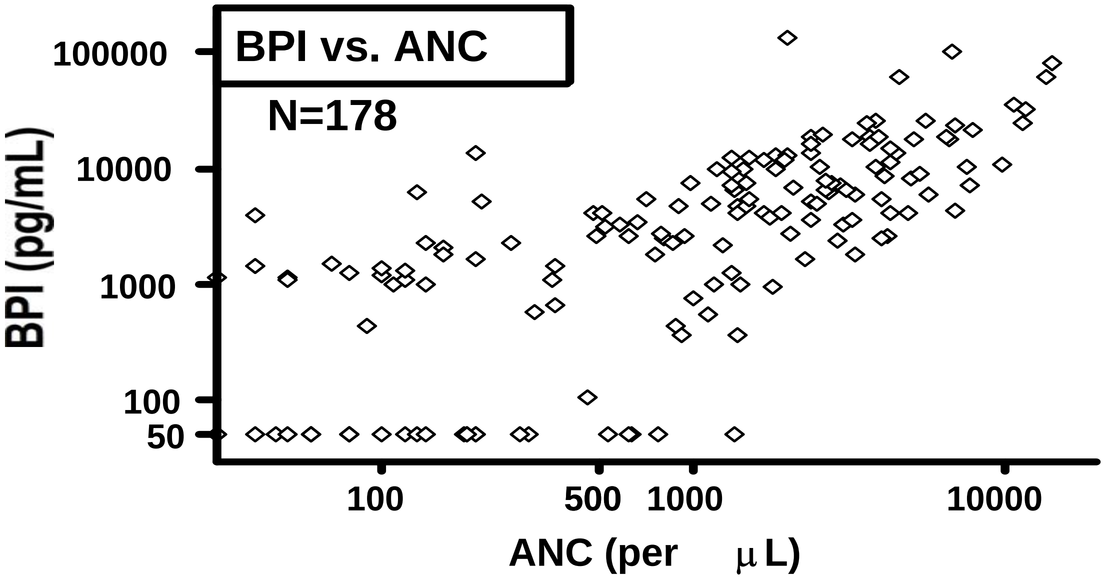
BPI vs. ANC
N=178
BPI (pg/mL)
100000
10000
1000
100
50
100
500
1000
10000
ANC (per μL)

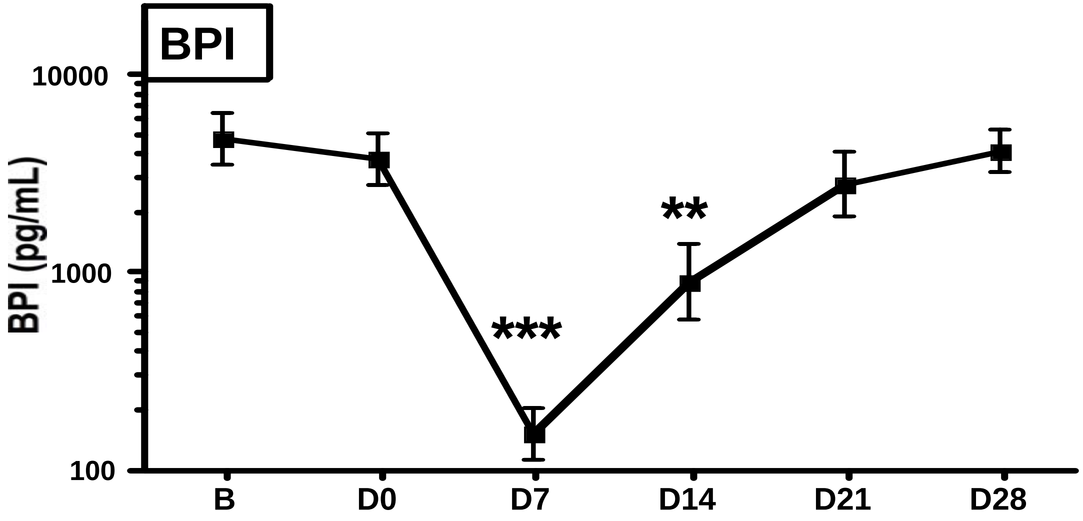
BPI
BPI (pg/mL)
10000
1000
100
***
**
B
D0
D7
D14
D21
D28

Lower Plasma BPI/LBP Ratio Associated With AGVHD

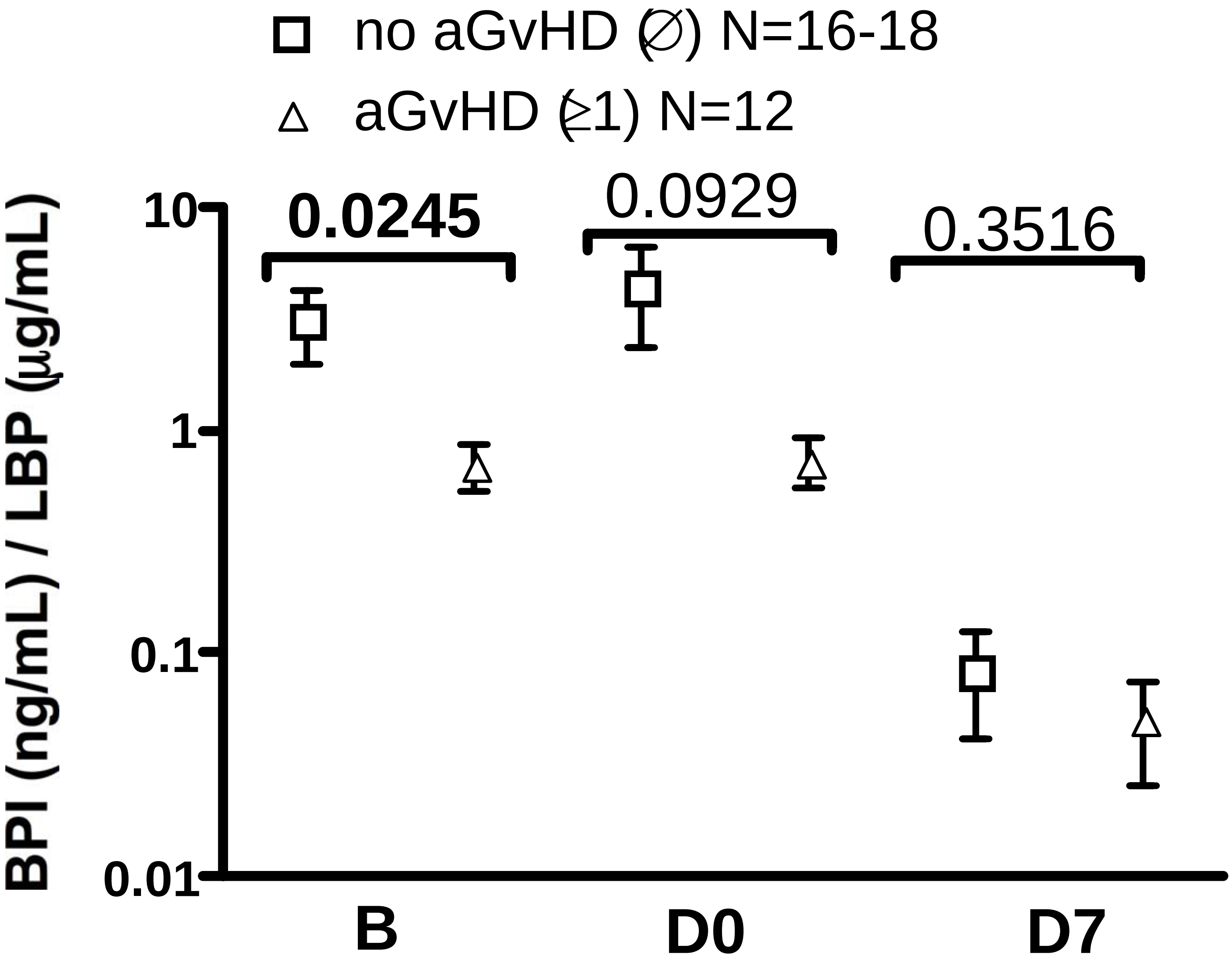

Features of patients who develop aGVHD

- Lower plasma BPI at baseline
- Higher plasma LBP at baseline
- Other evidence of LPS-related innate immune activation at baseline and day 0

<u>Consistent with hypothesis</u>:

- LPS → TLR4 → TNF-α → aGVHD
- Addition of BPI may shield against this process

Protocol Objectives

- To demonstrate safety of escalating doses of opebacan in myeloablative allogeneic HSCT
- To determine pharmacokinetics of opebacan in myeloablative allogeneic HSCT
- To determine if IV opebacan is associated with changes in biological markers for inflammation
- To develop preliminary descriptive data on the occurrence and severity of HSCT-related complications, including acute GvHD

Study Design

- Phase I/II, open-label, dose-escalation study of the safety and PK of opebacan in subjects undergoing non-T cell depleted myeloablative allogeneic HSCT

- Subjects who sign informed consent and pass initial screening prior to HSCT conditioning will be re-evaluated at Day –1 following completion of conditioning

- Those subjects who meet eligibility criteria at final screening will receive opebacan by continuous IV infusion beginning on Day –1

- Eligible subjects will be assigned to one of five successive dose groups of six subjects each. Each dose group must contain at least two pediatric subjects and two adult subjects.

Pre-Screen Inclusion Criteria

- Subject and/or legal guardian have signed consent
  IV catheter maintained in subject for the purpose of opebacan administration
- Age ≤ 60 and undergoing allogeneic myeloablative HSCT
- Life expectancy ≥ 8 weeks
- Scheduled for treatment with a myeloablative conditioning regimen
- Meets standard performance and end-organ function criteria for HSCT
- Female subjects of child-bearing age - negative urine pregnancy test
- Sexually active male and female subjects of reproductive age must be using a form of contraception considered effective and medically acceptable

Pre-Screen Exclusion Criteria

- **Cumulative lifetime exposure of > 300 mg/M^2 of anthracycline (expressed as doxorubicin equivalent dose)**
- **Prior history of CHF**
- **Active infection or ongoing treatment with antibiotics prior to transplant**
- **Positive for HIV,HTLV-I or HTLVII**
- **Any prior stem cell transplant**
- **Projected need for heparin anticoagulation during the study medication infusion period. Use of dextran sulfate is also prohibited during the infusion period**
- **Enrollment on a regimen where conditioning extends beyond Day –1**
- **Enrollment on a regimen where it will not be possible to achieve an 18 to 36 hour window between initiation of opebacan and infusion of donor cells**
- **Cord blood is the source of a subject’s transplanted cells**

Drug Administration

- All subjects will receive an initial 4 mg/kg continuous IV infusion for 30 minutes followed immediately by a continuous infusion that varies, according to the dose group, in dose level and duration

Treatment Plan

Dose Group	No. of Subjects	Dose Regimen
1	6	4 mg/kg continuous IV infusion for 30 minutes followed immediately by 6 mg/kg/day continuous IV infusion for 3 days
2	6	4 mg/kg continuous IV infusion for 30 minutes followed immediately by 9 mg/kg/day continuous IV infusion for 3 days
3	6	4 mg/kg continuous IV infusion for 30 minutes followed immediately by 12 mg/kg/day continuous IV infusion for 3 days
4	6	4 mg/kg continuous IV infusion for 30 minutes followed immediately by 12 mg/kg/day continuous IV infusion for 7 days
5	6	4 mg/kg continuous IV infusion for 30 minutes followed immediately by 12 mg/kg/day continuous IV infusion for 14 days

Administration

- Opebacan should be brought to room temperature prior to infusion
- The study medication must be the sole agent administered in the chosen port during the course of the infusion protocol. The venous access port will not be heparinized, but may be flushed as necessary with physiologic saline for injection
- Any sign of a reaction at a site of infusion should be recorded on the subject's CRF and source document as an adverse experience
- If the infusion is interrupted, it should be resumed as soon as possible. However, if more than two hours has elapsed, call XOMA's Medical Monitor at (800) 858-2783 before restarting the infusion. Any unexpected attributes or appearance of the investigational drug should be reported to XOMA immediately

Scheduled Assessments

- Routine laboratory analyses, including chemistries, CBC, coagulation measures, and urinalysis will be performed locally at the study sites
- Surface markers requiring same day analysis (e.g., TLR4) will also be processed and analyzed locally, though in some circumstances these samples may be frozen, batched and sent to a central lab
- All other samples will be analyzed at a central lab

Study Design

- **All safety information for each dose group completed through Day 28 will be reviewed by the Data Safety Monitoring Board (DSMB)**

- **Dosing may begin for the next dose group if no more than one subject in the previous group has experienced a dose-limiting toxicity (DLT) and the the DSMB feels there are no clinically significant safety concerns at the previous dose level**

- **All subjects will have disease status, performance score, and cumulative aGvHD status confirmed on Day 100 (± 14 days)**

Study Design

- **Safety will be assessed**
  - **by pre- and post-treatment VS and lab assessments**
  - **recording of adverse clinical events, with an emphasis on infectious and non-infectious regimen-related toxicities**

- **PK data will be collected and analyzed**

- **If a subject in any cohort experiences graft failure, the subject's cohort will be expanded to 12 subjects. If a second graft failure occurs in the expanded cohort, the DSMB will determine whether the study should be stopped after reviewing all available information**

Scheduled Assessments

The following assessments will be performed daily from Day –1 until Day 49 or until the subject is released from the hospital, whichever occurs first:

- **Metabolic assessment=Intake and output, total days of TPN only**
- **First waking set of vital signs - HR, BP, RR, and Temp. If a subject experiences an SAE, VS must be taken more frequently**
- **The oral mucositis score must be recorded at prescreening and then daily from Day –1 until the condition resolves to grade 0. If oral mucositis reappears after resolving, daily assessments must be reinstituted through Day 49 or until the subject is released from the hospital, whichever occurs first**

Outcome and Measures

- **Biological and clinical activity will be assessed through an evaluation of the following outcome measures:**
  - **Time to engraftment, defined as the first of three consecutive measurements of ANC ≥ 500/μL**
  - **Inflammatory states such as presence of fever and infection and Inflammatory markers**
  - **Transplant-related complications including, but not limited to, aGvHD, inadequate oral intake, presence of oral mucositis, veno-occlusive disease of the liver, and idiopathic pulmonary fibrosis**

Outcome and Measures

- **Safety will be assessed by clinical and laboratory measures and recording of adverse clinical events, with an emphasis on infectious and non-infectious regimen-related toxicities**

- **All subjects must have samples for anti-opebacan antibody analysis drawn at prescreening. Samples will also be collected at Day 28 to detect whether subjects have developed anti-opebacan antibodies**

- **The pharmacokinetics of opebacan will be determined through the collection of PK samples from each subject throughout the study**
