# Supplementary material for: Pilot experience with opebacan/rBPI 21 in myeloablative hematopoietic cell transplantation
Source: F1000Res. 2015 Dec 21;4:1480. [Version 1] doi: 10.12688/f1000research.7558.1 (PMC4722698; doi:10.12688/f1000research.7558.1)
Supplement: Supplementary file 4 [file f1000research-4-8138-s0003.tgz › e1d94d2c-5ccb-4cd3-ae91-d5dadf9b63a1.pdf]

STATUS PAGE

PROTOCOL **05-127**

Closed To New Accrual

Closure Effective Date: 09/02/09

No new subjects may be enrolled in the study as described above. Any questions regarding this closure should be directed to the study's Principal Investigator

DANA-FARBER CANCER INSTITUTE
DANA-FARBER / HARVARD CANCER CENTER

Office for Human Research Studies

Protocol Front Sheet

DFCI Protocol No.: 05127

1. PROTOCOL TITLE AND VERSION

Title: LPS Directed Innate Immunity in HSCT Patients
Protocol Version No./ Date: 6/4/15
Sponsor Study Number: n/A

2. DF/HCC STUDY CONTACT INFORMATION

Primary Study Contact: Lisa Brennan RN **Email:** lisa_brennan@dfci.harvard.edu **Phone:** 632-3846

INVESTIGATORS: (List only those under DFCI IRB, *i.e.*, from institutions listed in Section 6 below)
Overall PI: Eva C. Guinan MD **Phone:** 632-4932 **Institution(s):** DFCI
Site Responsible PI: **Phone:** **Institution(s):**

3. DRUG / DEVICE INFORMATION N/A:

☐ **Drug(s), Biologic(s)**:
Provided by:
IND Exempt: ☐ -or-
IND#: **Holder Type:** [pull down]
IND Holder Name:

☐ **Device(s) Name**:
Provided by:
IDE Exempt: ☐ -or-
IDE #: **Holder Type:** [pull down]
IDE Holder Name:

4. PROTOCOL COORDINATION, FUNDING, PHASE, MODE, TYPE ETC.

Regulatory Sponsor:
DF/HCC Investigator

Cancer Related: [pull down] If yes:
Primary Disease Program:
[pull down]
or
Primary Discipline Based Program:
Transplant

Funding/Support (check all that apply)**:**
☐ Industry:
☐ Federal Organization:
Grant #:
☐ Internal Funding:
☐ Non-Federal:
☒ Other:

CTEP Study: No

Phase: N/A

Multi-Center (i.e., non-DF/HCC site participation)**:**
No

Protocol Type: Other
If Ancillary, provide parent protocol #:

Protocol Involves (check all that apply as listed in the protocol document, even if not part of the research but is mandated by the protocol document)**:**

☐ Chemotherapy
☐ Immunotherapy
☐ Surgery
☒ Bone Marrow/Stem Cell Transplant
☐ Cell Based Therapy
☐ Gene Transfer (use of recombinant DNA)
☐ Radiation Therapy
☐ Hormone Therapy
☐ Vaccine
☐ Data Repository
☐ Exercise/Physical Therapy
☐ Genetic Studies
☐ Human Material Banking
☒ Human Material Collection
☒ Medical Record Review
☐ Questionnaires/Surveys/Interviews
☐ Radiological Exams
☐ Required Biopsy Study
☐ Human Embryonic Stem Cell
☐ Quality of Life
☐ Other:

5. SUBJECT POPULATION (also applies to medical record review and specimen collection studies)

Total Study-Wide Enrollment Goal: 75 **Greater than 25% of the overall study accrual will be at DF/HCC:** ☒ Yes ☐ No
Total DF/HCC Estimated Enrollment Goal: 75 **Adult Age Range:** 18-65 **Pediatric Age Range:**
Will all subjects be recruited from pediatric clinics? ☐ Yes ☒ No
If enrolling both adults and pediatric subjects, anticipated percent of pediatric subjects:
Retrospective Medical Record Reviews only (Please provide date range): from to

6. DF/HCC PARTICIPANTS UNDER DFCI IRB (check all that apply)

☐ Beth Israel Deaconess Medical Center (BIDMC)
☐ Beth Israel Deaconess Medical Center – Needham (BIDMC-Needham)
☐ Boston Children's Hospital (BCH)
☒ Brigham and Women's Hospital (BWH)
☒ Dana-Farber Cancer Institute (DFCI)
☐ Dana-Farber/New Hampshire Oncology-Hematology (DFCI @ NHOH)
☐ DF/BWCC in Clinical Affiliation with South Shore Hospital (DFCI @ SSH)
☐ Dana-Farber at Milford Regional Cancer Center (DFCI @ MRCC)
☐ Dana-Farber at Steward St. Elizabeth's Medical Center (DFCI @ SEMC)
☐ Massachusetts General Hospital (MGH)
☐ Mass General/North Shore Cancer Center (MGH @ NSCC)
☐ Mass General at Emerson Hospital – Bethke (MGH @ EH)
☐ New England Cancer Specialists (NECS)

7. NON-DF/HCC PARTICIPANTS UNDER DFCI IRB (check all that apply)

☐ Cape Cod Healthcare (CCH)
☐ Lowell General Hospital (LGH)
☐ New Hampshire Oncology-Hematology-P.A. (NHOH)
☐ Newton-Wellesley Hospital (NWH)
☐ Broad Institute
☐ Lawrence & Memorial Cancer Center in affiliation with Dana-Farber Community Cancer Care (LMCCC)

Protocol Front Sheet

8. DF/HCC INITIATED STUDIES ONLY - INSTITUTIONAL PARTICIPANTS UNDER OTHER IRB (N/A:)

DF/HCC Multi-Center Protocols: (list institution/location)

DF/PCC Network Affiliates: (list institution/location)

Protocol Number: 05-127

Approval Date: ____04/20/05_____(IRB meeting date when protocol/consent approved or conditionally approved)

Activation Date: ____04/21/05_____(Date when protocol open to patient entry)

Approval signatures are on file in the Office for Human Research Studies, tel. 617-632-3029.

Date Posted	Revised Sections	IRB Approval Date	OHRS Version Date
07/12/05	Front Sheet Replaced due to Amendment # 1	06/27/05	-
07/12/05	Protocol and Consent Forms Replaced due to Amendment # 2	07/11/05	-
9/1/05	Front Sheet Replaced due to Amendment # 3	8/31/05	-
9/30/05	Protocol Replaced –Amendment #4	9/26/05	-
10/19/05	Protocol and Front Sheet replaced due to Amendment # 5	10/18/05	-
1/23/06	Front Sheet, Protocol, Consent Form – Amendment #6	1/19/06	-
04/18/06	Consent Forms (footer only) replaced due to Continuing Review #1	04/13/06	-
04/26/06	Consent Forms, Protocol & Front Sheet Replaced due to Amendment #7	04/25/06	-
04/12/07	Consent Forms replaced due to Continuing Review #2	04/12/07	-
03/20/08	Front Sheet replaced due to Amendment #8	03/19/08	-
04/15/08	On Hold Alert Page posted due to Continuing Review pending; All research must stop	N/A	-
04/16/08	On Hold removed; Consent Forms (footer) replaced due to Continuing Review #3	04/16/08	-
10/09/08	Protocol, Consent Forms and Front Sheet replaced due to Amendment #9	10/08/08	-
03/18/09	Study renewal due to Continuing Review #4	03/13/09	-
12/08/09	Front Sheet replaced due to Amendment #10	11/25/09	-
03/12/10	Closure to accrual (effective 09/02/09) and Study Renewal due to Continuing Review #5	03/11/10	-
03/01/11	Study renewal/ Consent Forms footers and Front Sheet replaced due to Continuing Review #6	02/26/11	-
09/22/11	Front Sheet replaced due to Amendment #11	09/14/11	-
03/06/12	Study renewal/ Consent Forms footers replaced due to Continuing Review #7	02/20/12	-
01/29/13	Study renewal/ Consent Forms footers replaced due to Continuing Review #8	01/29/13	N/A
01/02/15	Study renewal/ Consent Forms footer replaced due to Continuing Review #9	12/31/14	N/A
07/13/15	Protocol and Front Sheet replaced due to Amendment #12	06/23/15	N/A

LPS-Directed Innate Immunity in Patients Undergoing Hematopoietic Stem Cell Transplantation (HSCT)

Investigator:

Primary: Eva C. Guinan, M.D.

Sub investigators: Ofer Levy M.D., Ph.D

Sites:

Dana-Farber Cancer Institute

44 Binney Street

Boston, MA 02115

Institutional Review Board/Ethics Review Committee:

Dana-Farber Cancer Institute

44 Binney Street

Boston, MA 02115

Version 6/4/15

TABLE OF CONTENTS

	Page
1. Abstract	3
2. Background/rationale	3
3. Objectives/study aims	4
4. Eligibility/subject enrollment	5
5. Study design/duration	5
6. Specimen/data collection procedures	6
6.1 LPS and Innate Immunity-Related Assays	6
7. Laboratory/data analysis	7
8. Statistical considerations	7
8.1 Analysis plan	7
8.2 Sample size	8
9. Regulatory requirements	9
9.1 Informed consent	9
9.2 Patient confidentiality	9
10. List of references	9

1. Abstract

Acute graft-versus-host disease (GVHD) is a major complication of allogeneic hematopoietic stem cell transplantation (HSCT) that is mediated in large part by donor T-cells responding to host antigens. Increasing evidence suggests that one trigger for acute GVHD [11] is Gram-negative bacterial lipopolysaccharide (LPS or endotoxin) stimulation of monocyte release of pro-inflammatory cytokines. This in turn induces donor T cell activation [1]. We have recently shown that patients undergoing HSCT have measurable endotoxin in their systemic circulation and increased plasma concentrations of the LPS-binding protein (LBP) [4]. In humans, correlations have been detected between major complications of HSCT and increases of LPS concentrations [4] and TNF-α serum levels [12, 13]. Characterizing novel early inflammatory events in the innate immune pathway that trigger acute GVHD holds promise in devising strategies to target that response for early diagnosis, new therapeutic interventions and GVHD prevention. The main objective of this study is to determine the feasibility of measuring elements of the LPS-directed innate immune response, including expression of TLR4/CD14 lipopolysaccharide (LPS or endotoxin) receptor and of LPS-induced cytokine release from antigen-presenting cells (APC), including monocytes and dendritic cells, in the peripheral blood of patients undergoing myeloablative allogeneic HSCT prior to and at engraftment.

2. Background and rationale

Acute graft-versus-host disease is a major complication of allogeneic hematopoietic stem cell transplantation that is mediated by donor T-cells responding to host antigens. Evidence is accumulating that acute GVHD is triggered by Gram-negative bacterial lipopolysaccharide (LPS or endotoxin) that induces monocytes to release pro-inflammatory cytokines consequently activating donor T cells [1]. LPS can enter the blood stream either by translocation across the damaged mucosa and/or as a result of invasive infection with Gram-negative bacteria. Murine studies indicate that radiation therapy and myeloablative conditioning regimens compromise mucosal integrity and leukocyte-based host defense thereby allowing translocation of LPS from endogenous bowel flora across the damaged mucosal barrier into the systemic circulation [2, 3]. We have recently shown that human patients undergoing HSCT have measurable endotoxin in their systemic circulation and increased plasma concentrations of the LPS binding protein (LBP) [4]. There is strong evidence that such gut-derived endotoxemia following myeloablative chemotherapy is a key trigger, via induction of the Th1-type cytokine tumor necrosis factor-α (TNF-α), of acute GVHD [2]. Conversely, anti-inflammatory/Th2-type cytokines such as IL-10, reduce acute GVHD [5].

Great advances have been made over the past decades in characterizing innate immune recognition of microbial products, including LPS, via *Toll*-like receptors expressed on monocytes and other host cells [6]. Human monocytes detect LPS via Toll-like receptor-4 (TLR4) and the glycosylphosphoinositide (GPI)-linked LPS co-receptor CD14 [7]. Upon activation of monocytes by LPS, surface expression of CD14 and

TLR4 are modulated: (a) surface expression of CD14 decreases due to shedding [8] and (b) surface expression of TLR 4 is up-regulated [9]. Although one study of TLR4 expression in patients undergoing HSCT found a correlation between hypomorphic alleles of the TLR4 gene and a lower subsequent risk of acute GVHD [10], little is known about the relationship between the functional expression of TLR4 and CD14 on human donor monocytes and the subsequent development of acute GVHD in HSCT recipients.

Studies using animal models have described an association of pro-inflammatory cytokine release with acute GVHD [11]. In humans, correlations have been detected between major complications of HSCT and increases of LPS concentrations [4] and TNF-α serum levels [12, 13]. However, the potential value of such markers of acute GVHD development remains to be determined. Of note, the expression TNF-α in intestinal biopsy tissue has been found to be highly specific for acute GVHD, but has not yet been associated with transplant-related mortality [14].

Characterizing early LPS-induced inflammatory events that trigger acute GVHD holds promise in devising new diagnostic and therapeutic strategies for post-transplant morbidity and mortality. We therefore propose a prospective, observational study of endotoxemia (i.e., entry of LPS into the bloodstream), and its relationship to cell surface characteristics and function of APC in the peripheral blood of children undergoing myeloablative allogeneic bone marrow transplant. It is anticipated that this study will help characterize the relationship of endotoxemia and modulation of APC expression of the LPS receptor components CD14 and TLR4 in transplant patients as well as begin to establish whether such events, and/or a donor phenotype characterized by high LPS-induced monocyte release of Th1-type cytokines, will correlate with subsequent development of acute GVHD in HSCT recipients. This may provide a basis for clinical studies in the areas of donor selection, early prediction of acute GVHD prior to its overt clinical manifestations, and eventually support the development of agents that modulate LPS-induced innate immune responses, including LPS-neutralizing proteins such as the neutrophil-derived bactericidal/permeability-increasing protein (BPI) [15] and/or inhibitory lipid A congeners [16].

3. Objectives/study aims

The specific objectives are:

1. To determine the feasibility of using *in vitro* measurements to examine LPS-induced markers of innate immunity in peripheral blood mononuclear cells derived from donors.
2. To develop preliminary data regarding markers of innate immune activation in blood leukocytes and tissue samples and in plasma from patients undergoing myeloablative allogeneic HSCT.

3. To develop preliminary evidence as to whether an association exists between specific markers of innate immunity, acute graft-versus-host-disease (aGVHD) and/or active infection.

4. Eligibility/subject enrollment

Adult patients 18-65 years of age undergoing any type of stem cell transplant, including autologous, allogeneic, non-myeloablative and myeloablative transplants, from any source including bone marrow(BM), peripheral blood stem cells (PBSC) or umbilical cord blood (UCB) and potentially their respective HSC donors will be offered participation in the study.

1. Patient and/or guardian and donor (if available and wants to participate) understand the procedures and agree to participate by providing informed consent.
2. In the case of NMDP donors (unrelated donors obtained from the National Marrow Donor Program), consent is obtained by an NMDP process based on their internal review and approval of this protocol.

5. Study design/duration

This is a prospective, observational study primarily designed to evaluate the feasibility of measuring cytokine release and monocyte expression of cytokine receptors in the peripheral blood of HSCT patients and, where available and enrolled, donors. The time points for blood sampling are based on our previously published results [4]. A total of 75 evaluable participants will be analyzed. The projected duration of this study will be 24-36 months. Patients will complete the blood collection schedule within the first 100 days after the transplant.

6. Specimen/data collection procedures

A total of 15-20cc (3-4 teaspoons**)** of peripheral blood in designated vacutainers will be obtained from any participating donors at a time-point prior to HSC donation. Similarly, 15-20 cc (3-4 teaspoons) of peripheral blood will be drawn into designated vacutainers from the HCST patients at time points listed below:

- Prior to initiation of transplant conditioning (baseline)
- D -3, -1, 0
- D+2, +4, 7 +/- one day
- D+14 +/-one day
- D+21 +/- one day

If the patient is discharged prior to any of the designated time points above, the sample will be drawn during the scheduled out patient clinic visit. If there are multiple clinic

visits, the sample will be drawn as close to or on the designated day (e.g., D7, D14, D21,).

Patients who are admitted to the hospital late in the day may have baseline blood work on the morning of the next day prior to initiation of chemotherapy. If GVHD develops within the first 100 days after transplant, an extra blood aliquot may be drawn within 4 days of diagnosis but it is not required.

If the patient or donor consents, any surplus blood may be saved for future research consistent with this study.

6.1 LPS and Innate Immunity-Related Assays

Assays will be performed on samples obtained at designated times stated in Section 6.0 and include, but are not limited to the following:

- Endotoxin related plasma studies, including but not limited to LPS, LBP and BPI
- Cell surface and functional studies which include flow cytometry of APCs not limited to surface staining for CD14 and TLR4
- Functional studies to include in vitro assay of LPS-induced TNF-α
- Cytokine studies of plasma levels, including but not limited to TNF-α and IL-10

Clinical nursing staff will draw samples that will be picked up and processed by research technicians under the supervision of Dr. Ofer Levy, Division of Infectious Diseases/Dept. Medicine/Children's Hospital. The specimens will be destroyed upon study completion.

Epidemiological information on donor and patient and clinical information the recipient for the first 100 days of the transplant course will be obtained through Dr. Eva Guinan from a registered HSCT Research Database (03-144), Dana-Farber clinic charts, and the Brigham and Women’s Hospital records. Worksheets will be developed by the study staff to record data on patient and donor characteristics and clinical data. We will collect and maintain patient data until the end of the research project. Some of this information may be collected from subjects' medical records after direct participation is ended. Information will be maintained by us until the completion of data analysis and the required regulatory reviews.

7. Laboratory/data analysis

Assays may include but not be limited to:

1. Whole blood anticoagulated with sodium heparin will be analyzed for basal and LPS-induced cytokine release by ELISA as described [17] or by flow cytometry

using a cytometric bead array (BD Biosciences). Whole blood TLR transcriptome analysis using RT-PCR will be performed.

2. Surface expression of innate immune receptors such as TLR4 and CD14 will be measured by flow cytometry as described [17].
3. Endotoxin (LPS) will be measured by chromogeneic assays
4. Plasma BPI and LBP will be measured by ELISA (HyCult Biotechnology).

8. Statistical considerations

8.1 Analysis plan

The first step in this largely descriptive analysis will be to assess the feasibility of measuring the cell surface markers (primarily CD14 and TLR4) at each time point. Feasibility will be assessed by whether or not there are a sufficient number of cells sampled to perform the assay. The percentage of patients with sufficient cells will be calculated at each time point.

We will then summarize the distributions of values from each assay at each time point using means, standard deviations, medians and other percentiles. Also of interest is the percent of patients with values below the limit of quantification. In addition to CD14 and TLR4, other assay measures in this analysis include plasma levels of LPS, LBP, BPI, TNF-α and IL-10, and in vitro assays of LPS-induced TNF-α from both the recipient (at each time point) and from any enrolled donor's cells. Changes over time will be assessed by calculating the within-person difference between two time points and assessing the significance of the change with the one-sample t-test (possibly after transformation to achieve approximate Normality) or the nonparametric Wilcoxon signed rank test. The data will also provide information on intra-class correlations, i.e., the within-person correlations over time, which will be valuable in the design of future studies.

Spearman correlations will be used to characterize the associations between the assay measurements at each time point. Primary interest here centers on the correlations between cell surface markers (CD14, TLR4) and plasma levels of LPS and TNF.

Finally, a preliminary assessment of associations between LPS-induced TNF-α (in donor cells and in recipient cells at each time point) and the occurrence of aGVHD will be conducted by describing the distributions of TNF-α levels separately in patients who develop vs. do not develop aGVHD. It is anticipated that approximately 40% of the patients will develop aGVHD. We will also test for the significance of these comparisons with the two-sample t-test (possibly after transformation to achieve approximate Normality) or the nonparametric two-sample Wilcoxon test. Similar analyses will be conducted for LPS, CD14, and TLR4.

Descriptive data including patient characteristics such as demographics, toxicity including aGVHD, engraftment, infection and laboratory data may be used from this non-interventional, observational study group to perform preliminary comparisons with cohorts from other transplant studies with similar patients and when similar measures are collected

8.2 Sample size

The target sample size is 50 evaluable patients. We anticipate that it will take about two-three years to accrue up to 60 patients (+/- donors), and that this will yield 50 participants with evaluable data out to Day 21 post-transplant. Twenty patients will yield 95% confidence intervals for a mean with half-width of 0.47 standard deviations and will provide 80% power for detecting significant correlations if the true correlation is at least $\pm$ 0.60. The study is not powered for the hypothesis tests described above. However, the sample size is adequate for assessing feasibility and for making initial descriptive assessments of the patterns of marker values over time and of correlations.

9. Regulatory requirements

9.1 Informed consent

Informed consent will be obtained from the patient or patient's legally authorized guardian/representative after adequate explanation of the aims, methods, anticipated benefits, and potential hazards of the study. The research subject will be given a copy of the signed informed consent document, and the original document will be retained in the CHB's medical records.

9.2 Patient confidentiality

Subjects will be identified by their initials and a study ID number only. Documents will be kept in confidence. Research staff listed on this protocol will use de-identified data for subject research records, for registering subjects, reporting on post-transplant complications, and maintaining information on the number and timing of sample collections.

10. List of references

1. Ferrara, J.L., K.R. Cooke, and T. Teshima, *The pathophysiology of acute graft-versus-host disease.* International Journal of Hematology, 2003. 78(3): p. 181-7.
2. Cooke, K., et al., *Tumor necrosis factor-a production to lipopolysaccharide stimulation by donor cells predicts the severity of experimental acute graft-versus-host disease.* J. Clin. Invest., 1998. 102: p. 1882-1891.
3. Cooke, K.R., et al., *The role of endotoxin and the innate immune response in the pathophysiology of acute graft versus host disease.* Journal of Endotoxin Research, 2002. 8(6): p. 441-8.

4. Levy, O., et al., *Endotoxemia and elevation of lipopolysaccharide-binding protein after hematopoietic stem cell transplantation.* Pediatric Infectious Disease Journal, 2003. 22(11): p. 978-81.
5. Wang, X.N., et al., *Interleukin-10 modulation of alloreactivity and graft-versus-host reactions.* p. 772-8, 2002 Sep 27.
6. Akira, S., *Mammalian Toll-like receptors.* Curr Op Immunol, 2003. 15: p. 5-11.
7. Ulevitch, R.J. and P.S. Tobias, *Recognition of gram-negative bacteria and endotoxin by the innate immune system.* Current Opinion In Immunology, 1999. 11(1): p. 19-22.
8. Bazil, V. and J.L. Strominger, *Shedding as a mechanism of down-modulation of CD14 on stimulated human monocytes.* Journal of Immunology., 1991. 147(5): p. 1567-74.
9. Marsik, C., et al., *Endotoxaemia modulates Toll-like receptors on leucocytes in humans.* British Journal of Haematology. 121(4), 2003: p. 653-6.
10. Lorenz, E., et al., *Association of TLR4 mutations and the risk for acute GVHD after HLA-matched-sibling hematopoietic stem cell transplantation.* Biology of Blood & Marrow Transplantation, 2001. 7(7): p. 384-7.
11. Schmaltz, C., et al., *Donor T cell-derived TNF is required for graft-versus-host disease and graft-versus-tumor activity after bone marrow transplantation.* Blood. 101(6), 2003: p. 2440-5.
12. Holler, E., et al., *Increased serum levels of tumor necrosis factor alpha precede major complications of bone marrow transplantation.* Blood, 1990: p. 1011-6, 1990 Feb 15.
13. Hildebrandt, G.C., et al., *Donor-derived TNF-alpha regulates pulmonary chemokine expression and the development of idiopathic pneumonia syndrome after allogeneic bone marrow transplantation.* Blood, 2004: p. 586-93.
14. Socie, G., et al., *Prognostic value of apoptotic cells and infiltrating neutrophils in graft-versus-host disease of the gastrointestinal tract in humans: TNF and Fas expression.* Blood, 2004: p. 50-7, 2004 Jan 1.
15. Levy, O., *Antimicrobial proteins and peptides: Anti-infective molecules of mammalian leukocytes.* J Leuk Biol, 2004. 76(5): 909-25.
16. Mullarkey, M., et al., *Inhibition of endotoxin response by e5564, a novel Toll-like receptor 4-directed endotoxin antagonist.* Journal of Pharmacology & Experimental Therapeutics, 2003. 304(3): p. 1093-102.
17. Levy, O., et al., *Selective impairment of Toll-like receptor-mediated innate immunity in human newborns: Neonatal blood plasma reduces monocyte TNF-alpha induction by bacterial lipopeptides, lipopolysaccharide, and imiquimod but preserves response to R-848.* J Immunol, 2004. 173: p. 4627–4634.
