# Supplementary material for: Pilot experience with opebacan/rBPI 21 in myeloablative hematopoietic cell transplantation
Source: F1000Res. 2015 Dec 21;4:1480. [Version 1] doi: 10.12688/f1000research.7558.1 (PMC4722698; doi:10.12688/f1000research.7558.1)
Supplement: Peer review history

# Peer review: Referee response for version 1

**DOI:** 10.5256/f1000research.8138.r11661

**Published:** 2016-01-20

**Authors:** Brett P. Giroir

**Affiliations:** 1 Health Policy Institute, Texas Medical Center, Houston, TX, USA

This manuscript details the first human experience utilizing rBPI21 during myeloablative HSC. This work follows several earlier publications indicating a role for LPS and induced cytokines in GVHD or other adverse outcomes after transplant in preclinical models. Also, the authors have previously publicized a very important manuscript detailing the positive effects of rBPI21 in a mouse model of ablative radiation.   

The primary findings of this current study are that rBPI21 was safely tolerated in the small group of patients, and that there are suggestive findings of benefit when compared to a non-randomized but matched control population at the same institutions during the same time period. The authors adequately explain the limitations of the study design but also discuss the important implications of this study. The importance of this study is very high, since rBPI21 has a very clean safety profile in humans even during infusion into pediatric patients with septic shock, and could therefore represent a potential easily administered drug with a high therapeutic index in this population.

The authors could provide clarifying statements about the time course/kinetics of endotoxemia and LBP elevation in these patients. Are LPS levels high immediately post the ablation, or only become elevated upon infusion of cells, or later in the course? This would help explain the dosing regimen in the study.  

In the next study, would there be consideration of a second rBPI infusion if there is severe acute GVHD, or is the mechanism of action only prophylactic in nature and not therapeutic during early GVHD? 

Finally, although it is understood that there is concern over rBPI potential cardiotoxicity based on a high dose preclinical model, there was absolutely NO cardiotoxicity during human infusions, including into patients with septic shock - many of whom had pulmonary artery catheters and precise documentation of cardiac safety (no decrease in CI, SVRI, PVRI, etc.). To exclude a large group of BMT patients exposed to high dose anthracyclines (such as done in this study) may unnecessarily limit access to patients who might highly benefit from rBPI since GVHD is itself documented in BMT patients to induce significant diastolic dysfunction and cardiomyopathy - which could be even worse in patients with pre-existing low ejection fractions or high exposure to cardiotoxic chemotherapy.

I have read this submission. I believe that I have an appropriate level of expertise to confirm that it is of an acceptable scientific standard.

# Peer review: Referee response for version 1

**DOI:** 10.5256/f1000research.8138.r11664

**Published:** 2016-01-05

**Authors:** Alan Cross

**Affiliations:** 1 Center for Vaccine Development, School of Medicine, University of Maryland, Baltimore, MD, USA

This proof-of-concept clinical study reports that the peri-HCT administration of a neutrophil-derived protein, BPI, that has endotoxin-neutralizing and antibacterial properties, is well-tolerated and may hasten platelet engraftment and reduce regimen-related toxicities. They did not measure circulating endotoxin levels or inflammation markers (e.g. sCD14 or CRP) to support the rationale for this study, although they do cite a publication by one of the co-authors (Ref 3) that supports this hypothesis. They may want to cite earlier studies by Jonathan Cohen that shows anti-endotoxin antibodies prevent acute GVHD in a murine model.

I have read this submission. I believe that I have an appropriate level of expertise to confirm that it is of an acceptable scientific standard.
